# Focused deterrence strategies effects on crime: A systematic review

**DOI:** 10.1002/cl2.1051

**Published:** 2019-09-09

**Authors:** Anthony A. Braga, David Weisburd, Brandon Turchan

**Affiliations:** ^1^ School of Criminology and Criminal Justice Northeastern University Boston Massachusetts; ^2^ Institute of Criminology Hebrew University of Jerusalem Jerusalem Israel; ^3^ Department of Criminology, Law and Society George Mason University Fairfax Virginia

## 1 PLAIN LANGUAGE SUMMARY

### 1.1 Focused deterrence is associated with moderate reductions in crime

#### 1.1.1 The review in brief

A relatively small number of people, often involved in gangs and criminally‐active groups, are responsible for a disproportionate share of crime. Focused deterrence strategies attempt to reduce offending behavior for specific types of crime. Our review suggests that these strategies are associated with moderate overall reductions in crime. Crime is not displaced to other areas, rather it is more likely that there is a diffusion of crime control benefits to adjacent areas and socially‐connected groups of offenders.

### 1.2 What is this review about?

Crime is highly concentrated amongst a small number of highly‐active offenders. Focused deterrence strategies combine law enforcement, community mobilization, and social services in an attempt to reduce offending behavior for specific crime types. A key feature of this crime control strategy involves the direct communications of the consequences of continued criminal offending and the availability of social services to targeted subjects. This review examines whether focused deterrence reduces crime and considers how observed crime reduction effects may vary by the different types of focused deterrence strategies and program evaluation designs.

**What is the aim of this review?**

This Campbell systematic review examines the effects of focused deterrence on crime. The review summarizes and analyzes results from 24 quasi‐experimental evaluations of focused deterrence interventions, including 12 programs targeting criminally active gangs or groups, nine programs targeting open‐air drug markets, and three programs targeting high‐risk individual offenders. All but one of the studies are from the United States.

### 1.3 What studies are included?

A total of 24 studies of focused deterrence interventions were identified. All studies were published from 2001 to 2015. Twenty‐three studies were conducted in the United States and one in Scotland. None of the identified studies used a randomized controlled trial design.

### 1.4 What are the main findings of this review?

#### 1.4.1 Is focused deterrence an effective approach to reducing criminal offending among problem persons and groups?

Yes. The available evidence suggests an overall reduction in crime when focused deterrence strategies are used. The largest reductions are generated by focused deterrence strategies that target criminally active gangs or groups, followed by programs that target individual chronic offenders and drug market interventions.

#### 1.4.2 Do some programs work better than others?

Yes. Gang/group intervention programs generate the largest effects, followed by programs targeting high‐risk individuals, with the smallest effects generated by drug market intervention (DMI) programs. DMI programs are most likely to suffer implementation problems which reduce effectiveness.

#### 1.4.3 Does crime get displaced to other areas?

No. No studies found significant crime displacement effects into surrounding areas. There is some evidence of the diffusion of crime control benefits.

### 1.5 What do the findings of this review mean?

Findings from this review support the growing use of focused deterrence as a proactive crime reduction strategy. Practitioners and policy‐makers should continue to implement focused deterrence programs to address serious crime problems.

The number of studies included in the updated review is more than double the number of studies included in the previous iteration of the review. However, despite the increase in eligible studies, no evaluations utilized a randomized controlled trial design. The growth of focused deterrence warrants more methodologically rigorous program evaluations and further exploration into the specific components of the strategy in order to improve our understanding of how the program reduces crime.

### 1.6 How up‐to‐date is this review?

The review authors searched for studies up to October 2015.

## 2 BRIEF ABSTRACT

### 2.1 Background

Focused deterrence strategies, also known as “pulling levers” policing programs, have been increasingly implemented in the United States and other countries to reduce serious violent crime committed by gangs and other criminally active groups, recurring offending by highly active individual offenders, and crime and disorder problems generated by overt street‐level drug markets.

### 2.2 Objectives

To synthesize the extant evaluation literature and assess the effects of focused deterrence strategies on crime.

### 2.3 Search methods

Multiple search strategies were used to identify eligible studies. These strategies included a keyword search of online abstract databases, hand searches of relevant journals, consultation with policing experts, and searches of bibliographies of past narrative, empirical, and systematic reviews of police crime prevention efforts.

### 2.4 Selection criteria

Eligible studies had to evaluate programs with the core elements of a focused deterrence strategy present, use quasi‐experimental or randomized experimental designs, and report at least one crime outcome.

### 2.5 Data collection and analysis

Twenty‐four studies evaluating focused deterrence interventions were identified and full narratives of these studies were reported. A formal meta‐analysis was conducted to determine the crime prevention effects of the eligible studies.

### 2.6 Results

Our meta‐analysis suggests that focused deterrence strategies are associated with an overall statistically significant, moderate crime reduction effect. However, effect sizes varied by program type and were smaller for evaluations with more rigorous research designs.

### 2.7 Authors' conclusions

The available empirical evidence suggests these strategies may generate crime reduction impacts. However, more rigorous program evaluations are needed.

## 3 EXECUTIVE SUMMARY/ABSTRACT

### 3.1 Background

Focused deterrence strategies, also known as “pulling levers” policing programs, have been increasingly implemented in the United States and other countries to reduce serious violent crime committed by gangs and other criminally active groups, recurring offending by highly active individual offenders, and crime and disorder problems generated by overt street‐level drug markets. These strategies are framed by an action research model that is common to both problem‐oriented policing and public health interventions to reduce violence. Briefly, focused deterrence strategies seek to change offender behavior by understanding underlying crime‐producing dynamics and conditions that sustain recurring crime problems and implementing an appropriately focused blended strategy of law enforcement, community mobilization, and social service actions. Direct communications of increased enforcement risks and the availability of social service assistance to target groups and individuals are defining characteristics of focused deterrence strategies.

### 3.2 Objectives

To synthesize the extant evaluation literature and assess the effects of pulling levers focused deterrence strategies on crime.

### 3.3 Search methods

Several strategies were used to perform an exhaustive search for literature fitting the eligibility criteria. First, a keyword search was performed on an array of online abstract databases. Second, we reviewed the bibliographies of past narrative and empirical reviews of literature that examined the effectiveness of pulling levers focused deterrence programs. Third, we performed forward searches for works that have cited the original focused deterrence review and seminal focused deterrence studies. Fourth, we searched bibliographies of narrative reviews of police crime prevention efforts and past completed Campbell systematic reviews of police crime prevention efforts. Fifth, we performed hand searches of leading journals in the field.

### 3.4 Selection criteria

Eligible studies had to meet three criteria: (a) the program had to have the core elements of a focused deterrence strategy present; (b) a comparison group was included, or a one‐group‐only interrupted time‐series design was used; (c) at least one crime outcome was reported. The units of analysis had to be people or places.

### 3.5 Data collection and analysis

Twenty‐four studies evaluating focused deterrence interventions were identified and full narratives of these studies were reported. All selected studies used quasi‐experimental designs. A formal meta‐analysis was conducted to determine the crime prevention effects of the eligible studies. Random effects models were used to calculate mean effect sizes.

### 3.6 Results

Nineteen of the 24 evaluations of focused deterrence strategies reported at least one noteworthy crime reduction effect associated with the approach. It is important to note here that, even with the addition of 14 recent evaluations to this updated review, none employed a randomized controlled trial design to evaluate the intervention. Our meta‐analysis suggests that pulling levers focused deterrence strategies are associated with an overall statistically significant, moderate crime reduction effect. However, program effect sizes varied by program type and was smaller for evaluations with more rigorous research designs.

### 3.7 Authors' conclusions

The available empirical evidence suggests these strategies may generate crime reduction impacts. These encouraging results suggests that policymakers and practitioners should continue to implement these programs to control serious crime problems. However, investments still need to be made to strengthen the overall rigor of program evaluations and improve our understanding of key program activities associated with observed crime reduction impacts.

## 4 BACKGROUND

### 4.1 The intervention

The focused deterrence approach is consistent with recent theorizing about police innovation, which suggests approaches that seek to both create more focus in the application of crime prevention programs and expand the tools of policing that are likely to be most successful in controlling crime (Weisburd & Eck, 2004). Focused deterrence interventions are aimed at influencing the criminal behavior of individuals through the strategic application of enforcement, community, and social service resources to facilitate desirable behaviors. These strategies are often framed as problem‐oriented exercises where specific recurring crime problems are analyzed, and responses are highly customized to local conditions and operational capacities. Focused deterrence allows police to increase the certainty, swiftness, and severity of punishment in innovative ways.

In an earlier version of this Campbell Collaboration systematic review, three basic kinds of focused deterrence programs were identified (Braga & Weisburd, 2012, 2011). The first type draws on the model of the Boston Operation Ceasefire experience during the 1990s (see Braga, Kennedy, Waring, and Piehl, 2001; Kennedy, Piehl, & Braga, 1996). This approach is focused on *gang and criminally active group violence reduction strategies.* It joins criminal justice agencies, social service organizations, and community members to engage directly with violent groups, communicate credible moral and law enforcement messages against violence clearly, make genuine offers of help for those who want it, and launch strategic enforcement campaigns against those who continue their violent behavior.

The second type of focused deterrence strategy is intended to reduce crime driven by street‐level drug markets and is generally called a “drug market intervention” (DMI) program. DMI‐focused deterrence strategies are used to identify street‐level dealers, immediately apprehend violent drug offenders, and suspend criminal cases for nonviolent dealers (Kennedy, 2008). DMI strategies then bring together nonviolent drug dealers, their families, law enforcement and criminal justice officials, service providers, and community leaders for a meeting that communicates directly to offenders that their drug dealing has to stop, the community cares for them but rejects their conduct, services, and job opportunities are available, and renewed dealing will result in the activation of the existing case (Kennedy & Wong, 2009).

Finally, some focused deterrence programs are aimed at preventing repeat offending by *high‐risk individuals*. In general, these strategies address the most dangerous offenders with a wide range of legal tools, put offenders on formal notice that their “next offense” will bring extraordinary legal attention, and focus community “moral voices” on such offenders to set a clear standard that violence is unacceptable (Deuchar, 2013; Kennedy, 2008; Papachristos, Meares, & Fagan, 2007).

### 4.2 How the intervention might work

There are several theoretical mechanisms underlying focused deterrence that shed insights on how the intervention might work in practice. There is ample skepticism in the literature regarding “person‐focused” approaches in policing (Weisburd, 2008). Such skepticism is rooted in evaluations of the standard model of policing dominant in the last century (National Research Council, 2004). In the standard model, the police focused on investigating and apprehending offenders. But the results of studies examining the crime prevention effects of strategies such as rapid response to calls for service (e.g., see Spelman & Brown, 1984), and investigations of crime after its occurrence (e.g., see Eck, 2002), led scholars to conclude that generalized person‐focused approaches were ineffective (National Research Council, 2004; Sherman et al., 1997; Telep & Weisburd, 2012; Weisburd & Eck, 2004). Even in the case of interventions directed at individuals, and including focused deterrence, Weisburd and Eck (2004, p. 53) concluded that the evidence for effectiveness was “weak.”

The theory is important to provide a strong logic model for effectiveness, especially when drawing a conclusion on the basis of non‐experimental evidence of program impacts. The strong theoretical model for the effectiveness of focused deterrence adds weight to the empirical evidence that we present in this article. Even though focused deterrence programs vary, they share common prevention mechanisms that are believed to influence crime. Although we do not evaluate these mechanisms directly, findings from evaluations of the associated programs provide insight into the effectiveness of these prevention mechanisms, which in turn yields knowledge that can aid in designing effective programs (Ludwig, Kling, & Mullainathan, 2011).

#### 4.2.1 Deterrence

Deterrence theory suggests that crime can be prevented when the costs of committing the crime are perceived by the offender to outweigh the benefits (Gibbs, 1975; Zimring & Hawkins, 1973). Most discussions of the deterrence mechanism distinguish between “general” and “special” deterrence (Cook, 1980). General deterrence is the idea that the general population is dissuaded from committing the crime when it sees that punishment necessarily follows the commission of a crime. Special deterrence involves punishment administered to criminals with the intent to discourage them from committing crimes in the future. Much of the literature evaluating deterrence has been focused on the effect of changing certainty, swiftness, and severity of punishment associated with certain acts on the prevalence of those crimes (Apel & Nagin, 2011; Nagin, 1998; Paternoster, 1987).

In addition to any increases in certainty, swiftness, and severity of sanctions associated with gun violence, focused deterrence strategies are intended to prevent crime through the advertising of the law enforcement strategy and the personalized nature of its application. The effective operation of general deterrence is dependent on the communication of punishment threats to relevant audiences. As Zimring and Hawkins (1973, p. 142) observed, “the deterrence threat may best be viewed as a form of advertising.” A key element of focused deterrence strategies involves the delivery of a direct and explicit “retail deterrence” message to a small target audience regarding what kind of behavior would provoke a special response and what that response would be. For instance, beyond the particular groups subjected to gang violence reduction interventions, the deterrence message was applied to a smaller specific audience (e.g., all gang‐involved youth in a particular city) rather than to a larger general audience, and it was operated by making explicit cause‐and‐effect connections between the behavior of the target population and the behavior of the authorities. Knowledge of what happened to others in the target population was intended to prevent further acts of violence by gangs in the jurisdiction.

The results of available research reveal that deterrent effects are ultimately determined by offender perceptions of sanction risk and certainty (Nagin, 1998). Durlauf and Nagin (2011, p. 40) observed that “[S]trategies that result in large and visible shifts in apprehension risk are most likely to have deterrent effects that are large enough not only to reduce crime but also apprehensions,” and they identified focused deterrence strategies as having these characteristics. As described earlier, focused deterrence strategies are targeted on specific behaviors by a small number of chronic offenders who are highly vulnerable to criminal justice sanctions. The approach directly confronts offenders and informs them that continued offending will not be tolerated and how the system will respond to violations of these new behavior standards. Face‐to‐face meetings with offenders are an important first step in altering their perceptions about sanction risk (Horney & Marshall, 1992; Nagin, 1998). As McGarrell, Chermak, Wilson, and Corsaro (2006) suggested, direct communications and affirmative follow‐up responses are the types of new information that may cause offenders to reassess the risks of continuing their criminal behavior.

In focused deterrence strategies, deterrent messages are framed to address the group context from which many crime problems emerge. The groups themselves can act as another internal communication vehicle for transmitting the actual sanction risk to other offenders. Sanctions for individual noncompliance are applied to groups; all communications to offenders focus on this group concept, with the thought that peer pressure will change individual and group behavior. As Braga and Kennedy (2012) described, meaningful enforcement actions and scrutiny by law enforcement agencies can leverage the rationality of group members to no longer encourage norms that provoke the outbreaks of violence. The citywide communication of the antiviolence message, coupled with meaningful examples of the consequences that will be brought to bear on groups that break the rules, can weaken or eliminate the “kill or be killed” norm as individuals recognize that their enemies will be operating under the new rules as well.

Changes in‐group norms and in objective risks associated with particular forms of misbehavior may, for example, make it more difficult to recruit peers for particular instances of co‐offending. Ethnographic research findings on illicit gun markets in Chicago have shown that gangs' assessment of the law enforcement responses to gun violence leads them to withhold access to firearms for younger and more impulsive members (Cook, Ludwig, Venkatesh, & Braga, 2007). DMI's goal of fundamentally disrupting overt drug markets can greatly enhance the difficulty of drug dealing: when buyers no longer routinely “cruise” once active markets, even a motivated street dealer may find it impossible to do business.

#### 4.2.2 Other theoretical perspectives

Many scholars have suggested there are other complementary violence reduction mechanisms at work in the focused deterrence strategies described here that need to be highlighted and better understood (Braga, 2012; Brunson, 2015; Corsaro & Engel, 2015). In Durlauf and Nagin's (2011) article, their focus is on the possibilities for increasing perceived risk and deterrence by increasing police presence. Nevertheless, in the focused deterrence approach, the emphasis is not only on increasing the risks associated with offending, but it is also on decreasing opportunity structures for crime, deflecting offenders away from crime, increasing the collective efficacy of communities, and increasing the legitimacy of police actions. Indeed, program designers and implementers sought to generate large crime reduction impacts from the multifaceted ways in which this strategy influences targeted offenders (Kennedy, 2011).

Discouragement emphasizes reducing the opportunities for crime and increasing alternative opportunity structures for offenders (Clarke, 1997). In this context, situational crime prevention techniques are often implemented as part of the core pulling levers work in focused deterrence strategies (Braga & Kennedy, 2012). Extending guardianship, assisting natural surveillance, strengthening formal surveillance, reducing the anonymity of offenders, and using place managers can greatly enhance the range and the quality of the varying enforcement and regulatory levers that can be pulled on offending groups and key actors in criminal networks. The focused deterrence approach also is aimed at redirecting offenders away from crime through the provision of social services and opportunities. Treated individuals are offered job training, employment, substance abuse treatment, housing assistance, and a variety of other services and opportunities.

Sampson, Raudenbush, and Earls (1997) emphasized the capacity of a community to realize common values and regulate behavior within it through cohesive relationships and mutual trust among residents. They argued that the key factor determining whether crime will flourish is a sense of the “collective efficacy” of a community. A community with strong collective efficacy is characterized as having high capacities for collective action for the public good. The use of focused deterrence strategies enhances collective efficacy in communities by emphasizing the importance of engaging and enlisting community members in the strategies developed. Implementation of the High Point DMI strategy, for example, drew on collective efficacy principles by engaging family, friends, and other “influential” community members in addressing the criminal behaviors of local drug dealers (Kennedy & Wong, 2009).

Community‐based action in focused deterrence strategies helps remove the justifications used by offenders to explain away their responsibility for the targeted behavior. In call‐ins and on the street, community members effectively invalidate the excuses for criminal behavior by challenging the norms and narratives that point to racism, poverty, injustice, and the like. In Boston, for example, Black clergy challenged gang members who attempted to use these excuses by countering that poverty, racism, and injustice were not linked to their decisions to fire shots in their neighborhoods and kill other young people who have experienced the same societal ills and life difficulties (Braga, Kennedy, Waring, & Piehl, 2001). Community members also work with law enforcement and social service agencies to (a) set basic rules for group‐involved offenders such as “don't shoot guns” and (b) alert the conscience of these offenders by appealing to moral values inherent in taking the life of another, causing harm to their neighborhood, or the pain that would be experienced by their mothers if they were killed or sent to prison for a long time in a far‐away location (Kennedy, 2011).

Finally, use of the focused deterrence approach takes advantage of recent theorizing regarding procedural justice and legitimacy. The effectiveness of policing is dependent on public perceptions of the legitimacy of police actions (Tyler, 2004). Legitimacy is the public belief that there is a responsibility and obligation to accept and defer voluntarily to the decisions made by authorities (Tyler, 2006). Findings from recent studies reveal that when procedural justice approaches are used by the police, citizens will not only evaluate the legitimacy of the police more highly, but they will also be more likely to obey the law in the future (Paternoster, Brame, Bachman, & Sherman, 1997; but see Nagin & Telep, 2017). Advocates of focused deterrence strategies argue that targeted offenders should be treated with respect and dignity (Kennedy, 2008, 2011), reflecting procedural justice principles. The Chicago Project Safe Neighborhood (PSN) strategy, for instance, was aimed at increasing the likelihood that the offenders would “buy‐in” and comply voluntarily with the prosocial, antiviolence norms being advocated by interacting with offenders in ways that enhance procedural justice in their communication sessions (Papachristos et al., 2007).

### 4.3 Why it is important to do the review

In the previous version of this Campbell Collaboration systematic review, 10 quasi‐experimental evaluations of the crime control impacts of focused deterrence programs were identified based on a search for eligible studies completed in 2010 (Braga & Weisburd, 2012, 2011). In that review, researchers found that focused deterrence strategies were associated with significant reductions in targeted crime problems. Although the authors concluded that the available evidence was highly supportive of crime reduction impacts (Braga & Weisburd, 2012, 2011), they noted the absence of randomized experiments and the fact that, in several of the included evaluations, weaker designs were used with nonequivalent comparisons.

The small number of studies and the preponderance of weaker evaluation designs, however, contribute to some healthy ongoing skepticism regarding the crime control benefits associated with focused deterrence programs among practitioners and crime policy scholars. The evaluation of the best‐known focused deterrence strategy, Boston's Operation Ceasefire (Braga et al., 2001; Piehl, Cooper, Braga, & Kennedy, 2003), has been greeted with both a healthy dose of skepticism (Fagan, 2002; Rosenfeld, Fornango, & Baumer, 2005) and some support (Cook & Ludwig, 2006; Morgan & Winship, 2007). The National Academy of Sciences' report on firearms data and research concluded that the Ceasefire quasi‐experimental evaluation was “compelling” in associating the intervention with a 63% reduction in youth homicide in Boston (National Research Council, 2005, p. 10); however, the report also stated that the lack of a randomized controlled trial left some doubt over how much of the decline was due to Ceasefire relative to other rival causal factors. Despite this uncertainty over the impact of the Boston Ceasefire strategy on youth homicide, the focused deterrence framework has been applied in many U.S. cities through federally sponsored violence prevention programs such as the Strategic Alternatives to Community Safety Initiative and Project Safe Neighborhoods (Dalton, 2002).

Former New York City Mayor Rudy Giuliani criticized the “Boston Model” as not leading to lasting crime control gains in his 2001 farewell address (The New York Times, 2001). In an article published in *The New Yorker*, well‐respected deterrence scholar Professor Franklin Zimring is quoted as lamenting the lack of rigorous evaluations of focused deterrence programs and, when assessing the Boston experience, suggested, “Ceasefire is more of a theory of treatment rather than a proven strategy” (Seabrook, 2009, p. 37). Other criminologists seem unaware of the existing empirical evidence. For instance, in his 2013 summary of the crime prevention value of focused deterrence programs, former National Council on Crime and Delinquency president Barry Krisberg reported, “It certainly hasn't been effective so far, and there is no information suggesting it is effective” (as interviewed by KTVU, 2013).

Recently, more cities have tested the focused deterrence approach to control gang violence, disorderly drug markets, and repeat offender problems. The National Network for Safe Communities, an applied research project of the John Jay College of Criminal Justice, provides support to some 42 U.S. cities who are implementing some version of a focused deterrence strategy.^1^ A few other countries have started to test the approach. For instance, a focused deterrence program has been implemented targeting youth violence in Glasgow, Scotland (Deuchar, 2013). Police executives and other public officials in Eastern European and South American countries, such as Turkey and Brazil, have also explored the possibility of implementing focused deterrence strategies to control gang and group‐related violence in their cities (National Network for Safe Communities, 2013).

Given the growing popularity of focused deterrence programs and conflicting scholarly views on the crime reduction value associated with the approach, ongoing systematic review of rigorous program evaluations is necessary to keep policy and practice debates rooted in the most up‐to‐date and comprehensive scientific evidence.

## 5 OBJECTIVES

The objective of this review is to synthesize the existing published and non‐published empirical evidence on the effects of pulling levers focused deterrence strategies on crime and to provide a systematic assessment of the preventive value of this approach.

### 5.1 Methods

#### 5.1.1 Criteria for considering studies for this review

##### 5.1.1.1 Types of studies

To be eligible for this review, interventions had to include the key components of a focused deterrence strategy as described above. Randomized experimental and quasi‐experimental (nonrandomized) designs that compared pre and postintervention measures were eligible for inclusion in this review, though we did not identify any randomized experiments in our search (Campbell & Stanley, 1966; Shadish, Cook, & Campbell, 2002). Eligible quasi‐experiments used a comparison group or one‐group‐only interrupted time‐series design that controlled for extraneous factors to analyze variations in crime trends pre and postintervention.^2^

##### 5.1.1.2 Types of units of analysis

The units of analysis could be areas, such as cities, neighborhoods, or police beats, or persons.

##### 5.1.1.3 Types of interventions

To be eligible for this review, interventions had to be identified as a focused deterrence strategy. As described by Kennedy (2006, pp. 156–157), pulling levers operations have tended to follow this basic framework:

- Selection of a specific crime problem, such as youth homicide or street drug dealing.
- Assembling an interagency enforcement group, typically including police, probation, parole, state and federal prosecutors, and sometimes federal enforcement agencies.
- Conducting research, usually relying heavily on the field experience of front‐line police officers, to identify key offenders—and frequently groups of offenders, such as street gangs, drug crews, and the like—and the context of their behavior.
- Framing a special enforcement operation directed at those offenders and groups of offenders, and designed to substantially influence that context, for example by using any and all legal tools (or levers) to sanction groups such as crack crews whose members commit serious violence.
- Matching those enforcement operations with parallel efforts to direct services and the moral voices of affected communities to those same offenders and groups.
- Communicating directly and repeatedly with offenders and groups to let them know that they are under particular scrutiny, what acts (such as shootings) will get special attention when that has in fact happened to particular offenders and groups, and what they can do to avoid enforcement action. One form of this communication is the “forum,” “notification,” or “call‐in,” in which offenders are invited or directed (usually because they are on probation or parole) to attend face‐to‐face meetings with law enforcement officials, service providers, and community figures.

We used this basic framework to assist in our determination of whether particular programs followed the focused deterrence approach. It is important to note here, however, that certain programs that were determined to be eligible for this review did not necessarily follow the very specific pulling levers steps identified by Kennedy (2006). Focused deterrence strategies are often framed as problem‐oriented exercises where specific recurring crime problems are analyzed and responses are highly customized to local conditions and operational capacities. As such, we fully anticipated a variety of focused deterrence strategies to be identified by our systematic review.

##### 5.1.1.4 Types of outcome measures

Eligible studies had to measure the effects of the focused deterrence intervention on officially recorded levels of crime at places or crime by individuals. Appropriate crime measures included crime incident reports, citizen emergency calls for service, and arrest data. Particular attention was paid to studies that measured crime displacement effects and diffusion of crime control benefit effects (Clarke & Weisburd, 1994; Reppetto, 1976). The review considered all forms of displacement and diffusion reported by the studies (e.g., spatial, temporal, target, modus operandi). Also, assessed was the quality of the methodologies used to measure displacement and diffusion effects.

### 5.2 Search methods for identification of studies

Several strategies were used to perform an exhaustive search for literature fitting the eligibility criteria. First, a keyword search was performed on 15 online abstract databases. Second, we reviewed the bibliographies of past narrative and empirical reviews of the literature on the effectiveness of focused deterrence programs (Braga, 2012; Kennedy, 2008; National Research Council, 2004, 2005). Third, we performed forward searches for works that cited the original focused deterrence review (Braga & Weisburd, 2012, 2011) and seminal focused deterrence studies (Braga et al., 2001; Kennedy et al., 1996; McGarrell et al., 2006; Papachristos et al., 2007). Fourth, we searched bibliographies of narrative reviews of police crime prevention programs (Braga, 2008a; Gravel, Bouchard, Descormiers, Wong, & Morselli, 2013; Koper, Woods, & Kubu, 2013; McGarrell et al., 2013; Petrosino et al., 2015; Sherman, 2002; Weisburd & Eck, 2004; Werb et al., 2011) and past completed Campbell systematic reviews of police crime prevention efforts (Bowers, Johnson, Guerette, Summers, & Poynton, 2011; Braga, Papachristos, & Hureau, 2014; Koper & Mayo‐Wilson, 2012; Mazerolle, Soole, & Rombouts, 2007; Weisburd, Telep, Hinkle, & Eck, 2008). Fifth, we performed hand searches of published articles in leading journals in the field.^3^ These searches were all completed between August 2015 and October 2015.

After finishing the above searches and reviewing the studies as described later, we emailed the list of studies meeting our eligibility criteria in December 2015 to leading criminal justice scholars knowledgeable in the area of focused deterrence strategies (see Appendix A). These 100 scholars were defined as those who authored at least one study which appeared on our inclusion list, anyone involved with U.S. National Research Council (2004, 2005) reviews of police research and firearms research, and other leading scholars identified by the authors. This helped us identify unpublished studies that did not appear in conventional databases or other reviews. Finally, we consulted with an information retrieval specialist at the outset of our review and at points along the way in order to ensure that appropriate search strategies were used to identify the studies meeting the criteria of this review.^4^

The following 15 databases were searched:

1. Sociological Abstracts
2. Criminal Justice Abstracts
3. National Criminal Justice Reference Service (NCJRS) Abstracts
4. Educational Resources Information Clearinghouse (ERIC)
5. Government Publications Office, Monthly Catalog (GPO Monthly)
6. Google Scholar
7. Proquest Dissertation and Theses A&I
8. West Law Next
9. Informit (includes CINCH)
10. Web of Science Core Collection
11. Academic Search Premier
12. HeinOnline
13. Social Sciences Premium Collection
14. The Grey Literature Database^5^
15. C2 SPECTR^6^

The following terms were searched in the 15 databases listed above:

1. Pulling levers AND police
2. Problem‐oriented policing
3. Police AND repeat offenders
4. Police AND gangs
5. Police AND guns
6. Gang violence prevention
7. Focused deterrence
8. Deterring violent offenders
9. Strategic gang enforcement
10. Crackdowns AND gangs
11. Enforcement swamping
12. Drug market intervention

### 5.3 Data collection and analysis

Two authors (Braga and Turchan) executed the varied search strategies to identify eligible studies. Abstracts that appeared to have a chance of fitting the eligibility criteria were added to a centralized list for further consideration. Once the initial search strategies were completed, the list of abstracts was jointly reviewed by two authors (Braga and Turchan). For abstracts that both reviewers believed had a reasonable likelihood of meeting the eligibility criteria, full‐text reports, journal articles, and books were obtained and analysed in‐depth. The third author (Weisburd) weighed in when there were any disagreements about the potential eligibility of a particular study.

#### 5.3.1 Details of study coding categories

All eligible studies were coded (see coding protocol in Appendix B) on a variety of criteria including:

a. Reference information (title, authors, publication, etc.)
b. Nature of description of a selection of site, problems, and so forth.
c. Nature and description of the selection of the comparison group or period
d. The unit of analysis
e. The sample size
f. Methodological type (randomized experiment or quasi‐experiment)
g. A description of the pulling levers intervention
h. Dosage intensity and type
i. Implementation difficulties
j. The statistical test(s) used
k. Reports of statistical significance (if any)
l. Effect size/power (if any)
m. The conclusions drawn by the authors

Braga and Turchan separately coded each eligible study. When coding issues emerged, they were discussed and resolved among three authors collectively (Braga, Weisburd, Turchan).

#### 5.3.2 Statistical procedures and conventions

Analysis of outcome measures across studies was carried out in a uniform manner and, when appropriate and possible, involved quantitative analytical methods. We conducted meta‐analyses of program effects to determine the size and direction of the effects and to weight effect sizes based on the variance of the effect size and the study sample size (Lipsey & Wilson, 2001). In this systematic review, the standardized mean difference effect size (also known as Cohen's *d*; see Rosenthal, 1994) was used. The Effect Size Calculator, developed by David B. Wilson and available on the Campbell Collaboration's web site, was used to calculate standardized mean difference effect sizes for reported outcomes in each study.^7^ Biostat's Comprehensive Meta‐Analysis Version 2.2 was then used to conduct the meta‐analysis of effect sizes. Computation of effect sizes in the studies was not always direct. The goal was to convert all observed effects into a standardized mean difference effect size metric. Indeed, it was sometimes difficult to develop precise effect size metrics from published materials. This reflects a more general problem in crime and justice with “reporting validity” (Farrington, 2006; Lösel & Köferl, 1989) and has been documented in reviews of reporting validity in crime and justice studies (see Perry & Johnson, 2008; Perry, Weisburd, & Hewitt, 2010).

Some studies reported Cohen's *d* as a key outcome measure (see, e.g., Braga, Hureau, & Papachristos, 2014; Saunders, Lundberg, Braga, Ridgeway, & Miles, 2015) and, after confirming that the studies used the appropriate methods, these effects sizes were included. In other evaluations, treatment and control group crime counts were used to calculate effect sizes for each study contrast. From these raw counts, Odds Ratios (ORs) were first calculated. The log of this OR was then multiplied by √3/π in order to attain the final effect size expressed as Cohen's *d* (see Hasselblad & Hedges, 1995). We then made an adjustment for over‐dispersion using the method in Farrington, Gill, Waples, and Argomaniz (2007). In a few studies, counts were not provided or could not be reconstructed from information in the study report. This was most often in papers that reported Incidence Rate Ratios (IRRs) in order to estimate treatment effects conditional on the use of covariates. In such cases, ORs were obtained by taking the product of the IRR and a ratio of the pretest means in the control and treatment group (OR = IRR × [mean_pre_C/mean pre_T]). This then allows *d* to be calculated from log OR using conventional methods. The standard error of this IRR is squared to obtain the variance. In the interrupted time series designs, we used standards and methods to estimate *d* as outlined by the Cochrane Group.^8^

#### 5.3.3 Determination of independent findings

One problem in conducting meta‐analyses in crime and justice is that investigators often do not prioritize outcomes examined. This is common in studies in the social sciences in which authors view the good practice as demanding that all relevant outcomes be reported. However, the lack of prioritization of outcomes in a study raises the question of how to derive an overall effect of treatment. For example, the reporting of one significant result may reflect a type of “creaming” in which the authors focus on one large and significant finding while ignoring the less positive results of other outcomes. But authors commonly view the presentation of multiple findings as a method for identifying the specific contexts in which the treatment is effective. When the number of such comparisons is small and therefore unlikely to affect the error rates for specific comparisons such an approach is often valid.

All studies were analyzed using three approaches. The first approach is conservative; we calculated an overall mean effect size for each study that combined all reported outcomes. The second represents the largest effect reported in the studies and offers an upper bound to the review findings. It is important to note that in some of the studies with more than one outcome reported, the largest outcome reflected what authors thought would be the most direct program effect. Finally, the smallest effect size for each study was analyzed. This approach is the most conservative and likely underestimates the effect of disorder policing programs on crime. It was used here primarily to provide a lower bound to the review findings. In short, using three approaches to testing program effects served as a sensitivity analysis to evaluate the effects of input variation on the output variation

## 6 RESULTS

### 6.1 Selection of studies

#### 6.1.1 Results of the search

Search strategies in the systematic review process generate a large number of citations and abstracts for potentially relevant studies that must be closely screened to determine whether the studies meet the eligibility criteria (Farrington & Petrosino, 2001). The screening process yields a much smaller pool of eligible studies for inclusion in the review. Combined with the results from the original review, the search strategies produced 62,541 distinct abstracts. The contents of these abstracts were reviewed for any suggestion of an evaluation of focused deterrence interventions. A total of 473 distinct abstracts were selected for further consideration. A joint review of these initially identified abstracts determined 131 abstracts had a reasonable likelihood of meeting the eligibility criteria and warranted in‐depth examination. For these 131 abstracts, full‐text reports, journal articles, and books were acquired then carefully assessed to determine whether the interventions involved focused deterrence strategies and whether the studies used randomized controlled trial designs or nonrandomized quasi‐experimental designs (excluded studies are reported in Appendix D). Twenty‐four eligible studies were identified and included in the updated review

1. Operation Ceasefire in Massachusetts (Braga et al., 2001)
2. Indianapolis Violence Reduction Partnership in Indianapolis, Indiana (McGarrell et al., 2006)
3. Operation Peacekeeper in Stockton, California (Braga, 2008b)
4. Project Safe Neighborhoods in Lowell, Massachusetts (Braga, Pierce, McDevitt, Bond, & Cronin, 2008)
5. Cincinnati Initiative to Reduce Violence in Cincinnati, Ohio (Engel, Corsaro, & Tillyer, 2010)
6. Operation Ceasefire in Newark, New Jersey (Boyle, Lanterman, Pascarella, & Cheng, 2010)
7. Operation Ceasefire in Los Angeles, California (Tita, Riley, & Greenwood, 2003)
8. Operation Ceasefire in Rochester, New York (Delaney, 2006)
9. Project Safe Neighborhoods in Chicago, Illinois (Papachristos et al., 2007)
10. Drug Market Intervention in Nashville, Tennessee (Corsaro & McGarrell, 2009)
11. Drug Market Intervention in Rockford, Illinois (Corsaro, Brunson, & McGarrell, 2009)
12. Drug Market Intervention in High Point, North Carolina (Corsaro, Hunt, Hipple, & McGarrell, 2012)
13. Drug Market Intervention in Peoria, Illinois (Corsaro & Brunson, 2013)
14. Operation Ceasefire II in Boston, MA (Braga et al., 2014)
15. Community Initiative to Reduce Violence in Glasgow, Scotland (Williams, Currie, Linden, & Donnelly, 2014)
16. Group Violence Reduction Strategy in Chicago, Illinois (Papachristos & Kirk, 2015)
17. Group Violence Reduction Strategy in New Orleans, Louisiana (Corsaro & Engel, 2015)
18. No Violence Alliance in Kansas City, Missouri (Fox, Novak, & Yaghoub, 2015)
19. Project Longevity in New Haven, Connecticut (Sierra‐Arevalo, Charette, & Papachristos, 2015)
20. Drug Market Intervention in Roanoke, Virginia (Saunders, Kilmer, & Ober, 2015)
21. Drug Market Intervention in Montgomery County, Maryland (Saunders et al., 2015)
22. Drug Market Intervention in Guntersville, Alabama (Saunders et al., 2015)
23. Drug Market Intervention in Seattle, Washington (Saunders et al., 2015)
24. Drug Market Intervention in Ocala, Florida (Saunders et al., 2015)

### 6.2 Characteristics of eligible studies

The 14 newly identified studies represent a large increase in eligible studies (140%) over the 10 evaluations considered in the previous systematic review. Table 1 summarizes the characteristics of the 24 selected studies. The selected studies examined focused deterrence interventions that were implemented in small, medium, and large cities. Only one study evaluated a focused deterrence program implemented in a jurisdiction outside the United States (Scotland). More than one‐third (*N *= 9, 37.5%) of the eligible studies were acquired through “grey literature” sources^9^ at the time the review of abstracts was completed.^10^ All 24 evaluations were released after 2000 and a half were completed after 2013. Half of the studies evaluated the crime reduction effects of focused deterrence strategies on serious violence generated by street gangs or criminally active street groups. Nine studies evaluated strategies focused on reducing crime driven by street‐level drug markets (Guntersville, High Point, Montgomery County, Nashville, Ocala, Peoria, Roanoke, Rockford, and Seattle) and three evaluated crime reduction strategies that were focused on individual repeat offenders (Chicago [PSN], Glasgow, and Newark).

**Table 1:** Characteristics of eligible focused deterrence evaluations (*N *= 24)

Characteristic	*N*	Percent
Country		
United States	23	95.8
Other (Scotland)	1	4.2
City population		
Small (<200,000 residents)	8	33.3
Medium (200,000–500,000 residents)	6	25.0
Large (>500,000 residents)	10	41.7
Study type		
Quasi‐experiment with matched comparison group	12	50.0
Quasi‐experiment with nonequivalent comparison group	9	37.5
Quasi‐experiment with no comparison group (ITS)	3	12.5
Intervention type		
Gang/group violence	12	50.0
Individual crime	3	12.5
Drug market	9	37.5
Displacement and diffusion		
Measured displacement/diffusion	5	20.8
Did not measure displacement/diffusion	19	79.2
Publication type		
Peer‐reviewed journal	15	62.5
Grey literature	9	37.5
Published report	2	8.3
Unpublished report	7	29.2
Completion year		
2001–2004	2	8.3
2005–2008	5	20.8
2009–2012	5	20.8
2013–2015	12	50.0

All eligible studies used quasi‐experimental designs to analyze the impact of focused deterrence strategies on crime. Half of the evaluations used quasi‐experimental designs with near‐equivalent comparison groups created through matching techniques. The Los Angeles evaluation used a quasi‐experimental design that included both nonequivalent and matched comparison groups; for the Los Angeles study, we only included the effects from the more rigorous matched comparison group analysis in our meta‐analysis. Nine evaluations (37.5%) used quasi‐experimental designs with nonequivalent comparison groups (Boston, Cincinnati, Indianapolis, Lowell, Nashville, New Haven, New Orleans, Rockford, and Stockton). The comparison units used in these evaluations were selected based on naturally occurring conditions, such as other cities or within‐city areas that did not receive treatment, rather than through careful matching to ensure comparability with treatment units. Three studies (12.5%) used one‐group‐only interrupted time‐series designs (Kansas City, Peoria, and Rochester). Table 2 provides a brief summary of the treatments, units of analysis, and research designs used by the 24 eligible studies.^11^

**Table 2:** Eligible focused deterrence evaluations

Study	Treatment	Units of Analysis	Research Design
Operation Ceasefire. Boston, MA. Braga et al. (2001)	Strategy focused on reducing serious violence by street gangs. 24‐month postintervention period (June 1996–May 1998). No threats to integrity of treatment noted during program implementation.	Citywide intervention. Outcome measures included monthly counts of citywide youth homicide incidents, citywide gun assault incidents, citywide shots fired calls for service, and youth gun assault incidents in one high‐risk district.	Nonequivalent quasi‐experiment comparing youth homicide trends in Boston relative to youth homicide trends in 39 other U.S. cities and 29 New England cities. Count‐based regression models controlling for trends and seasonal variations used to estimate the impact of intervention on time series.
Indianapolis Violence Reduction Partnership. Indianapolis, IN. McGarrell et al. (2006)	Strategy focused on reducing serious violence by street gangs. 27‐month postintervention period (April 1999–June 2001). No threats to integrity of treatment noted during program implementation.	Citywide intervention. Outcome measure was the monthly count of citywide homicides.	Nonequivalent quasi‐experiment comparing homicide trends in Indianapolis relative to homicide trends in six cities selected based on population and Midwestern location. ARIMA models controlling for trends and seasonal variations used to estimate impact of intervention on time series.
Operation Peacekeeper. Stockton, CA. Braga (2008b)	Strategy focused on reducing serious violence by street gangs. 65‐month postintervention period (September 1997–December 2002). No threats to integrity of treatment noted during program implementation.	Citywide intervention. Outcome measure was the monthly count of citywide gun homicides.	Nonequivalent quasi‐experiment comparing gun homicide trends in Stockton relative to gun homicide trends in eight cities selected based on population and California location. Count‐based regression models controlling for trends and seasonal variations used to estimate impact of intervention on time series.
Project Safe Neighborhoods. Lowell, MA. Braga et al. (2008)	Strategy focused on reducing serious violence by street gangs. 39‐month postintervention period (October 2002–December 2005). No threats to integrity of treatment noted during program implementation.	Citywide intervention. Outcome measure was the monthly count of fatal and nonfatal gun assault incidents.	Nonequivalent quasi‐experiment comparing gun assault trends in Lowell relative to gun assault trends in the State of Massachusetts and eight Massachusetts cities selected based on population, demographics, and yearly numbers of gun assaults. Count‐based and maximum‐likelihood regression models controlling for trends and seasonal variations used to estimate impact of intervention on time series.
Cincinnati Initiative to Reduce Violence. Cincinnati, OH. Engel et al. (2010)	Strategy focused on reducing serious violence by criminally active street groups. 37‐month postintervention period (October 2007–September 2009). No threats to integrity of treatment noted during program implementation.	Citywide intervention. Outcome measures were the monthly counts of citywide group member‐involved and nongroup member‐involved homicides.	Nonequivalent quasi‐experiment comparing group‐member‐involved homicide trends relative to nongroup‐member‐involved homicides. Count‐based regression models controlling for trends and seasonal variations used to estimate impact of intervention on time series.
Operation Ceasefire. Newark, NJ. Boyle et al. (2010)	Violence reduction strategy targeting individual gang members described as a “hybrid” between the Boston Ceasefire pulling levers strategy and the Chicago Ceasefire street worker program. 85‐week postintervention period (May 11, 2005–December 31, 2006). No threats to integrity of treatment noted during program implementation.	Intervention implemented in two square mile area that experienced elevated levels of gun violence. Outcome measure was the weekly number of gunshot wound incidents.	Near‐equivalent quasi‐experiment comparing gunshot wound trends in the targeted area relative to gunshot wound trends in a comparison area selected based on similar levels of gun violence, geographic size, and demographic characteristics. ARIMA models controlling for trends and seasonal variations used to estimate impact of intervention on time series. Used dual kernel density spatial analyses to examine the distribution of gunshot wound hot spots around target and comparison zones before and after the intervention was implemented.
Operation Ceasefire. Los Angeles, CA. Tita et al. (2003)	Strategy focused on reducing serious violence by criminally active street groups. Six‐month postintervention period (October 2000–February 2001). Evaluation team reported that integrity of the treatment was undermined due to a lack of commitment to the strategy by working group members and the unintended consequences of a police corruption scandal.	Intervention was implemented in a target area within the Boyle Heights neighborhood of Los Angeles. Outcome measures were monthly counts of violent crime incidents, gang crime incidents, and gun crime incidents.	Quasi‐experimental evaluation used two nonequivalent comparisons (the target area relative to the remainder of Boyle Heights; Boyle Heights relative to the surrounding larger Hollenbeck community) and one near‐equivalent comparison (Census block groups matched via propensity score analyses). A variety of regression‐based models were used to estimate the impact of the intervention on the distribution of monthly counts of the key outcome variables for 6‐month preintervention, 4 month suppression, and 2 month deterrence time periods. Examined immediate spatial displacement and diffusion effects in 11 Census block groups surrounding targeted Census block groups and gang crime committed by nontargeted gangs that were “socially tied” to targeted gangs.
Operation Ceasefire. Rochester, NY. Delaney (2006)	Strategy focused on reducing serious violence by street gangs and criminally active groups. 15‐month postintervention period (October 2003– December 2004). The treatment was undermined due to problems with interagency communication, limited enforcement actions, and inadequate delivery of the deterrence message.	Citywide intervention. Outcome measures were monthly counts of homicide, gun assault first degree, and gun robbery first degree, with a subanalysis on black male victims ages 15–30 for each outcome.	One‐group‐only interrupted time series evaluation comparing citywide outcome trends pre‐ and postintervention. Multiple regression models controlling for trends, seasonal variations, and lagged intervention effects, as well as changes in economic conditions and policing behavior, to estimate the impact of the intervention on the time series.
Project Safe Neighborhoods. Chicago, IL. Papachristos et al. (2007)	Gun violence reduction strategy comprised of four interventions: (a) increased federal prosecutions for convicted felons carrying or using guns, (b) lengthy sentences associated with federal prosecutions, (c) supply‐side firearm policing activities, and (d) social marketing of deterrence and social norms messages through offender notification meetings. 32‐month postintervention period (May 2002–December 2004). No threats to integrity of treatment noted during program implementation.	Intervention was implemented in two adjacent policing districts that experienced very high levels of homicide. Outcome measures were monthly and quarterly counts of homicides, gun homicides, gang homicides, and aggravated assault and battery incidents.	Quasi‐experimental evaluation comparing trends in targeted policing districts to trends in near‐equivalent policing districts matched via propensity score analysis. Hierarchical generalized linear growth curve regression models used to estimate impact of intervention on time series.
Drug Market Intervention. Nashville, TN. Corsaro and McGarrell (2009)	Strategy focused on reducing crime driven by street‐level drug market 14‐month postintervention period (March 2008–April 2009) No threats to integrity of treatment noted during program implementation	Intervention was implemented in the McFerrin Park neighborhood of Nashville. Outcome measures were monthly count of violent crime incidents, property crime incidents, illegal drug possession incidents, illegal drug equipment incidents, and total calls for service.	Nonequivalent quasi‐experimental design comparing trends in the intervention neighborhood to trends in the remainder of Davidson County. ARIMA models controlling for trends and seasonal variations used to estimate impact of intervention on time series. Examined immediate spatial displacement and diffusion effects in areas contiguous to the targeted neighborhood.
Drug Market Intervention. Rockford, IL. Corsaro et al. (2009)	Strategy focused on reducing crime driven by street‐level drug market. 14‐month postintervention period (May 2007–June 2008). No threats to integrity of treatment noted during program implementation.	Intervention was implemented in the Delancey Heights neighborhood of Rockford. Outcome measures were monthly count of violent crime incidents and nonviolent crime incidents.	Nonequivalent quasi‐experimental design comparing trends in the intervention neighborhood to trends in the remainder of Rockford. Hierarchical generalized linear growth curve regression models used to estimate impact of intervention on time series.
Drug Market Intervention. High Point, NC. Corsaro et al. (2012)	Strategy focused on reducing crime driven by street‐level drug market. 60‐month postintervention period from the year of the first implementation site (January 2004–December 2008). No threats to integrity of treatment noted during program implementation.	Intervention implemented in four neighborhoods. Outcome measure was the annual count of violent crime.	Quasi‐experimental evaluation comparing census blocks within the target area with matched comparison groups via propensity score analyses. Count‐based panel regression models with difference‐in‐difference estimators and place‐based and time‐varying fixed effects at the census block level. Examined immediate spatial displacement and diffusion effects in 59 adjacent Census blocks.
Drug Market Intervention. Peoria, IL. Corsaro and Brunson (2013)	Strategy focused on reducing crime driven by street‐level drug market. 13‐month postintervention period (November 2009–December 2010). Evaluation team reported that integrity of the treatment was undermined due to a lack of citizen involvement in and community awareness of the intervention.	Intervention was implemented in one neighborhood that had a disproportionately high number of crimes. Outcome measures included monthly counts of violent crime, property crime, drug and disorder crime, and total calls for service.	One‐group‐only interrupted time series evaluation comparing trends pre and postintervention for the target neighborhood. ARIMA models controlling for trends and seasonal variations used to estimate impact of intervention. Telephone surveys with residents in target area to determine their familiarity with the intervention and their perceived changes in neighborhood crime and disorder over the previous 6 months.
Operation Ceasefire II. Boston, MA. Braga et al. (2014)	Strategy focused on reducing serious violence by street gangs. 48‐month postintervention period (January 2007–December 2010). No threats to integrity of treatment noted during program implementation.	Citywide intervention targeted 19 gangs over the study period. Outcome measures included quarterly counts of victim gang‐involved shootings, suspect gang‐involved shootings, and total gang‐involved shootings.	Quasi‐experimental evaluation comparing trends for treated gangs to trends for untreated gangs matched via propensity score analyses. Negative binomial growth curve regression models with differences‐in‐differences estimators controlling for trends and seasonal variations to estimate the impact of intervention on time series. Displacement/diffusion effects measured for untreated “socially connected” gangs.
Community Initiative to Reduce Violence. Glasgow, Scotland. Williams et al. (2014)	Strategy designed to reduce physical violence and weapon carrying by gang youth. 35‐month postintervention period (October 2008–October 2011). No threats to integrity of treatment noted during program implementation.	Intervention implemented in two police divisions corresponding to the area of Glasgow. Outcome measures include annual counts of violent crime, nonviolent crime, physical violence crime, and weapon carrying crime.	Quasi‐experimental design comparing trends for one and 2‐year cohorts of targeted youth to matched comparison youth. Conditional fixed‐effects Poisson regression models including a group‐time period interaction term used to estimate impact of the intervention.
Group Violence Reduction Strategy. Chicago, IL. Papachristos and Kirk (2015)	Strategy focused on reducing serious violence by street gangs. 12‐month post‐call‐in evaluation period. No threats to integrity of treatment noted during program implementation.	Citywide intervention that targeted 149 gang factions. Outcome measures include the number of victimization, offending, and total shooting involvement for each faction.	Quasi‐experimental design postintervention shooting counts for treated gangs relative to postintervention shooting counts for untreated gangs matched via propensity score analyses. Difference‐of‐group means *Z*‐test comparison used to estimate impact of the intervention.
Group Violence Reduction Strategy. New Orleans, LA. Corsaro and Engel (2015)	Strategy focused on reducing serious violence by street gangs and criminally active groups. 17‐month postintervention period (November 2012–March 2014). No threats to integrity of treatment noted during program implementation.	Citywide intervention. Outcome measures include monthly counts of overall homicides, overall violent crime, overall property crime, firearm‐related homicides, firearm assaults, gang‐member‐involved homicides, and nongang member‐involved homicides.	Nonequivalent quasi‐experimental evaluation comparing homicide trends in New Orleans to 14 comparable cities and six high‐trajectory cities. Difference‐in‐difference count regression models used to compare homicide trends in New Orleans to nonequivalent controls with counterfactual tests.
No Violence Alliance. Kansas City, MO. Fox et al. (2015)	Strategy focused on reducing serious violence by street gangs and criminally active groups. 12‐month postintervention period. Early implementation was plagued by poor leadership and communication which delayed full implementation until nearly 1 year after the originally intended start day and those problems have been rectified.	Citywide intervention. Outcome measures include monthly counts of homicide and aggravated assault with a firearm.	One‐group‐only interrupted time series evaluation used to compare citywide trends in the preintervention period to 1, 3, 6, and 12 months postintervention time periods.
Project Longevity. New Haven, CT. Sierra‐Arevalo et al. (2015)	Strategy focused on reducing serious violence by street gangs and criminally active groups. 18‐month postintervention period (November 2012–April 2014). No threats to integrity of treatment noted during program implementation.	Citywide intervention. Outcome measures were the monthly counts of citywide total fatal and nonfatal shootings, group‐member‐involved shootings, and nongroup member‐involved shootings.	Non‐equivalent quasi‐experimental design comparing shooting trends in New Haven to a similar city (Hartford, CT). ARIMA models controlling for trends and seasonal variations used to estimate the impact of the intervention on the time series.
Drug Market Intervention. Roanoke, VA. Saunders et al. (2015)	Strategy focused on reducing crime driven by street‐level drug market. 12‐month postintervention period (Beginning December 2011 for Hurt Park and January 2013 for Melrose‐Rugby). The treatment was undermined due to a history of poor police‐community relations and lack of faith among residents in police despite the program's efforts to engage the community.	Intervention was implemented in the Hurt Park and Melrose‐Rugby neighborhoods. Outcome measures were 12‐month counts of total crime, violent crime, property crime, and drug crime.	Quasi‐experimental evaluation comparing trends in the targeted neighborhood to trends in comparison neighborhoods matched via synthetic control methods. Negative binomial regression models controlling for trends were used to estimate the impact of the intervention on the time series.
Drug Market Intervention Montgomery County, MD Saunders et al. (2015)	Strategy focused on reducing crime driven by street‐level drug market 12‐month postintervention period (March 2011–February 2012) The treatment was undermined due to a lack of community engagement	Intervention was implemented in the Damascus Gardens one‐square block apartment complex Outcome measures were 12‐month counts of total crime, violent crime, property crime, and drug crime	Quasi‐experimental evaluation comparing trends in the targeted neighborhood to trends in comparison neighborhoods matched via synthetic control methods Negative binomial regression models controlling for trends were used to estimate the impact of the intervention on the time series
Drug Market Intervention. Guntersville, AL. Saunders et al. (2015)	Strategy focused on reducing crime driven by street‐level drug market. 12‐month postintervention period (December 2011–November 2012). The treatment was undermined due to a lack of citizen involvement, as well as community distrust for police and faith leaders involved in the intervention.	Intervention was implemented in the Lakeview neighborhood. Outcome measures were 12‐month counts of total crime, violent crime, property crime, and drug crime.	Quasi‐experimental evaluation comparing trends in the targeted neighborhood to trends in comparison neighborhoods matched via synthetic control methods. Negative binomial regression models controlling for trends were used to estimate the impact of the intervention on the time series.
Drug Market Intervention. Seattle, WA. Saunders et al. (2015)	Strategy focused on reducing crime driven by street‐level drug market. 12‐month postintervention period (Beginning December 2009 for 23rd Street and January 2013 for International District). No threats to integrity of treatment noted during program implementation.	Intervention was implemented in the areas of the 23rd Street Corridor and International District. Outcome measures were 12‐month counts of total crime, violent crime, property crime, and drug crime.	Quasi‐experimental evaluation comparing trends in the targeted neighborhood to trends in comparison neighborhoods matched via synthetic control methods. Negative binomial regression models controlling for trends were used to estimate the impact of the intervention on the time series.
Drug Market Intervention. Ocala, FL. Saunders et al. (2015)	Strategy focused on reducing crime driven by street‐level drug market. 12‐month postintervention period (Beginning November 2009 for Second Chance and October 2010 for First Avenue). No threats to integrity of treatment noted during program implementation.	Intervention was implemented in the “Second Chance” neighborhood and the First Avenue housing project. Outcome measures were 12‐month counts of total crime, violent crime, property crime, and drug crime.	Quasi‐experimental evaluation comparing trends in the targeted neighborhood to trends in comparison neighborhoods matched via synthetic control methods. Negative binomial regression models controlling for trends were used to estimate the impact of the intervention on the time series.

Abbreviation: ARIMA, autoregressive integrated moving average.

The lack of randomized controlled trials is concerning, as well implemented randomized studies provide the strongest evidence of the causal impacts of programs or practices. Nonetheless, our review suggests that, in recent years, program evaluators have increasingly used more rigorous quasi‐experimental designs with matched comparison groups to estimate focused deterrence impacts. The previous iteration of this Campbell review (Braga & Weisburd, 2012, 2011) found that only 30% (3 of 10) eligible studies used quasi‐experimental designs with matched comparison groups. In contrast, 64.3% (9 of 14) of the newly identified studies in this updated review used these more rigorous controlled designs. While randomized experiments are sorely needed, the trend toward quasi‐experimental designs with higher levels of internal validity suggests reviewers can have more confidence in study findings on the effects of focused deterrence programs on crime.

The evolution of the rigor of the quasi‐experimental evaluation techniques is evidenced by the differing approaches used to evaluate separate implementations of the well‐known Boston Operation Ceasefire strategy in the 1990s and then in mid‐2000s. The U.S. Department of Justice (DOJ)‐sponsored evaluation of the impact of Operation Ceasefire in the 1990s used a nonrandomized quasi‐experimental design to compare youth homicide trends in Boston to youth homicide trends in other major cities in the United States and large New England cities (Braga et al., 2001; noted here as Boston Ceasefire I). The within‐Boston program impact assessment was supplemented by analyses of Ceasefire's effect on the monthly number of citywide gun assault incidents, citywide shots‐fired calls for service, and youth gun assault incidents in one high‐risk policing district. Count regression models, controlling for secular trends, seasonal variations, Boston youth population trends, Boston employment rate trends, robbery trends, adult homicide trends, and youth drug arrest trends, were used to estimate the effect of Ceasefire on the outcome variables. The impact of Ceasefire was estimated using a dummy variable to represent the commencement of the treatment time period. As noted in Table 3, the Boston Ceasefire I intervention was associated with a 63% decrease in youth homicides that was distinct from youth homicide trends in the comparison cities.

**Table 3:** Results of eligible focused deterrence evaluations

Study	Crime outcomes	Displacement/diffusion
Operation Ceasefire. Boston, MA. Braga et al. (2001)	Statistically significant 63% reduction in youth homicides, 25% reduction in gun assaults, 32% reduction in shots fired calls for service, and 44% reduction in youth gun assaults in one high‐risk district	Displacement/diffusion effects not measured
Indianapolis Violence Reduction Partnership. Indianapolis, IN. McGarrell et al. (2006)	Statistically significant 34% reduction in total homicide	Displacement/diffusion effects not measured
Operation Peacekeeper. Stockton, CA. Braga (2008b)	Statistically significant 42% reduction in gun homicide	Displacement/diffusion effects not measured
Project Safe Neighborhoods. Lowell, MA. Braga et al. (2008)	Statistically significant 44% reduction in gun assault incidents	Displacement/diffusion effects not measured
Cincinnati Initiative to Reduce Violence. Cincinnati, OH. Engel et al. (2010)	Statistically significant 35% reduction in group member‐involved homicides	Displacement/diffusion effects not measured
Operation Ceasefire. Newark, NJ. Boyle et al. (2010)	No statistically significant reduction in gunshot wound victims in target zone	The results of the displacement/diffusion analysis were inconclusive
Operation Ceasefire. Los Angeles, CA. Tita et al. (2004)	In Boyle Heights, gang crime decreased significantly compared with other regions of Hollenbeck during the suppression period of the intervention, and violent, gang, and gun crime all decreased significantly in the deterrence period. In the five targeted police reporting districts, violent crime decreased significantly in comparison with the rest of Boyle Heights in the suppression and the deterrence periods, and gang crime decreased significantly in the suppression period. In the Census block groups overlapping the targeted reporting districts, violent crime decreased significantly compared with the matched blocks.	Analyses suggested strong diffusion of crime control benefits into Census block groups immediately surrounding targeted area and a reduction in gang crime associated with the “socially tied” gangs.
Operation Ceasefire. Rochester, NY. Delaney (2006)	Statistically significant 25% reduction in homicide involving black male victims ages 15–30 and 27% reduction in gun robbery involving black male victims ages 15–30 at 1, 3, and 4 month lags. No significant reduction in total homicide and total gun violence, as well as gun assault involving black male victims ages 15–30.	Displacement/diffusion effects not measured
Project Safe Neighborhoods. Chicago, IL. Papachristos et al. (2007)	Statistically significant 37% reduction in total homicides reported in targeted police districts. Statistically significant reductions in gun homicides and aggravated assaults in targeted districts also reported. No statistically significant reduction in gang homicides in targeted police districts.	Displacement/diffusion effects not measured
Drug Market Intervention. Nashville, TN. Corsaro and McGarrell (2009)	Statistically significant 55% reduction in illegal drug possession offenses, 37% reduction in drug equipment offenses, and 28% reduction in property crimes reported in targeted neighborhood. No significant decreases reported in violent crime incidents and total calls for service.	Analyses suggested significant diffusion of crime control benefits into contiguous areas
Drug Market Intervention. Rockford, IL. Corsaro et al. (2009)	Statistically significant 22% reduction in nonviolent offenses. No significant decreases reported in violent offenses.	Displacement/diffusion effects not measured
Drug Market Intervention. High Point, NC. Corsaro et al. (2012)	Statistically significant 14% reduction in violent crime reported in target area	Analyses suggested a nonsignificant increase in violent crime in areas adjacent to target neighborhoods
Drug Market Intervention. Peoria, IL. Corsaro and Brunson (2013)	No statistically significant relationship with violent crime, property crime, drug/disorder crime, or total calls for service	Displacement/diffusion effects not measured
Operation Ceasefire II. Boston, MA. Braga et al. (2014)	Statistically significant 31% reduction in total gang‐involved shootings, 35% reduction in suspect gang‐involved shootings, and 27% in victim gang‐involved shootings among targeted gangs	Statistically significant 24% reduction in total gang‐involved shootings and 27% suspect gang‐involved shootings for vicariously treated gangs relative to matched comparison gangs (Braga et al., 2013)
Community Initiative to Reduce Violence. Glasgow, Scotland. Williams et al. (2014)	Statistically significant 65% and 84% reductions in weapon carrying among 1 and 2‐year targeted cohorts	Displacement/diffusion effects not measured
Group Violence Reduction Strategy. Chicago, IL. Papachristos and Kirk (2015)	Statistically significant 32% reduction in shooting victimization among targeted gangs relative to matched comparisons. Marginally significant 23% reduction in total shooting involvement among targeted gangs relative to matched comparisons.	Displacement/diffusion effects not measured
Group Violence Reduction Strategy. New Orleans, LA. Corsaro and Engel (2015)	Statistically significant 17% reduction in total homicides, 32% reduction in gang‐member‐involved homicides, 17% reduction in firearm homicides, and 17% reduction in nonfatal firearm assaults. No statistically significant relationship with nongang‐member‐involved homicides.	Displacement/diffusion effects not measured
No Violence Alliance. Kansas City, MO. Fox et al. (2015)	Statistically significant homicide reductions of 40% at one month, 34% at three months, and 29% at 6 months. Statistically significant gun‐involved aggravated assault reductions of 19% at one month and 14% at 3 months No statistically significant relationship with homicide at 12 months or gun‐involved aggravated assault at 6 and 12 months.	Displacement/diffusion effects not measured
Project Longevity. New Haven, CT. Sierra‐Arevalo et al. (2015)	Statistically significant 37% reduction in total shootings and homicides and 73% reduction in group‐member‐involved homicides and shootings	Displacement/diffusion effects not measured
Drug Market Intervention. Roanoke, VA. Saunders et al. (2015)	In the Hurt Park neighborhood, statistically significant 30% reduction in total crime at 3 months, 19% at 6 months, 28% at 9 months, and 23% at 12 months; statistically significant 45% reduction in property crime at 6 months, 57% at 9 months, and 50% at 12 months, as well as significant 24% reduction in violent crime at 3 months and 29% at 9 months. In the Melrose‐Rugby neighborhood, statistically significant 15% reduction in violent crime at 3 months and 34% at 6 months.	Displacement/diffusion effects not measured
Drug Market Intervention. Montgomery County, MD. Saunders et al. (2015)	No statistically significant reduction in total crime, violent crime, property crime, or drug crime	Displacement/diffusion effects not measured
Drug Market Intervention. Guntersville, AL. Saunders et al. (2015)	No statistically significant reduction in total crime, violent crime, property crime, or drug crime	Displacement/diffusion effects not measured
Drug Market Intervention. Seattle, WA. Saunders et al. (2015)	In the International District, statistically significant 15% reduction in total crime at 3 months and 6 months; statistically significant 8% reduction in property crime at 3 months and marginally significant 17% reduction at 6 months; statistically significant 53% reduction in violent crime at 3 months and marginally significant 40% reduction at 6 months, 34% reduction at 9 months, and 34% reduction at 12 months; statistically significant 29% reduction in drug crime at 3 months and marginally significant 17% reduction at 6 months. In the 23rd Street Corridor neighborhood, no statistically significant reduction in total crime, violent crime, property crime, or drug crime.	Displacement/diffusion effects not measured
Drug Market Intervention. Ocala, FL. Saunders et al. (2015)	No statistically significant reduction in total crime, violent crime, property crime, or drug crime for either intervention site	Displacement/diffusion effects not measured

The Boston Ceasefire I evaluation has been reviewed by a number of researchers and the relationship between program implementation and the subsequent trajectory of youth homicide in Boston during the 1990s has been closely scrutinized (see Fagan, 2002; Ludwig, 2005; Morgan & Winship, 2007; Rosenfeld et al., 2005). The U.S. National Research Council's (2005) Committee to Improve Research Information and Data on Firearms ultimately concluded that the Ceasefire I evaluation was compelling in associating the intervention with the subsequent decline in youth homicide. However, the Committee also suggested that many complex factors affect youth homicide trends and that it was difficult to specify the exact relationship between the focused deterrence intervention and subsequent changes in youth offending behaviors. While the Ceasefire I evaluation controlled for existing violence trends and certain rival causal factors, there could be complex interaction effects among these factors not measured by the evaluation that could account for some meaningful portion of the decrease. The evaluation was not a randomized controlled experiment. As such, the nonrandomized control group research design cannot rule out these internal threats to the conclusion that Ceasefire was the key factor in the youth homicide decline.

Braga et al. (2014) conducted a rigorous quasi‐experimental evaluation of a reconstituted Boston Ceasefire program implemented during the mid‐2000s in response to growing gang violence problem (noted here as Boston Ceasefire II). Propensity scores were used to match treated Boston gangs to untreated Boston gangs who were not connected to the treated gangs through rivalries or alliances. Differences‐in‐differences estimators in growth‐curve regression models were used to assess the impact of Ceasefire II by comparing gun violence trends for matched treatment gangs relative to matched comparisons gangs during 2006 through 2010 study period. The Ceasefire II evaluation reported that total shootings involving directly treated gangs were reduced by 31% relative to total shootings involving comparison gangs. It is important to note that the Ceasefire II evaluation yielded a much more conservative violence reduction estimate when compared to program impacts reported in the Ceasefire I quasi‐experimental evaluation.

Using the Maryland Scientific Methods Scale (Sherman et al., 1997) as a standard, the Ceasefire I impact evaluation would be considered a “Level 3” (in a five‐level scale) evaluation and also regarded as the minimum design that is adequate for drawing conclusions about program effectiveness. These designs rule out many threats to internal validity such as history, maturation/trends, instrumentation, testing, and mortality. However, as Farrington, Gottfredson, Sherman, and Welsh (2002) observe, the main problems of Level 3 evaluations center on selection effects and regression to the mean due to the nonequivalence of treatment and control conditions. The Ceasefire II evaluation would be considered a “Level 4” evaluation as it measured outcomes before and after the program in multiple treatment and control condition units. These types of designs have better statistical control of extraneous influences on the outcome and, relative to lower‐level evaluations, deals with selection and regression threats more adequately.

Five studies (20.8%) examined possible crime displacement and diffusion of crime control benefit impacts that may have been generated by the focused deterrence interventions. The High Point DMI, Nashville DMI, Newark Ceasefire, and Los Angeles Ceasefire evaluations tested whether areas proximate to treatment locations experienced changes in crime levels. The Los Angeles Ceasefire and Boston Ceasefire II examined whether the focused deterrence intervention influenced the criminal behavior of gangs socially connected to targeted gangs through rivalries and alliances.

Potential threats to the integrity of the treatment were noted in seven studies (29.2%). For instance, Tita et al. (2003) reported that the Los Angeles intervention was not fully implemented as planned. The implementation of the Ceasefire program in the Boyle Heights neighborhood of Los Angeles was negatively affected by the well‐known Ramparts LAPD police corruption scandal and a lack of ownership of the intervention by the participating agencies. During the initial implementation of the Kansas City No Violence Alliance group violence reduction strategy (VRS), Fox et al. (2015) reported a concerning lack of leadership and poor communication among partnering agencies; these issues were eventually addressed as the intervention continued to be implemented. Similarly, the Rochester Ceasefire group VRS was negatively impacted by problems with interagency communication that led to limited enforcement actions and inadequate delivery of the deterrence message to targeted groups (Delaney, 2006). DMI programs in Guntersville, Montgomery County, Peoria, and Roanoke were noted to suffer from a lack of community involvement in the targeted areas (Corsaro & Brunson, 2013; Saunders, Ober, Kilmer, & Greathouse, 2016).^12^

Table 3 summarizes the main effects of the intervention on crime outcomes and, when measured, crime displacement and diffusion of crime control benefits effects. A more detailed narrative review of the focused deterrence strategies contained in the eligible studies is provided in Appendix C.

Nineteen of the twenty‐four studies (79.2%) reported noteworthy crime reductions associated with the focused deterrence approach. (Table 3). All 12 studies evaluating the impacts of focused deterrence strategies on violence by gangs and criminally active groups reported at least one statistically significant crime control impact associated with program implementation. While a nonstatistically significant reduction in gunshot wound victimization in the target zone was noted, the evaluation of Newark's Operation Ceasefire did not report any statistically significant crime prevention benefits generated by focusing on individual violent gang members. The other four studies that did not report any noteworthy crime control impacts were DMI programs implemented in Guntersville, Montgomery County, Ocala, and Peoria.

Five studies conducted six tests of possible crime displacement and diffusion of crime control benefits associated with the evaluated focused deterrence programs (Table 3). These studies included gang/group violence reduction strategies (Boston II, Los Angeles), DMI programs (High Point, Nashville), and individual repeat offender strategies (Newark). Two of the four studies that measured whether crime levels were impacted in areas immediate proximate to treatment areas reported noteworthy diffusion of crime control benefits associated with the focused deterrence intervention (Nashville, Los Angeles); none reported significant crime displacement effects into surrounding areas.

Two focused deterrence studies investigated the existence of displacement and diffusion effects on the criminal behavior of gangs that were socially connected to targeted groups. The Los Angeles intervention targeted two rival gangs operating out of the same area (Hollenbeck). Criminal activity (e.g., violent, gang, and gun crimes) was substantially reduced among the two gangs over a 6‐month pre‐post period. Slightly larger reductions in these crimes were evident among four nontargeted, rival gangs in surrounding areas during the same time period. Part of the explanation for the diffusion effects may rest with fewer feuds between the targeted and nontargeted gangs. Tita et al. (2003) also speculated that diffusion effects may have been influenced by social ties among the targeted and rival gangs. This seemed to be especially the case for gang crimes involving guns. In a companion paper to the main effects program evaluation, Braga et al. (2013) found that the Boston Ceasefire II strategy also created spillover deterrent effects onto other gangs that were socially connected to targeted gangs through rivalries and alliances. Total shootings involving these “vicariously treated” gangs decreased by 24% relative to total shootings involving matched comparison gangs.

### 6.3 Risk of bias in included studies

Table 4 presents our assessment of the risk of bias in the *N *= 24 included focused deterrence studies. We assessed the level of risk of bias along with six sources of potential bias for each study (“Low” or “High”), or if a study was not clear on whether the bias was present or not (“Unclear”). The dimensions of bias assessed were: (a) To what extent was randomization absent in the allocation of study units? (b) How much did the assignment sequence stray from proper randomization protocol? (c) How dissimilar were treatment and control units at the baseline? (d) What level of contamination was present in the study? (e) To what degree did this study engage in selective reporting? (f) How much did other reported risks of bias threaten the integrity of this study?

**Table 4:** Assessment of risk of bias in eligible focused deterrence studies

Study (Author(s), Year)	Allocation method^a^	Assignment sequence^b^	Selection bias^c^	Contamination^d^	Reporting bias^e^	Other bias^f^
Boston Ceasefire I (Braga et al., 2001)	High	High	High	Low	Low	Low
Indianapolis (McGarrell et al., 2006)	High	High	High	Low	Low	Low
Stockton (Braga, 2008b)	High	High	High	Low	Low	Low
Lowell (Braga et al., 2008)	High	High	High	Low	Low	Low
Cincinnati (Engel et al., 2010)	High	High	High	Low	Low	Low
Newark Boyle et al. (2010)	High	High	Low	Low	Low	Low
Los Angeles (Tita et al., 2003)	High	High	Low	Low	Low	High
Rochester (Delaney, 2006)	High	High	High	Low	Low	High
Chicago PSN (Papachristos et al., 2007)	High	High	Low	Low	Low	Low
Nashville (Corsaro and McGarrell, 2009)	High	High	High	Low	Low	Low
Rockford (Corsaro et al., 2009)	High	High	High	Low	Low	Low
High Point (Corsaro et al., 2012)	High	High	Low	Low	Low	Low
Peoria (Corsaro and Brunson, 2013)	High	High	High	Low	Low	High
Boston Ceasefire II (Braga et al., 2014)	High	High	Low	Low	Low	Low
Glasgow (Williams et al., 2014)	High	High	Low	Low	Low	Low
Chicago GVRS (Papachristos and Kirk, 2015)	High	High	Low	Low	Low	Low
New Orleans (Corsaro and Engel, 2015)	High	High	High	Low	Low	Low
Kansas City (Fox et al., 2015)	High	High	High	Low	Low	High
New Haven (Sierra‐Arevalo et al., 2015)	High	High	High	Low	Low	Low
Roanoke (Saunders et al., 2015)	High	High	Low	Low	Low	High
Montgomery County (Saunders et al., 2015)	High	High	Low	Low	Low	High
Guntersville (Saunders et al., 2015)	High	High	Low	Low	Low	High
Seattle (Saunders et al., 2015)	High	High	Low	Low	Low	Low
Ocala (Saunders et al., 2015)	High	High	Low	Low	Low	Low
“High” Totals	24	24	12	24	24	7
% of *N* = 65 studies	100%	100%	50.0%	100%	100%	29.2%

^a^ To what extent was randomization absent in the allocation of study units?

^b^ How much did the assignment sequence stray from proper randomization protocol?

^c^ How dissimilar were treatment and control units at the baseline?

^d^ What level of contamination was present in the study?

^e^ To what degree did this study engage in selective reporting?

^f^ How much did other reported risks of bias threaten the integrity of this study?

As noted above, none of the 24 studies included in this review were randomized experiments. There were some limitations to the internal validity of the included quasi‐experimental studies. Half of all eligible studies (*N *= 12, 50.0%) provided direct evidence (usually in the form of a table that presented balanced outcomes and descriptive variables) that the treatment and control units were similar at the baseline measurement period. For instance, the Chicago GVRS evaluation used propensity scores to develop a balanced group of untreated gangs to compare to the treated gangs (Papachristos & Kirk, 2015). Twelve quasi‐experimental studies (50.0%) used treatment and control units that were not the same. For instance, the Indianapolis quasi‐experimental evaluation compared homicide trends in the city relative to homicide trends in other nonequivalent cities (McGarrell et al., 2006). Two studies used interrupted time series analyses without equivalent comparisons (Delaney, 2006; Fox et al., 2015).

None of the included studies reported any evidence of contamination of control conditions during the intervention period. Further, none of the included studies reported evidence suggestive that the evaluators were only selecting those crime types that showed an effect. Finally, only seven studies (29.2%) presented any other evidence of possible bias. As described earlier, these studies suggested that there were serious threats to the integrity of the treatments applied. The internal validity of the included studies was mixed. Among the eligible quasi‐experimental designs, the strength of the research design varied. Therefore, we conducted sensitivity analyses that tested the moderating effects of research design on the relationship between focused deterrence programs and crime outcomes.

### 6.4 Synthesis of results

#### 6.4.1 Meta‐analysis of the effects of focused deterrence on crime

**Figure 1:**
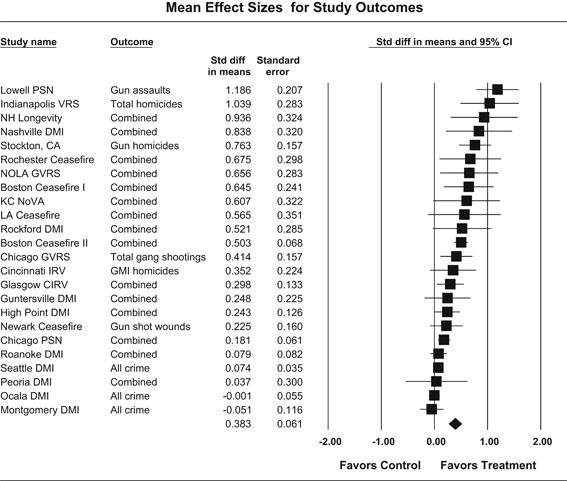
Mean effect sizes for study outcomes

Our meta‐analyses of the effects of focused deterrence programs on crime included all 24 eligible studies. Using the mean effect criterion for the eligible studies, the forest plot in Figure 1 shows the standardized difference in means between the treatment and control or comparison conditions (effect size) with a 95% confidence interval. Because the studies vary in their contexts and approaches, which is indicated by a significant *Q* statistic (*Q *= 122.568, *df* = 23, *p* < .05, Tau^2^ = 0.053), we used a random effects model to estimate the overall mean effect size. The meta‐analysis of effect sizes suggests a statistically significant effect in favor of focused deterrence strategies. The overall effect size for these studies was 0.383 (*p* < .05; 95% CI = 0.264, 0.503). This is below Cohen's (1988) standard of 0.50 for a medium effect size. Nonetheless, the overall effect size is relatively large compared to assessments of interventions in crime and justice work more generally (Lipsey, 2000; MacKenzie & Hickman, 1998; Weisburd, 1993; Weisburd et al., 2008).

**Figure 2:**
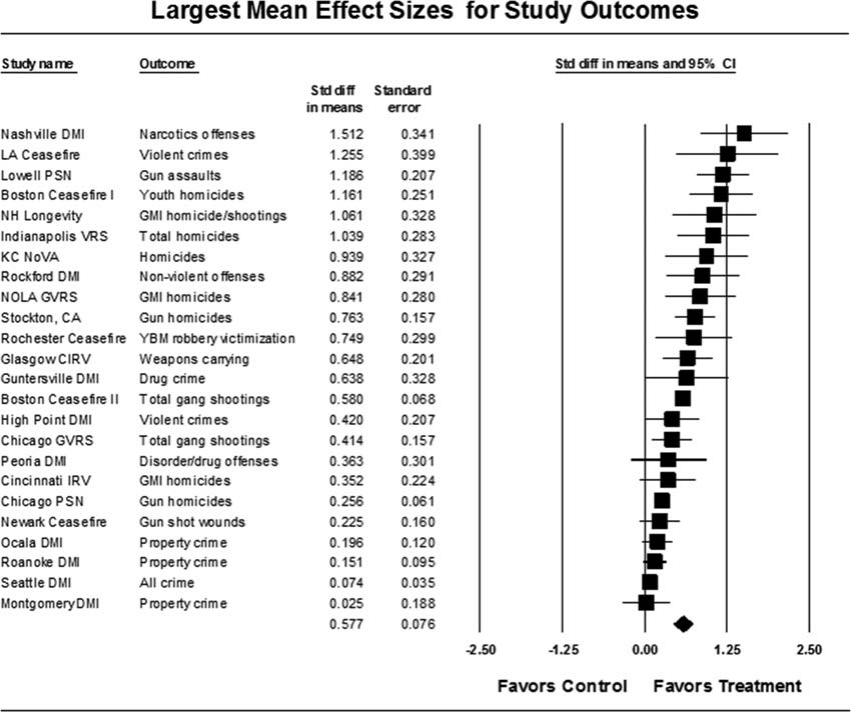
Largest mean effect sizes for study outcomes

**Figure 3:**
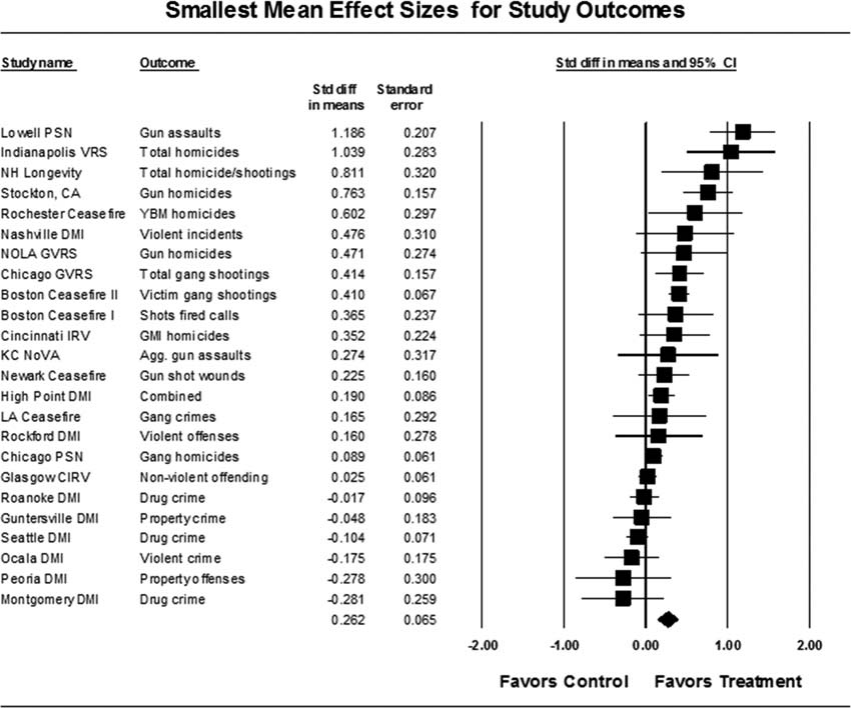
Smallest mean effect sizes for study outcomes

A majority of the studies reported effect sizes that favored treatment conditions over control conditions (91.7%, 22 of 24), with the Ocala and Montgomery County programs reporting nonsignificant and very small negative sizes. As described earlier, we conducted additional meta‐analyses of the largest and smallest effect sizes reported for each study.^13^ For the largest effect size meta‐analysis (Figure 2), the overall standardized mean difference effect size was medium (0.577, *p *< .05; 95% CI = 0.427, 0.726). For the smallest effect size meta‐analysis (Figure 3), the overall standardized mean difference effect size was modest (0.262, *p* < .05; 95% CI = 0.135, 0.389).

#### 6.4.2 Program type and research design as effect size moderators

**Figure 4:**
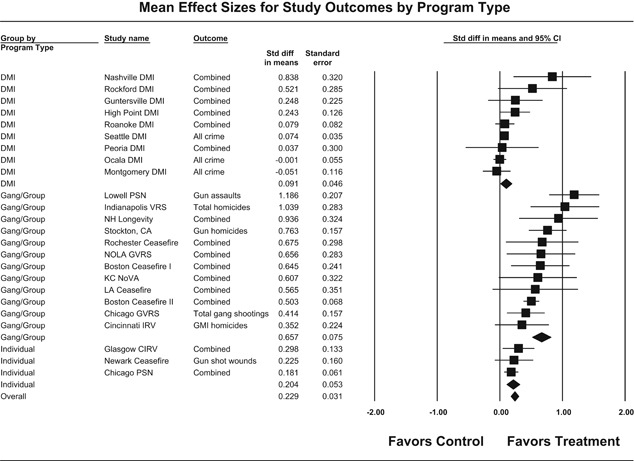
Mean effect sizes for study outcomes by program type

Focused deterrence strategies have been directed at reducing crime by street gangs and criminally active groups, overt drug markets, and high‐risk individuals. These programs represent differing applications of focused deterrence strategies to control distinct types of problems. The inclusion of moderator variables, such as program and research design types, help to explain and understand differences across studies in the outcomes observed (Lipsey, 2003). Figure 4 presents a random effects model examining the mean effect sizes for the three different program types. It is important to note that the *Q*‐statistic associated with the between‐group variation was large and statistically significant (*Q* = 41.555, *df* = 2, *p* < .05), suggesting that program type was influential in determining effect sizes. The gang/group intervention programs were associated with the largest within‐group effect size (0.657, *p *< .05), followed by the high‐risk individuals programs (0.204, *p *< .05) and the DMI programs (0.091, *p *< .05). When program type was included as a moderator, the meta‐analysis estimated a more modest overall effect size (0.229, *p *< .05).

The smaller mean effect size associated with the DMI programs was influenced by the noteworthy share of programs with reported threats to the integrity of the focused deterrence treatment. Not surprisingly, DMI programs that were implemented with higher treatment fidelity generated larger overall crime reduction impacts. As mentioned earlier, four of the nine (44.4%) eligible DMI programs suffered from implementation difficulties centered on securing the necessary community involvement in targeted drug market areas (Guntersville, Montgomery County, Peoria, and Roanoke). When treatment integrity was included as an effect size moderator for the nine DMI studies, programs with noted implementation issues had a smaller nonstatistically significant mean effect size (0.053). In contrast, the mean effect size for DMI programs without implementation difficulties suggested a modest, statistically significant crime reduction impact (0.184, *p* < .05).

The meta‐analysis in the previous iteration of the Campbell systematic review estimated a larger overall mean effect size (0.604) relative to the current meta‐analysis (0.383). This difference is primarily due to the greater prevalence of more rigorous quasi‐experimental designs with higher levels of internal validity among the studies included in the current systematic review. It is well known among social scientists that program evaluations with more rigorous research designs tend to report null effects. As Peter H. Rossi's Iron Law of Evaluation states, “The expected value of any net impact assessment of any large scale social program is zero” (Rossi, 1987, p. 3). And as his Stainless Steel Law of Evaluation posits, “The better designed the impact assessment of a social program, the more likely is the resulting estimate of net impact to be zero” (Rossi, 1987, p. 3). Given the important distinction in methodological quality between the nonequivalent and matched quasi‐experimental studies, we examined research design as a moderator variable.

**Figure 5:**
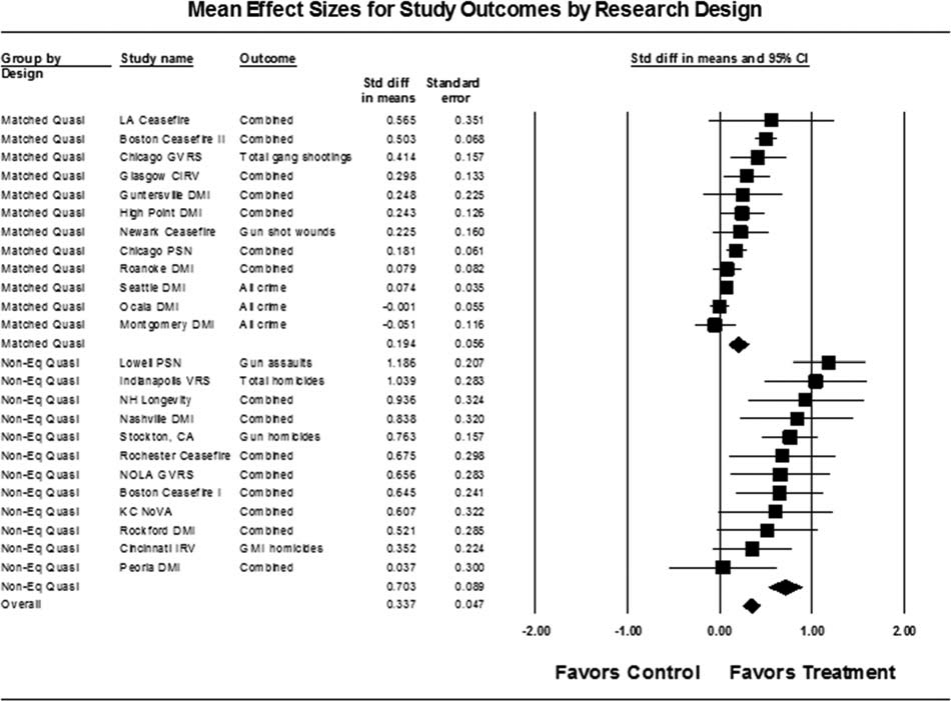
Mean effect sizes for study outcomes by research design

Figure 5 presents a random effects model examining the two different classes of quasi‐experimental designs included in this review. It is important to note that the *Q*‐statistic associated with the between‐group variation was large and statistically significant (*Q* = 23.349, *df* = 1, *p *< .05), suggesting that research design was influential in determining effect sizes. In this analysis, the nonequivalent quasi‐experimental designs were associated with a much larger within‐group effect size (0.703, *p *< .05) relative to the matched quasi‐experimental designs (0.194, *p* < .05). When research design type was included as a moderator, the meta‐analysis estimated a more modest overall effect size (0.337, *p* < .05).^14^ While the biases in quasi‐experimental research are not clear (e.g., Campbell & Boruch, 1975; Wilkinson & Task Force on Statistical Inference, 1999), recent reviews in crime and justice suggest that weaker research designs often lead to more positive outcomes (Weisburd, Lum, & Petrosino, 2001; Welsh, Peel, Farrington, Elffers, & Braga, 2011).

#### 6.4.3 Publication bias

Publication bias, generally defined as the concern that the collection of studies easily available to a reviewer represents those studies most likely to have statistically significant results, presents a strong challenge to any review of evaluation studies (Rothstein, 2008). The credibility of a review arguably depends more heavily on the collection of studies reviewed than on which statistical methods of synthesis are used (Wilson, 2009). Similar to the problem of a biased study sample leading to biased results in an individual study, a biased collection of studies will potentially lead to biased conclusions in a systematic review (Rothstein & Hopewell, 2009). As reported earlier, our search strategies were designed to mitigate the potential effects of publication bias on our analyses. Indeed, it is encouraging that more than one‐third of the eligible studies (9 of 24, 37.5%) were acquired through grey literature sources such as published reports, theses, dissertations, unpublished reports, and unpublished working papers. The studies identified through grey literature sources reported a much smaller overall mean effect size (0.156, *p *< .05) when compared to the overall mean effect size (0.475, *p* < .05) reported by studies in published journal articles, suggesting that our search strategies were successful in identifying a range of focused deterrence studies with varying effects on crime outcomes.^15^

Like many systematic reviews, our meta‐analyses used the trim‐and‐fill procedure to explore whether publication bias might be affecting the results and to estimate how the reported effects would change if the bias were to be removed (Duval, 2005; Duval & Tweedie, 2000). The diagnostic funnel plot is based on the idea that, in the absence of bias, the plot of study effect sizes should be symmetric about the mean effect size. If there is asymmetry, the trim‐and‐fill procedure imputes the missing studies, adds them to the analysis, and then recomputes the mean effect size. Trim‐and‐fill procedures do suffer from some well‐known limitations that could result in the underestimation or overestimation of publication bias (Rothstein, 2008; Simonsohn, Nelson, & Simmons, 2014).^16^ Nonetheless, this approach does provide reviewers with a well‐understood measure of the possible influence of bias on their meta‐analytic results.

**Figure 6:**
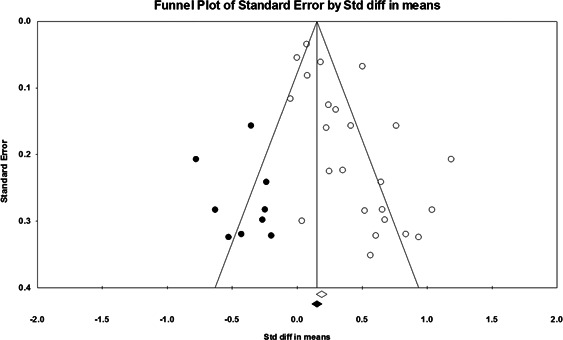
Funnel plot of standard error by standardized difference in means. Note: Empty circles are the original studies. Filled‐in circles indicate 9 imputed studies from the trim‐and‐fill analysis

A visual inspection of the resulting funnel plot indicated some asymmetry with more studies with a large effect and a large standard error to the right of the mean than the left of the mean. The trim‐and‐fill procedure determined that nine studies should be added to create symmetry. The funnel plot with imputed studies is presented in Figure 6. These additional studies modestly altered the mean effect size estimate. The mean random effect decreased from 0.383 (95% CI [0.264, 0.503]) to 0.215 (95% CI [0.098, 0.332]). Indeed, the 95% confidence intervals substantially overlap, suggesting that the underlying parameters may not be different. Nevertheless, the trim‐and‐fill result suggests mild publication selection bias. However, the adjusted mean effect size remained a similar statistically significant small size and, as such, the observed publication bias does not appear to be sufficient to nullify the results (as suggested by the funnel plot in Figure 6).

## 7 DISCUSSION

### 7.1 Summary of main results

The results of our review suggest focused deterrence strategies may generate noteworthy crime control impacts. In 19 of the 24 eligible studies, evaluators reported that the implementation of the evaluated program was associated with a statistically significant crime reduction effect on a targeted crime problem. The results of our meta‐analysis of effect sizes estimated a statistically significant, moderate overall mean effect in favor of focused deterrence strategies. When these second‐order effects were measured, focused deterrence programs did not result in significant crime displacement impacts. Rather, focused deterrence programs tended to generate diffusion of crime control benefits that extended into proximate areas and socially connected groups that did not receive direct treatments.

The magnitude of the impact of focused deterrence varied by program type. The strongest crime reduction impacts were associated with focused deterrence programs designed to reduce serious violence generated by ongoing conflicts among gangs and criminally active groups. Even when the integrity of the treatment applied was considered, DMI programs generated the smallest crime reduction impacts associated with the three different kinds of focused deterrence strategies.

### 7.2 Overall completeness and applicability of evidence

The promising findings of this review have widespread applicability to the field of policing and crime prevention. The previous iteration of the review contained 10 studies dating back to 2001. This updated review identified 14 new eligible studies released between 2011 and 2015 for a new total of 24 eligible studies. With the addition of a number of new focused deterrence studies, the essential finding of this review was reaffirmed: focused deterrence programs seem to generate modest reductions in crime (Braga & Weisburd, 2012, 2011). Nearly all of the eligible focused deterrence interventions occurred in the United States (23 studies). Only one study involved an evaluation of an intervention implemented in another country (Scotland). As such, further research is needed to determine general applicability of focused deterrence across varying international contexts.

### 7.3 Quality of the evidence

There was some evidence that the research design used in the included studies moderated the magnitude of the impact of focused deterrence on crime. The within‐group effect size for weaker nonequivalent quasi‐experimental designs was larger when compared to more rigorous matched quasi‐experimental designs. Nevertheless, the effects of focused deterrence on crime remained statistically significant regardless of the research design. No randomized experiments testing the efficacy of focused deterrence on crime were completed during the implementation of our search strategies.

### 7.4 Limitations and potential biases in the review process

Outcome measured by studies included in this review relied exclusively on official records and did not include alternative measures such as self‐reported victimization. While more than one‐third of the eligible studies came from grey literature sources, our trim‐and‐fill procedures suggests the possibility of modest publication bias. Nevertheless, the possible publication bias suggested by the trim‐and‐fill analysis was not enough to nullify our overall findings that focused deterrence programs generate modest crime control impacts.

### 7.5 Agreements and disagreements with other studies or reviews

The results of this systematic review support the conclusion of the National Academies' Committee on Proactive Policing report that focused deterrence can be effective in preventing crime (Weisburd & Majmundar, 2018). As described at the outset of this report, there have been scholarly disagreements over the crime control efficacy of focused deterrence. This is particularly true of varying assessments of the well‐known Boston Ceasefire strategy implemented during the 1990s. The results of this review are congruent with the perspectives of scholars who supported the violence reduction value of Boston Ceasefire (e.g., Cook & Ludwig, 2006; Morgan & Winship, 2007) and diverge with the perspective of the skeptics (see, e.g., Fagan, 2002; Rosenfeld et al., 2005).

## AUTHORS' CONCLUSIONS

### Implications for practice and policy

The positive findings of our systematic review and meta‐analysis, in combination with the strong theoretical literature supporting the mechanisms of focused deterrence, provide solid support for the adoption of such programs by cities suffering from serious crime problems. Indeed, it is unusual that an overwhelming majority of program effect sizes included in our meta‐analysis favored treatment over control conditions. It is also noteworthy that the strongest crime reduction impacts were associated with focused deterrence programs designed to reduce serious violence generated by ongoing conflicts among gangs and criminally active groups. Similarly, the National Academies' Committee on Proactive Policing concluded that “evaluations of focused deterrence programs show consistent crime control impacts in reducing gang violence” (Weisburd & Majmundar, 2018, p. 175). These encouraging findings suggest that focused deterrence might be particularly effective at controlling violence emanating from recurring group‐based conflicts relative to crime problems driven by disorderly street drug markets or individual repeat offenders.

DMI programs were associated with the smallest crime reduction impacts of the three focused deterrence program types. Given the large body of research that has shown the ineffectiveness of many police crime prevention efforts (Visher & Weisburd, 1998), the overall crime reduction impact generated by DMI programs is still noteworthy. However, the smaller crime control benefits suggested by our meta‐analysis are different from effectiveness claims made from early applications of the approach. For instance, reflecting on several short term, simple pretest versus posttest one‐group‐only comparisons of violent crime incidents in treated areas, Kennedy and Wong (2009, p. 43) suggest “that it may be possible to close overt community drug markets and substantially reduce violent and drug‐related crime.” In their more rigorous quasi‐experimental evaluation of the High Point DMI, Corsaro et al. (2012) suggest a more modest 14% reduction in violent crime incidents associated with the approach.

It is interesting to note that these findings follow those that have been generated in studies of developmental prevention. Summarizing systematic reviews in this area, Farrington, Ttofi, and Lösel (2016) find that programs that focus on higher risk youth are more likely to be successful. In correctional evaluations, the importance of focusing on high‐risk offenders has also been a key element for predicting program success (e.g., Andrews & Bonta, 2006). Our finding that the largest impacts are found for programs that focus on the most violent offenders is congruent with what has been observed in treatment programs more generally. This insight could further explain the stronger impacts of focused deterrence on the violent behavior of high‐risk groups and repeat offenders relative to the smaller impacts when implemented to control the behaviors of a broader range of offenders participating in street‐level drug sales.

The existing empirical evidence suggests that “person focused” policing interventions associated with the standard model of policing, such as programs designed to arrest and prosecute repeat offenders, were not effective in controlling crime (National Research Council, 2005). In contrast, the evaluation evidence reviewed here suggests that focused deterrence strategies, designed to change offender behavior through a blended enforcement, social service and opportunity provision, and community‐based action approach, is effective in controlling crime. Other key programmatic elements include strategic analyses of targeted crime problems and a well‐developed communications plan designed to make targeted offenders understand the new regime that is being imposed on them.

The available literature further suggests that focused deterrence strategies, especially DMI programs, may be difficult to implement and these challenges can undermine its crime control efficacy in certain jurisdictions. It is important to recognize that successful focused deterrence programs follow a deliberate strategy development process rather than the simple adoption of tactics applied in other jurisdictions. Consistent with its problem‐oriented policing roots, the focused deterrence framework requires local jurisdictions to conduct careful upfront research on the nature of targeted crime problems to customize a response to identified underlying conditions and dynamics that fits both local community contexts and the operational capacities of criminal justice, social service, and community‐based agencies. The successful implementation of focused deterrence strategies requires the establishment of a “network of capacity” consisting of dense and productive relationships among these diverse partnering agencies (see Braga & Winship, 2006). Cities without robust networks in place have found it difficult to implement and sustain focused deterrence strategies.

### Implications for research

More than half of the eligible studies included in this updated systematic review were completed after the original Campbell review (Braga & Weisburd, 2012, 2011). Unfortunately, none of the newly identified studies responded to the original review's call for the next generation of focused deterrence program evaluations to shed some much‐needed light on the theoretical mechanisms underlying focused deterrence policing. Like many evaluations of crime prevention programs, nearly all of the focused deterrence program evaluations included in this review could be described as “black box” evaluations where it is uncertain which program elements were most important in generating observed crime reduction effects.

While there is a strong logic model for predicting positive outcomes in focused deterrence programs, we have little knowledge of which of the mechanisms underlying that model have the strongest impacts on outcomes. Deterrence certainly remains a key element to understanding why focused deterrence policing works. Nonetheless, it seems particularly important to assess how elements of procedural justice and collective efficacy influence program outcomes. In recent years there has been growing concern not just about whether policing impacts on crime but also upon how it affects communities. The President's Task Force on 21st Century Policing (2015), for example, identified public trust in the police to be the “first pillar” of policing. While we do not have robust evidence on the mechanisms underlying prevention in most focused deterrence evaluations, the Chicago PSN quasi‐experiment provides encouraging evidence for prevention mechanisms that would enhance public evaluations of legitimacy (Papachristos et al., 2007). The Chicago PSN evaluation suggests that direct communications with offenders in a procedurally just manner in the context of maintaining an enforcement environment enhances program effectiveness. This suggests potential for focused deterrence policing to be implemented in ways that are likely to increase legitimacy among offenders. We need more studies that examine this and other potential mechanisms that may improve community outcomes.

None of the new studies used rigorous randomized controlled trial designs to evaluate the crime reduction impacts of focused deterrence programs. This continues to be a key weakness in drawing conclusions about focused deterrence programs. However, the updated review reveals that the quality of quasi‐experimental evaluations of focused deterrence strategies have improved greatly over time. Contemporary quasi‐experimental evaluations of focused deterrence strategies tend to use sophisticated statistical matching techniques, panel designs, and higher‐powered statistical models. Future evaluations of focused deterrence programs targeting repeat offenders and drug markets could be further strengthened by using randomized experimental designs. Well after the completion of the search strategies in this review, Hamilton, Rosenfeld, and Levin (2018) completed the first randomized controlled trial of an individual offender focused deterrence program centered on high‐risk probationers and parolees. Their study found that subjects who attended the focused deterrence notification meeting were less likely than those who did not receive treatment to be arrested over the following 17 months. It would be considerably more complicated to use randomized experimental designs to evaluate gang and criminally active group focused deterrence programs given that these interventions intentionally seek to generate spillover effects that could contaminate control gangs, groups, and areas. Braga and Weisburd (2014) suggest that multisite cluster randomized trial designs could be used to conduct more rigorous evaluations of gang and group violence reduction strategies.^17^

Comparative research on applications of focused deterrence strategies in other countries is also needed to determine whether these violence reduction policies and practices can be transferred to settings outside U.S. urban environments. Experiences in Glasgow, Scotland, suggest that the approach may be beneficial in addressing serious youth violence problems in other Western countries (Deuchar, 2013). However, implementation in more challenging global environments, such as Turkey and Brazil (National Network for Safe Communities, 2013), represent strong tests for the focused deterrence approach. Many questions need to be answered. For instance, is it possible to develop a network of capacity that could mobilize communities to complement law enforcement efforts to control the violent behaviors of drug gangs in severely disadvantaged favelas of Rio de Janeiro? Drawing on the positive experiences in developing such capacities in very violent disadvantaged neighborhoods in the United States, it seems possible. Obviously, it would be highly problematic for corrupt, violent, and incompetent police forces to lead the implementation of focused deterrence strategies. Barring that concerning possibility, the flexible problem‐solving framework undergirding focused deterrence strategies suggests that the approach can be appropriately tailored to varying urban contexts. At this point in time, the potential violence reduction efficacy of these approaches in other countries is largely based on speculation rather than empirical facts and practical experience. However, experimentation with focused deterrence strategies to control crime problems beyond U.S. settings is clearly warranted by the available scientific evidence.

While the evaluation evidence needs to be strengthened with additional rigorous randomized experimental field trials, and more developed study of the theoretical mechanisms underlying its impacts, our review suggests that jurisdictions suffering from gang and group‐related violence problems should add focused deterrence strategies to their existing portfolio of prevention and control interventions. The effects of focused deterrence on crime problems generated by repeat offenders and street‐level drug markets seem to be smaller and, as such, more caution should be applied when implementing these kinds of programs. Jurisdictions looking to implement focused deterrence programs need guidance on the key operational elements of these varied approaches. As evaluation evidence and practical experience continues to accumulate, a premium must be placed on identifying these complementary crime control mechanisms and isolating their impacts on targeted crime problems.

## ROLES AND RESPONSIBILITIES

Braga and Weisburd designed the original systematic review following established Campbell Collaboration conventions and protocols; Braga, Weisburd, and Turchan designed the second iteration. With the assistance of Phyllis Schultze, Turchan and Braga executed the varied search strategies to identify eligible studies.

- Content: Anthony Braga, David Weisburd, Brandon Turchan
- Systematic review methods: Anthony Braga, David Weisburd, Brandon Turchan
- Statistical analysis: Anthony Braga, David Weisburd, Brandon Turchan
- Information retrieval: Anthony Braga, Brandon Turchan

## SOURCES OF SUPPORT

The research for the original version of this review was supported through funds provided by the National Policing Improvement Agency, United Kingdom. The authors did not receive any financial or other support for the completion of this updated review.

## DECLARATIONS OF INTEREST

Weisburd and Turchan have no vested interest in the outcomes of this review, nor any incentive to represent findings in a biased manner. With colleagues, Professor Braga has been an evaluator of four pulling levers focused deterrence programs: Operation Ceasefire in Boston I and II, Project Safe Neighborhoods in Lowell, and Operation Peacekeeper in Stockton. He has also written several book chapters that provide narrative reviews of pulling levers strategies and suggest these interventions generate crime control impacts. The prior narrative reviews provide the basis for Professor Braga's interest in carrying out this systematic review. Professor Braga would not have been uncomfortable if the findings had shown that prior narrative reviews were incorrect.

## PLANS FOR UPDATING THE REVIEW

In accordance with Campbell Collaboration guidelines, Braga, Weisburd, and Turchan will update this review once every 5 years.

## Footnotes

1 For a complete list of cities supported by the National Network for Safe Communities, go to https://nnscommunities.org/impact/cities (last accessed May 24, 2019).

2 The previous iteration of this systematic review did not include studies with one‐group‐only interrupted time‐series design designs. As will be shown below, the updated review identified only one such study (Delaney, 2006).

3 These journals were: *Criminology, Criminology & Public Policy, Justice Quarterly, Journal of Research in Crime and Delinquency, Journal of Criminal Justice, Police Quarterly, Policing, Police Practice and Research, British Journal of Criminology, Journal of Quantitative Criminology, Crime & Delinquency, Journal of Criminal Law and Criminology,* and *Policing and Society.* Hand searches covered 1979–2015.

4 Ms. Phyllis Schultze of the Gottfredson Library at the Rutgers University School of Criminal Justice assisted with the initial abstract search and was consulted throughout on our search strategies.

5 Maintained by the Gottfredson Library at the Rutgers University School of Criminal Justice.

6 The now‐defunct Campbell Collaboration Social, Psychological, Educational and Criminological Trials Register was consulted for the original version of this systematic review.

7
https://www.campbellcollaboration.org/effect‐size‐calculato.html.

8
https://methods.cochrane.org/sites/methods.cochrane.org.bias/files/public/uploads/EPOC%20Data%20Collection%20Checklist.pdf.

9 The grey literature is a term applied to sources of information that are not commercially published and is typically composed of technical reports, working papers, government and agency reports, and conference proceedings. Wilson (2009) has argued that there is often little difference in methodological quality between published and unpublished studies, suggesting the importance of searching the grey literature.

10 During the development of this report, the New Haven study was accepted for publication at *Crime & Delinquency* and the Roanoke study was accepted for publication at *Journal of the American Statistical Association.*

11 Table 2 summarizes the characteristics of the key focused deterrence evaluation identified through the varied search processes. Five evaluations had companion quasi‐experimental analyses that supported the program impact conclusions presented here: Boston Ceasefire I (Piehl et al., 2003), Boston Ceasefire II (Braga, Apel, & Welsh, 2013), Chicago PSN (Wallace, Papachristos, Meares, & Fagan, 2016), High Point DMI (Corsaro, 2013), and Indianapolis (Corsaro & McGarrell, 2009). In addition to the Corsaro et al. (2012) evaluation, the RAND Corporation completed an independent evaluation of the High Point DMI using a synthetic control quasi‐experimental design (Saunders et al., 2015). The RAND evaluation found a slightly stronger impact of the DMI program on targeted outcomes. They find that in the year following a DMI, calls for service decreased 16% and violent crimes decreased 34%, on average, compared to synthetic control markets. The RAND evaluation also found no evidence of statistically significant crime displacement or diffusion effects after a DMI was implemented.

12 The National Network for Safe Communities raised concerns to the RAND Corporation over the treatment fidelity of the DMI programs that were sponsored by the U.S. Bureau of Justice Assistance and implemented under the guidance of a technical assistance team from Michigan State University. One key concern centered on the absence of reconciliation efforts between police and affected communities on perceived harms associated with prior drug control tactics. Reconciliation is viewed as a critical component of developing the necessary community support needed to exert informal social control over drug sellers in targeted overt drug markets. Further concerns involved unclear definitions of the drug market areas to be targeted for intervention, a lack of opportunity and resources available to targeted dealers with banked cases, and other implementation issues. Personal communication with David Kennedy on February 25, 2017; Memorandum on “DMI Integrity” from David Kennedy to Beau Kilmer and Mark Kleiman, November 17, 2015.

13 Random effects models were used to estimate the overall standardized mean effect sizes. For the largest effect size meta‐analysis, *Q* = 152.740, *df* = 23, *p* < .05, Tau^2^ = 0.093. For the smallest effect size meta‐analysis, *Q* = 109.537, *df* = 23, *p* < .05, Tau^2^ = 0.062.

14 These findings are similar to the previous iteration of the Campbell focused deterrence systematic review. In the prior meta‐analysis, the nonequivalent quasi‐experimental designs had an effect size of 0.766 (*p* < .05), the matched quasi‐experimental designs had an effect size of 0.196 (*p* < .05) and, when research design type was included as a moderator, the overall effect size was 0.312 (*p* < .05). However, as noted earlier, the current review has a larger share of matched quasi‐experimental designs relative to nonequivalent quasi‐experimental designs. As such, the overall mean effect size estimated in the current meta‐analysis is smaller (0.383) as compared to the original review (0.604).

15 For grey literature studies, *Q* = 23.204, *df* = 8, *p* < .05. For journal article studies, *Q* = 46.913, *df* = 14, *p* < .05. The between *Q* = 10.079, *df* = 1, *p* < .05, suggesting that the publication type produced statistically significant differences in observed crime outcomes. The moderated overall effect size was 0.296 (*p *< .05).

16 As discussed by Rothstein (2008, p. 69), the trim‐and‐fill procedure is based on the notion that, in the absence of bias, a funnel plot of study effect sizes will be symmetric about the mean effect. If there are more small studies on one side than on the other side of the bottom of the funnel plot, there is concern that some studies may have been censored from the meta‐analysis. The trim‐and‐fill approach imputes the missing studies, adds them to the analysis, and then recomputes the mean effect size. The most notable limitation is that this approach assumes the observed asymmetry is a result of publication bias rather than of true differences in the results of the small studies compared with the larger ones.

17 Cluster randomized experiments represent a variation of the classic randomized controlled trial design in which clusters (groups) of subjects, rather than individual subjects, are randomly allocated to treatment and control conditions. This design allows better control of treatment “contamination” across individual subjects. In the case of gang violence, this contamination is the stable unit treatment valuation assumption (SUTVA) problem generated by social connections among gangs. In a multisite cluster randomized trial, clusters of subjects are randomly allocated to treatment and control conditions in two or more sites. Randomly allocating distinct clusters of gangs connected by rivalries and alliances to treatment and control conditions limits the treatment contamination problem. Researchers in each participating city would need to identify gang conflict and alliance networks and apply social network analysis techniques to specify distinct socially connected cliques of gangs. Researchers would also need to track shootings by specific gangs during preintervention and postintervention time periods in participating cities.

## APPENDIX A LIST OF 100 EXPERTS CONTACTED DURING SEARCH PROCESS

1. Allan Abrahamse, RAND Corporation
2. Robert Apel, Rutgers University
3. David Bayley, University at Albany—SUNY
4. Lawrence Bobo, Harvard University
5. Brenda Bond, Suffolk University
6. Robert Boruch, University of Pennsylvania
7. Douglas Boyle, Rutgers University
8. Alfred Blumstein, Carnegie Mellon University
9. Rod Brunson, Rutgers University
10. Chia‐Cherng Cheng, Rutgers University
11. Steven Chermak, Michigan State University
12. Ronald V. Clarke, Rutgers University
13. Philip J. Cook, Duke University
14. Suzanne J. Cooper, Harvard University
15. Nicholas Corsaro, University of Cincinnati
16. Linda Cottler, University of Florida
17. Shea Cronin, Boston University
18. Robert Crutchfield, University of Washington
19. Dorothy Currie, University of St. Andrews
20. Scott Decker, Arizona State University
21. Peter D. Donnelly, University of St. Andrews
22. John E. Eck, University of Cincinnati
23. Robin Engel, University of Cincinnati
24. Jeffrey Fagan, Columbia University
25. Graham Farrell, Loughborough University
26. Andrew M. Fox, University of Missouri—Kansas City
27. Herman Goldstein, University of Wisconsin
28. Peter Grabosky, Australian National University
29. Clifford Grammich, RAND Corporation
30. Peter Greenwood, Greenwood and Associates
31. David Hemenway, Harvard University
32. Natalie K. Hipple, Indiana University
33. Joel Horowitz, Northwestern University
34. David M. Hureau, Harvard University
35. Robert L. Johnson, Rutgers—New Jersey Medical School
36. Shane Johnson, University College London
37. David M. Kennedy, John Jay College of Criminal Justice
38. David S. Kirk, University of Texas at Austin
39. Mark A.R. Kleiman, University of California—Los Angeles
40. David A. Klinger, University of Missouri—St. Louis
41. John Klofas, Rochester Institute of Technology
42. Johannes Knutsson, Norwegian Police University College
43. Jennifer Lanterman, University of Nevada—Reno
44. Janet Lauritsen, University of Missouri—St. Louis
45. Gloria Laycock, University College London
46. Steven Levitt, University of Chicago
47. Will Linden, Violence Reduction Unit—Glasgow
48. Jens Ludwig, University of Chicago
49. Cynthia Lum, George Mason University
50. Russell Lundberg, Sam Houston State University
51. Tracey Maclin, Boston University
52. Edward R. Maguire, American University
53. Stephen D. Mastrofski, George Mason University
54. Lorraine Mazerolle, University of Queensland
55. Jack McDevitt, Northeastern University
56. Edmund McGarrell, Michigan State University
57. Tracey Meares, Yale University
58. Jeremy Miles, RAND Corporation
59. Terrie Moffitt, Duke University
60. Mark H. Moore, Harvard University
61. Susan Murphy, University of Michigan
62. Daniel Nagin, Carnegie Mellon University
63. Karen Norberg, Washington University of St. Louis
64. Kenneth J. Novak, University of Missouri—Kansas City
65. Andrew Papachristos, Yale University
66. Joseph Pascarella, St. Joseph's College—SUNY
67. John V. Pepper, University of Virginia
68. Ruth Peterson, Ohio State University
69. Anne M. Piehl, Rutgers university
70. Glenn L. Pierce, Northeastern University
71. Alex Piquero, University of Texas at Dallas
72. Peter Reuter, University of Maryland
73. Greg Ridgeway, University of Pennsylvania
74. K. Jack Riley, RAND Corporation
75. Dennis Rosenbaum, University of Illinois—Chicago
76. Richard Rosenfeld, university of Missouri—St. Louis
77. Jessica Saunders, RAND Corporation
78. Elaine B. Sharp, University of Kansas
79. Lawrence Sherman, University of Cambridge
80. Wesley Skogan, Northwestern University
81. Nick Tilley, University College London
82. Marie S. Tillyer, Unversity of Texas—San Antonio
83. George E. Tita, University of California—Irvine
84. Jeremy Travis, John Jay College of Criminal Justice
85. Tom Tyler, Yale University
86. Stewart Wakeling, California Partnership for Safe Communities
87. Joel Waldfogel, University of Minnesota
88. Samuel Walker, University of Nebraska—Omaha
89. Danielle Wallace, Arizona State University
90. Elin J. Waring, Lehman College—CUNY
91. Alexander Weiss, Alexander Weiss Consulting
92. Charles Wellford, University of Maryland
93. Brandon C. Welsh, Northeastern University
94. Damien J. Williams, University of St. Andrews
95. Jeremy M. Wilson, Michigan State University
96. Christopher Winship, Harvard University
97. Garen Wintemute, University of California—Davis
98. Robert Worden, University at Albany—SUNY
99. Majid B. Yaghoub, University of Missouri—Kansas City
100. Franklin Zimring, University of California—Berkeley

## APPENDIX B CODING SHEETS

ELIGIBILITY CHECK SHEET

1. Document ID: __ __ __ __
2. Study Author Name(s): ___________________________________________
3. Study Title:__________________________________________________
4. Journal Name, Volume and Issue: ___________________________________
5. Document ID: __ __ __ __
6. Coder's Initials __ __ __
7. Date eligibility determined: ____________
8. A study must meet the following criteria in order to be eligible. Answer each question with a “yes” or a “no.”

a. The study is an evaluation of a pulling levers focused deterrence intervention._____
b. The study includes a comparison group (or a preintervention comparison period in the case of pre‐post studies), which did not receive the treatment condition (focused deterrence). Studies may be experimental or quasi‐experimental. ______
c. The study reports on at least one crime outcome. ______
d. The study is written in English. _____

If the study does not meet the criteria above, answer the following question:

a. The study is a review article that is relevant to this project (e.g., may have references to other studies that are useful, may have pertinent background information) ____

9. Eligibility status:

____ Eligible
____ Not eligible
____ Relevant review

Notes:

________________________________________________________________________________________________________________________________

CODING PROTOCOL

Reference Information

1. Document ID: __ __ __ __
2. Study author(s): _________________________________________________
3. Study title: _____________________________________________________
4. Publication type: ______

1. Book
2. Book chapter
3. Journal article (peer‐reviewed)
4. Thesis or doctoral dissertation
5. Government report (state/local)
6. Government report (federal)
7. Police department report
8. Technical report
9. Conference paper
10. Other (specify))_____________________

5. Publication date (year): ______________

6. Journal Name: ____________________________________________
7. Journal Volume: _______________
8. Journal Issue: ____________

7. Date range of research (when research was conducted):

Start: ____________
Finish: ____________

8. Source of funding for study: ____________________________________

9. Country of publication: ___________________

10. Date coded: ___________

11. Coder's Initials: __ __ __

### Describing the Pulling Levers Focused Deterrence Intervention

12. Did the study formally identify the treatment as a pulling levers policing intervention?

Yes
No

12. If No, what did the study call the intervention? ________________________________________________________________

13. What crime problem was targeted for the intervention? (Select all that apply)

1. Total homicide
2. Youth homicide
3. Gun violence
4. Drug‐related violence
5. Street‐level drug markets
6. Other (specify) _____________

14. Who were the primary targets of the intervention? (Select all that apply)

1. Street gangs
2. Semiorganized/organized crime
3. Informal criminally active groups
4. Drug‐selling crews
5. High‐risk individuals
6. Other group (specify) ___________

15. If the intervention was primarily targeted at “high‐risk individuals,” please describe the individuals: (Select all that apply)

1. Probationers
2. Parolees
3. Convicted felons
4. Gang members
5. Street‐level drug dealers
6. Other (specify) _____________
7. N/A

16. Specifically, what event(s) makes up the problem?

________________________________________________________________

________________________________________________________________

17. Was the intervention developed based on an analysis of the targeted problem?

1. No, the intervention was implemented without any analysis of the targeted problem.
2. Yes, the intervention was implemented after a cursory/limited analysis of the targeted problem.
3. Yes, the intervention was implemented after a thorough analysis of the targeted problem.
4. Other (specify) _____________

18. At what unit of analysis was the treatment delivered/intervention directed at? (Select all that apply)

1. Specific individuals
2. Groups of individuals
3. Microplaces (crime hot spots, specific housing project, etc.)
4. Small police‐defined units (such as beats)
5. Larger police‐defined units (such as districts or sectors)
6. Neighborhood or community level
7. City or town level
8. State level
9. Other (specify) _____________

19. What agency was primarily responsible for the implementation of the intervention? (Select the lead agency only)

1. Local police
2. State police
3. Federal law enforcement agencies (e.g., ATF, DEA, FBI)
4. Local/county/state prosecutor
5. Federal prosecutor
6. Probation
7. Parole
8. Correctional agency
9. Local/county/state governmental agency (e.g., Mayor's Criminal Justice Office)
10. Social service provider
11. Community‐based agency
12. Other (specify) ___________

20. What groups were involved in the implementation of the intervention? (Select all that apply)

1. Local police
2. State police
3. Federal law enforcement agencies (e.g. ATF, DEA, FBI)
4. Local/county/state prosecutor
5. Federal prosecutor
6. Probation
7. Parole
8. Correctional agency
9. Local/county/state governmental agency (e.g., Mayor's Criminal Justice Office)
10. Social service provider
11. Community‐based agency
12. Other (specify) _____________

21. What key elements of the focused deterrence strategy were identified in the program evaluation? (Select all that apply)

1. Clear “triggering” event that provoke the pulling levers response
2. Enforcement levers that could be customized to targeted groups/individuals
3. Social services/opportunities for targeted groups/individuals
4. Communications strategy
5. Other (specify) _____________

22. If a communications strategy was present, please identify the key elements of the message(s) (Select all that apply)

1. Deterrence message
2. Social service/opportunity‐based message
3. Changing norms/decision making message
4. Reintegration of offender(s) back into community message
5. Other (specify) _____________
6. N/A

23. If a communications strategy was present, how were the message(s) delivered marketed to the targeted audience? (Select all that apply)

1. Formal meetings (e.g., forums or “call‐ins”)
2. Home visits
3. On the street (i.e., “retail” delivery on corners, in parks, etc.)
4. Advertising (e.g., billboards, TV/radio spots, handouts, etc.)
5. School assemblies
6. Correctional setting (e.g., in‐prison meeting, in‐juvenile detention facility, etc.)
7. Other (specify) _____________
8. N/A

24. What did the evaluation indicate about the implementation of the response? _

1. The response was implemented as planned or nearly so
2. The response was not implemented or implemented in a radically different way than originally planned
3. Unclear/no process evaluation included

25. If the process evaluation indicated there were problems with implementation of the response, describe these problems:

________________________________________________________________

________________________________________________________________

________________________________________________________________

***__________________________________________________________***

26. If the process evalunation identified inadequate participation by involved agencies, indicate the agencies below that were responsible for weak participation (Select all that apply)

1. Local police
2. State police
3. Federal law enforcement agencies (e.g., ATF, DEA, FBI)
4. Local/County/State prosecutor
5. Federal prosecutor
6. Probation
7. Parole
8. Correctional agency
9. Local/County/State governmental agency (e.g., Mayor's Criminal Justice Office)
10. Social service provider
11. Community‐based agency
12. Other (specify) _____________
13. N/A

27. Country where study was conducted: _____________________________

28. City (and state/province, if applicable) where study was conducted: _________

**Methodology/Research design:**

29. Type of study: _________

1. Randomized experiment
2. Nonequivalent control group (quasi‐experimental)
3. Matched control group (quasi‐experimental)
4. Other (specify) __________________

30. How were study units allocated to treatment or comparison conditions?

1. Simple random allocation
2. Random allocation in pairs, blocks, or some other sophisticated technique
3. Simple descriptive matching
4. Sophisticated statistical matching (e.g., propensity scores)
5. Other (specify) ___________________

31. Explain how independent and extraneous variables were controlled so that it was possible to disentangle the impact of the intervention or how threats to internal validity were ruled out.

________________________________________________________________

________________________________________________________________

________________________________________________________________

*The following questions refer to the units receiving treatment:*

32. Units receiving treatment: ______

1. Microplaces (crime hot spots, specific housing project, etc.)
2. Small police‐defined units (such as beats)
3. Larger police‐defined units (such as districts or sectors)
4. Neighborhood or community level
5. City or town level
6. State level
7. Individuals
8. Other (specify) _____________

33. What is the exact unit receiving treatment?_________________________

*The following question refers to the units not receiving treatment*

34. Units NOT receiving treatment: ______

1. Microplaces (crime hot spots, specific housing project, etc.)
2. Small police‐defined units (such as beats)
3. Larger police‐defined units (such as districts or sectors)
4. Neighborhood or community level
5. City or town level
6. State level
7. Individual
8. Other (specify) _____________

35. What were the casual hypotheses tested in this study?__________________

36. Please identify any theories from which the causal hypotheses were derived.

________________________________________________________________

________________________________________________________________

***Outcomes reported** (Note that for each outcome, a separate coding sheet is required)*

37. How many crime/alternative outcomes are reported in the study? _____

38. What is the specific outcome recorded on this coding sheet?___________

39. Was it the primary outcome of the study? _______

1. Yes
2. No
3. Cannot tell/researcher did not prioritize outcomes

40. Was this initially intended as an outcome of the study? ______

1. Yes
2. No (explain)
3. Cannot tell

41. If no, explain why:

________________________________________________________________

________________________________________________________________

***Unit of analysis***

42. What was the unit of analysis for the research evaluation?

1. Individuals
2. Microplaces (crime hot spots, specific housing project, etc.)
3. Small police‐defined units (such as beats)
4. Larger police‐defined units (such as districts or sectors)
5. Neighborhood or community level
6. City or town level
7. State level
8. Other (specify) _____________

43. How many units of analysis are there for the intervention in the study? ______

44. Did the researchers collect nested data within the unit of analysis?

1. Yes
2. No

***Dependent Variable***

45. What type of data was used to measure the outcome covered on this coding sheet? ____

1. Official data (from the police)
2. Researcher observations
3. Self‐report surveys
4. Other (specify) ___________________

46. If official data was used, what specific type(s) of data were used? (Select all that apply)

1. Calls for service (911 calls)/crime reports
2. Arrests
3. Incident reports
4. Level of citizen complaints
5. Other (specify)
6. N/A (official data not used)
7. Other (specify) ___________________

47. If researcher observations were used, what types of observations were taken? (Select all that apply)

1. Physical observations (e.g., observed urban blight, such as trash, graffiti)
2. Social observations (e.g., observed disorder, such as loitering, public drinking)
3. Other observations (specify)
4. N/A (researcher observations not used)
5. Other (specify) ___________________

48. If self‐report surveys were used, who was surveyed? (Select all that apply)

1. Residents/community members
2. Business owners
3. Elected officials
4. Government/social service agencies
5. Other (specify) ___________________
6. N/A (self‐report surveys not used)

49. Specifically identify the outcome covered on this coding sheet ____________

50. For the units of analysis in this study, what time periods were examined for the outcome covered on this coding sheet?

1. Yearly
2. Monthly
3. Weekly
4. Other researcher defined time periods (specify) _________________

51. What was the length in time of the follow‐up period after the intervention?

________________________________________________________________

52. Did the researcher assess the quality of the data collected?

1. Yes
2. No

52. Did the researcher(s) express any concerns over the quality of the data?

1. Yes
2. No

52. If yes, explain_____________________________________________

53. Does the evaluation data correspond to the initially stated problem? (i.e., if the problem is gang violence, does the evaluation data specifically look at whether gang violence changed?)

1. Yes
2. No

53. If no, explain the discrepancy:

________________________________________________________________

________________________________________________________________

**Effect size/Reports of statistical significance**

***Dependent Measure Descriptors***

54. Statistical analysis design: _____

1. Pretest comparison
2. Posttest comparison
3. Follow‐up comparison
4. N/A

*Sample Size*

55. Based on the unit of analysis for this outcome, what is the total sample size in the analysis?________

56. What is the total sample size of the treatment group (group that receives the response)?_______

57. What is the total sample size of the control group (if applicable)? _____

58. Was attrition a problem in the analysis for this outcome?

1. Yes
2. No

58. If attrition was a problem, provide details (e.g., how many cases were lost and why were they lost).

________________________________________________________________

________________________________________________________________

________________________________________________________________

59. What do the sample sizes above refer to?

1. Crimes
2. People
3. Geographic areas
4. Places
5. Other (specify) ________________

***Effect Size Data***

60. Raw difference favors (i.e., shows more success for):

1. Treatment group
2. Control group
3. Neither (exactly equal)
9. Cannot tell (or statistically insignificant report only)

61. Did a test of statistical significance indicate statistically significant differences between either the control and treatment groups or the pre and post tested treatment group? ____

1. Yes
2. No
3. Cannot tell
4. N/A (no testing completed)

62. Was a standardized effect size reported?

1. Yes
2. No

63. If yes, what was the effect size? ______

64. If yes, page number where effect size data is found ________

65. If no, is there data available to calculate an effect size?

1. Yes
2. No

66. Type of data effect size can be calculated from:

1. Means and standard deviations
2. *t*‐value or *F*‐value
3. Chi‐square (*df *= 1)
4. Frequencies or proportions (dichotomous)
5. Frequencies or proportions (polychotomous)
6. Other (specify) ________

*Means and Standard Deviations*

67. Treatment group mean. _____

67. Control group mean. _____

68. Treatment group standard deviation. _____

68. Control group standard deviation. _____

*Proportions or frequencies*

69. *n* of treatment group with a successful outcome. _____

69. *n* of control group with a successful outcome. _____

70. Proportion of treatment group with a successful outcome. _____

70. Proportion of treatment group with a successful outcome. _____

*Significance Tests*

71. *t*‐value _____

71. *F*‐value _____

71. Chi‐square value (*df *= 1) _____

*Calculated Effect Size*

72. Effect size ______

72. Standard error of effect size _____

**Conclusions made by the author(s)**

*Note that the following questions refer to conclusions about the effectiveness of the intervention in regards to the current outcome being addressed on this coding sheet.*

73. Conclusion about the impact of the intervention? _____

1. The authors conclude problem declined
2. The authors conclude the problem did not decline
3. Unclear/no conclusion stated by authors

74. Did the assessment find evidence of a geographic displacement of crime? _____

1. Yes
2. No
3. Not tested

75. Did the assessment find evidence of a temporal displacement of crime? _____

1. Yes
2. No
3. Not tested

76. Did the author(s) conclude that the pulling levers intervention was beneficial? _____

1. Yes
2. No
3. Can't tell

77. Did the author(s) conclude there a relationship between the pulling levers intervention and a reduction in crime? _____

1. Yes
2. No
3. Can't tell

78. Who funded the intervention?

________________________________________________________________

________________________________________________________________

79. Who funded the evaluation research?

________________________________________________________________

________________________________________________________________

80. Were the researchers independent evaluators?

1. Yes
2. No

80. If no, explain the nature of the relationship:

________________________________________________________________

________________________________________________________________

81. Additional notes about conclusions:

________________________________________________________________

82. Additional notes about study:

________________________________________________________________

## APPENDIX C DESCRIPTIVE NARRATIVE REVIEW OF INCLUDED STUDIES

### Operation Ceasefire in Boston, MA

The Boston Gun Project was a problem‐oriented policing enterprise expressly aimed at taking on a serious, large‐scale crime problem—homicide victimization among young people in Boston in the 1990s. The trajectory of the Boston Gun Project, and the resulting Operation Ceasefire intervention, is by now well‐known and extensively documented (Braga et al., 2001; Kennedy, 1997, 2006; Kennedy et al., 1996). Briefly, a working group of law enforcement personnel, youth workers, and Harvard researchers diagnosed the youth violence problem in Boston as one of patterned, largely vendetta‐like hostility amongst a small population of chronic offenders, and particularly among those involved in 61 loose, informal, mostly neighborhood‐based “gangs.” These 61 gangs consisted of some 1,300 members, representing less than 1% of the city's youth between the ages of 14 and 24. Although small in number, these gangs were responsible for more than 60% of youth homicide in Boston.

The Operation Ceasefire focused deterrence strategy was designed to prevent violence by reaching out directly to gangs, saying explicitly that violence would no longer be tolerated, and backing up that message by “pulling every lever” legally available when violence occurred (Kennedy 1997). The chronic involvement of gang members in a wide variety of offenses made them, and the gangs they formed, vulnerable to a coordinated criminal justice response. The authorities could disrupt street drug activity, focus police attention on low‐level street crimes such as trespassing and public drinking, serve outstanding warrants, cultivate confidential informants for medium‐ and long‐term investigations of gang activities, deliver strict probation and parole enforcement, seize drug proceeds and other assets, ensure stiffer plea bargains and sterner prosecutorial attention, request stronger bail terms (and enforce them), and bring potentially severe federal investigative and prosecutorial attention to gang‐related drug and gun activity. Simultaneously, youth workers, probation and parole officers, and later churches and other community groups offered gang members services and other kinds of help.

These partners also delivered an explicit message that violence was unacceptable to the community and that “street” justifications for violence were mistaken. The Ceasefire Working Group delivered this message in formal meetings with gang members (known as “forums” or “call‐ins”), through individual police and probation contacts with gang members, through meetings with inmates at secure juvenile facilities in the city, and through gang outreach workers. The deterrence message was not a deal with gang members to stop violence. Rather, it was a promise to gang members that violent behavior would evoke an immediate and intense response. If gangs committed other crimes but refrained from violence, the normal workings of police, prosecutors, and the rest of the criminal justice system dealt with these matters. But if gang members hurt people, the Working Group concentrated its enforcement actions on their gangs.

The Ceasefire “crackdowns” were not designed to eliminate gangs or stop every aspect of gang activity, but to control and deter serious violence. To do this, the Working Group explained its actions against targeted gangs to other gangs, as in “this gang did violence, we responded with the following actions, and here is how to prevent anything similar from happening to you.” The ongoing Working Group process regularly watched the city for outbreaks of gang violence and framed any necessary responses in accord with the Ceasefire strategy. As the strategy unfolded, the Working Group continued communication with gangs and gang members to convey its determination to stop violence, to explain its actions to the target population, and to maximize both voluntary compliance and the strategy's deterrent power.

The DOJ‐sponsored evaluation of the impact of Operation Ceasefire used a nonrandomized quasi‐experimental design to compare youth homicide trends in Boston to youth homicide trends in other major cities in the United States and large New England cities (Braga et al., 2001). The key outcome variable was the monthly number of homicide victims ages 24 and under between January 1, 1991 and May 31, 1998. The within‐Boston program impact assessment was supplemented by analyses of Ceasefire's effect on the monthly number of citywide gun assault incidents, citywide shots‐fired calls for service, and youth gun assault incidents in one high‐risk policing district. Poisson and negative binomial regression models, controlling for secular trends, seasonal variations, Boston youth population trends, Boston employment rate trends, robbery trends, adult homicide trends, and youth drug arrest trends, were used to estimate the effect of Ceasefire on the outcome variables. The impact of Ceasefire was estimated using a dummy variable with June 1996 selected as the commencement of the postimplementation period.

The Ceasefire evaluation concluded that the program was associated with statistically significant reductions in youth homicide and the other indicators of serious gun violence in Boston. Controlling for the other covariates, the evaluation reported that Ceasefire was associated with a 63% reduction in the monthly count of youth homicides, a 25% reduction in the monthly count of citywide gun assault incidents, a 32% reduction in the monthly count of citywide shots‐fired calls for service, and a 44% reduction in the monthly count of youth gun assaults in selected high‐risk district (Braga et al., 2001; see also Piehl et al., 2000). In a companion paper, Piehl et al. (2003) closely analyzed the monthly counts in the youth homicide time series to determine whether the timing of the implementation of Ceasefire coincided with the start of the significant decrease in Boston youth homicides. The authors developed an econometric model that evaluated all possible monthly break points in the time series to identify the maximal monthly break point associated with a significant structural change in the trajectory of the time series. Controlling for trends and seasonal variations, the timing of the “optimal break” in the time series was in the summer months after Ceasefire was implemented.

The DOJ‐sponsored evaluation then conducted a comparative analysis of youth homicide trends in 39 of the most populous cities in the United States and 29 New England cities with populations of more than 60,000 residents (Braga et al., 2001). Using count regression models that controlled for trends, seasonal variations, and serial autocorrelation, the research found that only three cities (Dallas, TX; Jacksonville, FL; and Virginia Beach, VA) had significant reductions in the monthly count of youth homicides that coincided with the implementation of Ceasefire in Boston and an additional four cities (Los Angeles, CA; New York City, NY; Philadelphia, PA; and Tucson, AZ) had significant reductions in the monthly count of youth homicides at some point within the time series. Further examination of the youth homicide trends in these cities, however, revealed trajectories that looked distinct from the trajectory of Boston youth homicide over the same time period. As such, the researchers concluded that Boston's youth homicide reduction associated with Operation Ceasefire was distinct when compared to trends in most major U.S. cities.

The DOJ evaluation has been reviewed by a number of researchers and the relationship between the implementation of Ceasefire and the trajectory youth homicide in Boston during the 1990s has been closely scrutinized. Fagan (2002) suggested that some of the decrease in homicide may have occurred without the Ceasefire intervention in place as violence was decreasing in most major U.S. cities. In support of this perspective, Fagan's (2002) presented a simple time‐series graph on youth gun homicide in Boston and in other Massachusetts cities that suggested a general downward trend in gun violence may have existed before Operation Ceasefire was implemented. Using growth‐curve analysis to examine predicted homicide trend data for the 95 largest U.S. cities during the 1990s, Rosenfeld et al. (2005) found some evidence of a sharper youth homicide drop in Boston than elsewhere but suggest that the small number of youth homicide incidents precludes strong conclusions about program effectiveness based on their statistical models. However, in his review of their analysis, Richard Berk (2005) raised a number of statistical and methodological concerns with the analysis developed by Rosenfeld and his colleagues. Ludwig (2005) suggested that Ceasefire was associated with a large drop in youth homicide but, given the complexities of analyzing city‐level homicide trend data, there remained some uncertainty about the extent of Ceasefire's effect on youth violence in Boston. Morgan and Winship's (2007) review of the DOJ evaluation concluded that the analysis was a “very high‐quality example” of how to conduct an interrupted time series analysis of program impact and further noted “they offer four types of supplemental analysis … which can be used to strengthen the warrant for causal assertion” (p. 252).

The National Academies' Panel on Improving Information and Data on Firearms (National Research Council, 2005) concluded that the Ceasefire evaluation was compelling in associating the intervention with the subsequent decline in youth homicide. However, the Panel also suggested that many complex factors affect youth homicide trends and it was difficult to specify the exact relationship between the Ceasefire intervention and subsequent changes in youth offending behaviors. While the DOJ‐sponsored evaluation controlled for existing violence trends and certain rival causal factors such as changes in the youth population, drug markets, and employment in Boston, there could be complex interaction effects among these factors not measured by the evaluation that could account for some meaningful portion of the decrease. The evaluation was not a randomized, controlled experiment. Therefore, the nonrandomized control group research design cannot rule out these internal threats to the conclusion that Ceasefire was the key factor in the youth homicide decline.

### Indianapolis Violence Reduction Partnership (IVRP) in Indianapolis, IN

The IVRP working group was comprised of Indiana University researchers and federal, state, and local law enforcement agencies (McGarrell & Chermak, 2003). During the problem analysis phase, the researchers examined 258 homicides from 1997 and the first 8 months of 1998 and found that a majority of homicide victims (63%) and offenders (75%) had criminal and/or juvenile records. Those with a prior record often had a substantial number of arrests. The working group members followed the structured qualitative data gathering exercises used in Boston to gain insight on the nature of homicide incidents. The qualitative exercise revealed that 59% of the incidents involved “groups of known chronic offenders” and 53% involved drug‐related motives such as settling business and turf disputes (McGarrell & Chermak, 2003). It is worth noting that the terminology “groups of known chronic offenders” was initially used because, at that point in time, there was not a consensual definition of “gang” and the reality of much gang activity in Indianapolis was of a relatively loose structure (McGarrell & Chermak, 2003).

The working group developed two sets of overlapping strategies. First, the most violent chronic offenders in Indianapolis were identified and targeted for heightened arrest, prosecution, and incarceration (McGarrell & Chermak, 2003). Second, the working group engaged the pulling levers approach to reduce violent behavior by gangs and groups of known chronic offenders (McGarrell & Chermak, 2003). The IVRP strategy implemented by the Indianapolis working group closely resembled the Boston version of pulling levers. The communications strategy, however, differed in an important way. The deterrence and social services message was delivered in meetings with high‐risk probationers and parolees organized by neighborhoods. Similarly, home visits by probation and parolees were generally organized by neighborhood. As the project progressed, when a homicide or series of homicides involved certain groups or gangs, the working group attempted to target meetings, enforcement activities, and home visits on the involved groups or gangs (McGarrell & Chermak, 2003).

The evaluation of the IVRP gang VRS used a nonrandomized quasi‐experimental design to compare homicide incident trends in Indianapolis to homicide incident trends in six Midwestern cities (McGarrell et al., 2006). The six comparison cities included Cincinnati (OH), Cleveland (OH), Columbus (OH), Kansas City (MO), Louisville (KY), and Pittsburgh (PA). For all seven cities, the key outcome variable was the monthly number of homicide incidents between January 1, 1997 and June 30, 2001. The evaluation used AutoRegressive Integrated Moving Average (ARIMA) models to analyze the city time series data.

The impact of the IVRP strategy was estimated using a dummy variable with April 1999 selected as the commencement of the postintervention period (McGarrell et al., 2006). The ARIMA analyses of the Indianapolis homicide time series estimated that the IVRP intervention was associated with a statistically significant 34% reduction in monthly numbers of homicides. The ARIMA models analyzing the other cities' homicide time series did not report any statistically significant associations between the timing of IVRP and subsequent decreases in monthly homicide numbers. In a subsequent analysis of Indianapolis homicide time series data, Corsaro and McGarrell (2009) used ARIMA models to analyze the impact of IVRP on gang and nongang homicides. The analyses found a statistically significant 38% reduction in gang homicides following the implementation of IVRP and did not find a statistically significant reduction in the nongang homicides during the postintervention time period. Since IVRP was explicitly designed to reduce gang violence, the authors concluded that these results support the position that the intervention was indeed having the desired effects on violent gang offending.

### Operation Peacekeeper in Stockton, CA

Beginning in mid‐1997, criminal justice agencies in Stockton began experimenting with the pulling levers approach to address a sudden increase in youth homicide. The Stockton Police Department and other local, state, and federal law enforcement agencies believed that most of the youth violence problem was driven by gang conflicts and that the pulling levers approached used in Boston might be effective in reducing Stockton's gang violence problem. The strategy was implemented by the Stockton Police Department's Gang Street Enforcement Team and grew into what is now known as “Operation Peacekeeper” as more agencies joined the partnership (Wakeling, 2003).

The Peacekeeper intervention was managed by a working group of line‐level criminal justice practitioners; social service providers also participated in the working group process as appropriate. When street gang violence erupted or when it came to the attention of a working group member that gang violence was imminent, the working group followed the Boston model by sending a direct message that gang violence would not be tolerated, pulling all available enforcement levers to prevent violence, continuing communications, and providing social services and opportunities to gang members who want them.

The Operation Peacekeeper evaluation used a nonrandomized quasi‐experimental design to compare gun homicide trends in Stockton to gun homicide trends in eight other midsized California cities (Braga, 2008b). The eight comparison California cities included Anaheim, Bakersfield, Fresno, Long Beach, Oakland, Riverside, Sacramento, and Santa Ana. For each of the nine cities included in the evaluation, the key outcome variable was the monthly number of gun homicide victims between January 1, 1990 and December 31, 2005. The evaluation carefully analyzed the distributions of the dependent variables for each city's time series to determine the appropriate regression models for the impact assessment. Ordinary Least Squares (Santa Ana), maximum likelihood with an AR(1) autoregressive component (Long Beach, Oakland), negative binomial (Anaheim, Bakersfield, Fresno, Riverside, Stockton), and Poisson (Sacramento) regression models were used to analyze the city time series data.

Stockton's Operation Peacekeeper intervention was implemented in September 1997 and was operational until it was discontinued in December 2002 (Braga, 2008b). Multiple category dummy variables indicating the time periods when the Stockton Peacekeeper intervention was present or not were included in the regression models to estimate the trajectory of the monthly counts of gun homicide in each of the time series after Stockton implemented its gun violence reduction initiative. Controlling for existing linear and nonlinear trends, seasonal variations, and violent crime trends, the negative binomial regression analyzing the Stockton gun homicide time series estimated that the intervention was associated with a statistically significant 42% reduction in the monthly count of gun homicides. None of the comparison cities experienced a statistically significant reduction in the monthly count of gun homicides that coincided with the implementation of the Peacekeeper intervention in Stockton.

### Project Safe Neighborhoods (PSN) in Lowell, MA

Supported by funds from the U.S. Department of Justice‐sponsored PSN initiative, an interagency task force implemented a pulling levers focused deterrence strategy to prevent gun violence among Hispanic and Asian gangs in Lowell, MA in 2002 (Braga, McDevitt, & Pierce, 2006). The Lowell authorities used a pulling levers focused deterrence strategy that replicated Boston's Operation Ceasefire to prevent violence among Hispanic gangs. However, from the outset, they felt much less confident about their ability to prevent Asian gang violence by applying the same set of criminal justice levers to Asian gang members. During the intervention time period, the Lowell Police Department (LPD) had little reliable intelligence about Asian gangs in the city (Braga et al., 2006). The LPD had attempted to develop informants in the past but most these efforts had been unsuccessful.

Through PSN, the LPD increased its efforts to develop intelligence about the structure of the city's Asian gangs and particularly the relationship between Asian gang violence and ongoing illegal gambling that was being run by local Asian businesses. In Lowell, Cambodian and Laotian gangs were comprised of youth whose street activities were influenced by “elders” of the gang (Braga et al., 2006). Elders were generally long‐time gang members in their 30s and 40s that no longer engaged in illegal activities on the street or participated in street‐level violence with rival youth. Rather, these older gang members were heavily involved in running illegal gambling dens and informal casinos that were operated out of cafes, video stores, and warehouses located in the poor Asian neighborhoods of Lowell. The elders used young street gang members to protect their business interests and to collect any unpaid gambling debts. Illegal gaming was a very lucrative business that was much more important to the elders than any ongoing beefs the youth in their gang had with other youth (Braga et al., 2006). In contrast to acquiring information on individuals responsible for gun crimes in Asian communities, it was much easier to detect the presence of gambling operations through surveillance or a simple visit to the suspected business establishment.

The importance of illegal gaming to influential members of Asian street gangs provided a potentially potent lever to law enforcement in preventing violence. The authorities in Lowell believed that they could systematically prevent street violence among gangs by targeting the gambling interests of older members. When a street gang was violent, the LPD targeted the gambling businesses run by the older members of the gang. The enforcement activities ranged from serving a search warrant on the business that houses the illegal enterprise and making arrests to simply placing a patrol car in front of the suspected gambling location to deter gamblers from entering. The LPD coupled these tactics with the delivery of a clear message, “when the gang kids associated with you act violently, we will shut down your gambling business. When violence erupts, no one makes money” (Braga et al., 2006, p. 40). Between October 2002 and June 2003, the height of the focused attention on Asian gangs, the LPD conducted some 30 search warrants on illegal gambling dens that resulted in more than 100 gambling‐related arrests (Braga et al., 2006).

The evaluation of the PSN gang VRS used a nonrandomized quasi‐experimental design to compare fatal and nonfatal gun assault incident trends in Lowell to fatal and nonfatal gun assault incident trends in seven other Massachusetts cities and the entire State of Massachusetts (Braga et al., 2008). The seven comparison Massachusetts cities included Boston, Brockton, Fall River, Lynn, New Bedford, Springfield, and Worcester. For the State of Massachusetts and the eight cities included in the evaluation, the key outcome variable was the monthly number of gun assault incidents between January 1, 1996 and December 31, 2005. The evaluation carefully analyzed the distributions of the dependent variables for each time series to determine the appropriate regression models for the impact assessment. Maximum likelihood with an AR(1) autoregressive component (Boston, Springfield, and State of Massachusetts), negative binomial (Brockton, Lynn, New Bedford, Worcester), and Poisson (Lowell, Fall River) regression models were used to analyze the city time series data.

The impact of Lowell's PSN strategy was estimated using a dummy variable with October 2002 selected as the commencement of the postintervention period (Braga et al., 2008). Controlling for existing linear and nonlinear trends, seasonal variations, population changes, and violent crime trends, the Poisson regression model reported that the Lowell PSN intervention was associated with a statistically significant 44% reduction in the monthly count of gun assault incidents. Neither the comparison cities nor the State of Massachusetts experienced a statistically significant reduction in the monthly count of gun homicides that coincided with the implementation of the PSN intervention in Lowell.

### Cincinnati Initiative to Reduce Violence in Cincinnati, OH

In response to a disturbing increase in homicides between 2001 and 2006, Cincinnati's political leadership partnered with law enforcement officials, academics, medical professionals, street advocates, and community and business leaders, to form the Cincinnati Initiative to Reduce Violence (CIRV; Engel et al., 2010). Strategy development was assisted by Proctor and Gamble Co. in the hopes of strengthening institutionalization and sustainability (Engel, Tillyer, & Corsaro, 2013). Problem analyses suggested that violent street groups of active criminal offenders generated the bulk of homicides and shootings in Cincinnati; thus, members of criminally active street groups in Cincinnati were the target population for the pulling levers focused deterrence strategy. As described by Engel et al. (2010), Cincinnati implemented a group VRS that was modeled after the pulling levers focused deterrence strategy implemented in Boston and included law enforcement consequences for violence, along with social service opportunities and community engagement. In face‐to‐face offender notification meetings, police, community activists, political figures, civil rights activists, ex‐offenders, parents of murdered children, social service providers, medical personnel, and business, civic, and religious leaders told members of violent groups that the violence must stop, that there would be law enforcement consequences for the entire group if it did not, and that the community would support these consequences (Engel et al., 2010). The working group partners also told violent group members that there was social service help for all who wanted it.

The CIRV utilized Street Advocates who performed the role of violence interrupters. Despite Street Advocates suggesting that they disrupted 75 incidents from January 2009 to December 2010, problems emerged with this component of the intervention. Three Street Advocates were arrested while participating in the initiative leading to the suspension of violence interrupters in December 2010 and reduced funding for the overall initiative the following year (Engel et al., 2013).

Preliminary evaluations of the impact of the CIRV interventions yielded promising results (Engel et al., 2008, 2009, 2010), but a more complete assessment of the CIRV was completed by Engel et al. (2013), who extended the follow‐up period through December 2010. Due to the longer postintervention period, 28 call‐ins were included compared to 20 in the previous report and 568 group members attended compared to the 488 group members at the time of the previous report. From the implementation of the intervention in July 2007 through December 2010, enhanced law enforcement and sanctions were imposed on 17 gangs and groups.

For the evaluation, Engel et al. (2013) used monthly crime data from January 2004 through December 2010, as well as measures of social service provisions offered during the intervention from July 2007 through December 2010. Two dependent variables were examined: gang‐member‐involved (GMI) homicides and violent firearm offenses (fatal and nonfatal shootings). Engel et al. (2013) used a two‐stage analytic strategy. First, generalized linear modeling (GLM) pooled time series regression models with controls for trends and seasonal variations were used to estimate the effects of the CIRV over time (Engel et al., 2013). Second, fixed‐effects maximum likelihood regression models with a pooled cross‐section time series were used to parse out the effects of social service provisions from the larger intervention (Engel et al., 2013).

Pooled time series regression results suggest that the CIRV was significantly associated with reductions in GMI homicides at 24 months (38%) and 42 months (41%; Engel et al., 2013). By comparison, non‐GMI homicides increased non‐significantly 12% and 38% at 24 months and 42 months, respectively, postintervention. Additionally, violent firearm offenses were significantly lower by 22% at both 24 months and 42 months postintervention. For comparative purposes, violent non‐firearm offenses experienced a statistically nonsignificant 5% reduction both 24 months and 42 months following the intervention (Engel et al., 2013).

To tease out the effects of social services from the overall initiative, Engel and colleagues (2013) investigated whether the number of social service provisions offered to targeted individuals was related to GMI homicides and/or violent firearm offenses. Regression results indicate that social services were not significantly related to instantaneous changes in GMI homicides or violent firearm offenses (Engel et al., 2013). Furthermore, lagged effects of social service provisions at 1‐, 2‐, and 6‐month intervals were examined but not consistent relationship with outcome measures was observed (Engel et al., 2013).

### Operation Ceasefire in Newark, NJ

In a peer‐reviewed evaluation, researchers from the Violence Institute of New Jersey at the University of Medicine and Dentistry of New Jersey (UMDNJ) evaluated the Operation Ceasefire gang violence strategy in Newark, New Jersey (Boyle et al., 2010). The Newark Ceasefire strategy focused on preventing gun violence by individual gang members in a targeted “Ceasefire Zone.” According to Boyle et al. (2010), the Newark strategy blended the law enforcement actions developed by the Boston Ceasefire pulling levers strategy (Kennedy et al., 1996) with the public health violence prevention activities developed by CeaseFire Chicago (Skogan, Hartnett, Bump, & Dubois, 2008). Shooting teams of detectives from the Newark, Irvington, and New Jersey State police departments aggressively investigated fatal and nonfatal shootings in the Ceasefire Zone. Parole officers also closely monitored high‐risk individuals in the targeted area.

Drawing on the Chicago approach, Newark Ceasefire addressed risk and protective factors for individual gang members through five program components: public education, community mobilization, faith‐based leader involvement, youth outreach, and criminal justice system engagement. Ceasefire youth outreach workers attempted to change the way gang members thought about and reacted to violence and to connect them with available services and opportunities. While there were not any formal offender‐notification strategies in place, the participating law enforcement agencies, community groups, and outreach workers actively communicated with individual gang members to prevent retaliatory shootings and disrupt ongoing conflicts.

The Ceasefire Zone was a roughly two‐square mile section of Newark that experienced elevated levels of gun homicides and shooting incidents. The Newark Ceasefire intervention was implemented on May 11, 2005. The evaluation team used ARIMA models to examine nonfatal gunshot wound trends in the Ceasefire Zone, a comparison zone, and the remainder of the City of Newark minus the Ceasefire Zone (Boyle et al., 2010). The comparison zone was identified through spatial analyses of nonfatal gunshot wounds to identify an area of similar size with similar levels of gun violence in Newark and also matched to the Ceasefire Zone based on 2000 Census data on the number of block groups in each area, population, resident race and ethnicity, median resident age and household income, concentrated poverty, and vacant housing units.

In the Newark Ceasefire evaluation, the key outcome variable was the weekly number of nonfatal gunshot wound victims treated at the Trauma Center at University Hospital in Newark in the Ceasefire Zone, comparison zone, and remainder of Newark between January 1, 2004 and December 31, 2006 (Boyle et al., 2010). These victimizations were geocoded by the location of gunshot wounding and then aggregated into weekly counts in the larger areas in which the events were contained. The ARIMA model estimated that the Newark Ceasefire intervention was associated with a nonstatistically significant decrease in the weekly number of nonfatal gunshot wound victims in the Ceasefire Zone. The comparison zone also experienced a smaller, nonsignificant decrease in the weekly number of nonfatal gunshot victims and the remainder of Newark experienced a nonsignificant increase in the weekly number of nonfatal gunshot victims. As such, the evaluators concluded that Newark Ceasefire was not associated with any significant reductions in nonfatal gunshot wounds.

The researchers also used crime mapping software to examine potential crime displacement and diffusion effects in the areas immediately surrounding the Ceasefire Zone. While the researchers noted changes in the spatial distributions of nonfatal gunshot woundings in the areas surrounding the Ceasefire Zone, they concluded that their analyses could not link the development of new gun violence hot spots and “cold spots” to displacement and/or diffusion processes associated with the Ceasefire intervention.

### Operation Ceasefire in Los Angeles, CA

In March 1998, NIJ funded the RAND Corporation to develop and test strategies for reducing gun violence among youth in Los Angeles. In part, the goal was to determine which parts of the Boston Gun Project might be replicable in Los Angeles. In designing the replication, RAND drew a clear distinction between the process governing the design and implementation of the strategy (data‐driven policy development; problem solving, working groups) and the elements and design (pulling levers, collective accountability, retailing the message) of the Boston model. Processes, in theory, can be sustained and adaptive, and as such can be utilized to address dynamic problems. By singling out process as an important component, the RAND team hoped to make clear that process can affect program effectiveness independently of the program elements or the merits of the actual design (see Tita et al., 2003).

The Los Angeles replication was unique in several important ways. First, the implementation was not citywide, but only within a single neighborhood (Boyle Heights) within a single Los Angeles Police Department Division (Hollenbeck). The project site, Boyle Heights, had a population that was relatively homogenous. Well over 80% of the residents were Latinos of Mexican origin. The same was true for the gangs, many of which were formed prior to the Second World War. These gangs were clearly “traditional” gangs, with memberships exceeding a hundred members or more. The gangs were strongly territorial, contain age‐graded substructures, and are intergenerational in nature (Tita et al., 2003).

Unlike other cities where gang and group‐involved violence was a rather recent phenomenon, Los Angeles represented an attempt to reduce gun violence in a “chronic gang city” with a long history of gang violence, and equally long history of gang reduction strategies. The research team had to first convince members of the local criminal justice and at large community that the approach we were espousing differed in important ways from these previous efforts to combat gangs. And in fact it does—the RAND project was not about “doing something about gangs,” but rather “doing something about gun violence” in a community where gang members committed an overwhelming proportion of gun violence. The independent analysis of homicide files confirmed the perception held by police and community alike that gangs were highly overrepresented in homicidal acts. From 1995 to 1998, 50% of all homicides had a clear gang motivation. Another 25% of the homicides could be coded as “gang related” because they involved a gang member as a victim or offender, but were motivated for reasons other than gang rivalries.

Given the social organization of violence in Boyle Heights, the multidisciplinary working group fully embraced the pulling levers focused deterrence strategy developed in Boston. A high‐profile gang shooting that resulted in a double homicide in Boyle Heights triggered the implementation of the Operation Ceasefire intervention in October 2000. The processes of retailing the message were formally adopted, though it was mostly accomplished through personal contact rather than in a group setting. Police, probation, community advocates, street gang workers, a local hospital and local clergy were all passing along the message of collective accountability for gangs continuing to commit gang violence. Unfortunately, Tita et al. (2003) reported that the Los Angeles pulling levers intervention was not fully implemented as planned. The implementation of the Ceasefire program in the Boyle Heights was negatively affected by the well‐known Ramparts LAPD police corruption scandal and a lack of ownership of the intervention by the participating agencies.

Despite the implementation difficulties, the RAND Corporation evaluated the Operation Ceasefire pulling levers strategy to reduce gun violence among gangs in the Boyle Heights area of Los Angeles (Tita et al., 2003). In their evaluation, RAND researchers examined the effects of the pulling levers gang VRS on violent crime (homicides, attempted homicides, robberies, assaults, and kidnapings), “gang crime” (violent crime and terrorist threats, firearm discharge, vandalism, and graffiti committed by gang members), and gun crime (any of the above crimes that involved use of a firearm).

The RAND evaluation analyzed changes in their key outcome variables for three time periods across three comparison areas (Tita et al., 2004). The three time periods were the 6 months prior to the triggering event—the *preintervention* period; the 4 months in which all parts of the intervention were applied—the *suppression* period; and the 2 months in which only selected parts of the intervention were applied, such as heightened patrol of public housing units in the area and greater enforcement of probation and parole regulations—the *deterrence* period. The three comparison areas were (a) Boyle Heights compared with the remainder of the Hollenbeck area, (b) the five police reporting districts where the intervention was targeted compared with the remainder of Boyle Heights, and (c) the Census block groups comprising the turf of the targeted gangs compared with a group of Census block groups scattered throughout Hollenbeck that most closely matched the characteristics of the targeted area based on a propensity score analysis. In explaining the rationale for their research design, the RAND researchers reported.

A reduction in crime in the treatment areas greater than those in the comparison areas during the suppression period would help show the effects of all measures combined, whereas continuing reductions in the deterrence period would suggest that the intervention may have had some long‐term effects in changing behavior, or that short‐term application of some resources can produce a long‐term deterrence effect (although we recognize our measure of deterrence is confounded by the continuation of some suppression activities; Tita et al., 2004, pp. 24–25).

The evaluation used a variety of approaches to detect the effects of the Ceasefire intervention across the study time period (Tita et al., 2004). The RAND researchers used Bayesian analyses of the count‐based distributions of the outcome variables across these time periods in Boyle Heights relative to the remainder of Hollenbeck comparison and in the five targeted reporting districts relative to the remainder of Boyle Heights comparison. In their analyses of targeted Census block groups relative to matched comparison Census block groups, the RAND researchers used Probit and step‐wise linear regression models to define the matched comparison Census block groups. They then used a slightly more rigorous version of the “difference in differences” approach that assumed the level of crime followed a Poisson distribution and considered serial time trends to evaluate the effects of the Ceasefire intervention across these time periods (Tita et al., 2004).

Their statistical analyses revealed that gang crime in Boyle Heights decreased significantly compared with other regions of Hollenbeck during the suppression period of the intervention, and violent, gang, and gun crime all decreased significantly in the deterrence period. The analyses suggested that the significant reduction in gang crime may have begun in the suppression period. Violent crime, however, did not decrease significantly in the suppression period. In the five targeted police reporting districts, violent crime decreased significantly in comparison with the rest of Boyle Heights in the suppression and the deterrence periods, and gang crime decreased significantly in the suppression period. Neither gang crime in the deterrence period nor gun crime in the deterrence or suppression periods decreased significantly in comparison with the remainder of Boyle Heights. The RAND evaluation also reported that, in the Census block groups overlapping the targeted reporting districts, violent crime decreased significantly compared with the matched blocks (Tita et al., 2004). Their analyses also suggested that some of this significant reduction may have persisted into the deterrence period.

In addition to their analyses of the main effects of the intervention, RAND researchers examined the effects of the intervention on neighboring areas and gangs. Their analyses suggested a strong diffusion of violence prevention benefits emanating from the targeted areas and targeted gangs (Tita et al., 2004). In the 6 months after the intervention, the researchers reported in the six targeted Census block groups that violent crime had decreased by 34%, gang crime decreased by 28%, and gun crime decreased by 26%. In the 11 Census block groups immediately surrounding the targeted block groups, violent crime had decreased by 33%, gang crime decreased by 44%, and gun crime decreased by 28%. The RAND research team also examined gang crime by gangs not targeted by the Ceasefire intervention that were “socially tied” through conflicts and alliances to the target gangs. After the Ceasefire intervention was implemented, gang crimes committed by the targeted gangs and the nontargeted, socially tied gangs decreased by a matching 26%.

### PSN in Chicago, IL

The PSN was implemented in two adjacent police districts in Chicago's West Side where rates of murder and gun violence were more than four times higher than the city average in 2002. As described by Papachristos et al. (2007), the PSN team reasoned that the best way to address Chicago's homicide and gun violence problem was to craft intervention strategies focused on the population with a very high risk of being a victim or offender of gun violence in the targeted neighborhoods. Two principles guided the design and development of PSN interventions: (a) that enforcement efforts be highly specified and targeted to those most at risk of being a victim and offender of gun violence, and (b) that serious efforts should be directed toward changing the normative side of gun violence, that is, the reasons young men use guns and their attitudes toward the law and law enforcers. Following these principles the PSN team devised several law enforcement, community outreach, and offender notification forums and follow‐up re‐entry programs. The PSN interventions were implemented in May 2002.

A quasi‐experimental design was used to evaluate the impact of the various PSN programs on neighborhood‐level homicide rates in Chicago (Papachristos et al., 2007). As described, two adjacent police districts were nonrandomly selected from the city's 25 police districts as PSN treatment districts and, via propensity score matching procedures, two other police districts selected as near‐equivalent controls. Monthly and quarterly counts of homicide incidents between January 1999 and December 2004 were identified as the key outcome variables (Papachristos et al., 2007; Meares, Papachristos, & Fagan, 2009); however, the evaluation also analyzed monthly and quarterly counts of gun homicide incidents, gang homicide incidents, and aggravated assault incidents in the treatment districts relative to the control districts.

The research team analyzed the overall effects of the PSN treatment as well as the four interventions that comprised the PSN treatment: (a) increased federal prosecutions for convicted felons carrying or using guns, (b) the length of sentences associated with federal prosecutions, (c) supply‐side firearm policing activities (gun recoveries by ATF‐CPD gun teams), and (d) social marketing of deterrence and social norms messages through justice‐style offender notification meetings. In these offender notification meetings, randomly selected gun‐ and gang‐involved recently released former prison inmates returning to the treatment districts were informed of their vulnerability as felons to federal firearms laws, with stiff mandatory minimum sentences; offered social services; and addressed by community members and ex‐offenders. Using individual growth curve regression models, the research team found that the PSN treatment was associated with a statistically significant 37% reduction in the number of homicides in the treatment district relative to the control districts. The overall PSN treatment was also associated with statistically significant decreases in gun homicide incidents and aggravated assault incidents, and a nonstatistically significant decrease in gang homicide incidents.

The PSN intervention that generated the largest, statistically significant effect on decreased homicide in the treatment districts relative to control districts was the offender notification forums. In short, the greater the proportion of offenders who attended the forums, the greater the decline in treatment district levels of homicide. Increased federal prosecutions and the number of guns recovered by the gun teams were associated with modest but statistically significant declines in homicides in the treatment districts relative to the control districts. Getting more guns off the street and prosecuting more offenders federally for gun crimes were associated with small but meaningful homicide decreases. The length of sentences associated with federal prosecutions was not associated with the observed homicide decreases.

In a supplemental unpublished analysis, Fagan et al. (2008) analyzed recidivism rates of individuals who participated in the PSN notification forums. Using survival analyses, the authors found that those who attended a PSN forum were 30% less likely to be rearrested relative to a comparison group of similar recently released individuals from the same neighborhood. The program diminished recidivism levels for both gang and nongang members and seemed to be particularly effective for individuals who had only one prior felony conviction.

In an alternative assessment, Wallace et al. (2016) focused on the offending behaviors of individuals after they attended a PSN offender notification forum. Two types of survival analyses were utilized to investigate participant recidivism: Cox proportional hazard models and competing risk hazard models. Forum participants had a recidivism hazard of approximately 43% less the hazard of nonparticipants from the intervention neighborhood and from nontreated control neighborhoods (Wallace et al., 2016). Additionally, forum participants had 30% lower hazard for committing a new offense compared to control groups. Lastly, results suggested that attending a PSN forum was associated with lower hazards for committing certain types of offenses (weapons and murder) relative to comparison groups (Wallace et al., 2016).

### Drug Market Intervention (DMI) in Nashville, TN

In peer‐reviewed publication, Corsaro, Brunson, and McGarrell (2010) evaluated the impact of a pulling levers focused deterrence strategy to reduce crime and drug‐related crime problems associated with an illegal drug market operating in the McFerrin Park neighborhood of Nashville, Tennessee. Drawing on similar intervention conducted in High Point, North Carolina (Kennedy, 2009), the project employed a joint police‐community partnership to identify individual offenders, notify them of the consequences of continued dealing, provide supportive services through a community‐based resource coordinator, and convey an uncompromising community norm against drug dealing. This application of focused deterrence is generally referred to as the “Drug Market Intervention” (DMI) strategy.

The DMI seeks to shut down overt drug markets entirely (Kennedy, 2009). Enforcement powers are used strategically and sparingly, employing arrest and prosecution only against violent offenders and when nonviolent offenders have resisted all efforts to get them to desist and to provide them with help. Through the use of “banked” cases, the strategy makes the promise of law enforcement sanctions against dealers extremely direct and credible, so that dealers are in no doubt concerning the consequences of offending and have good reason to change their behavior. The strategy also brings powerful informal social control to bear on dealers from immediate family and community figures. The strategy organizes and focuses services, help, and support on dealers so that those who are willing have what they need to change their lives. Each operation also includes a maintenance strategy.

The strategy was implemented in March 2008 and the evaluation examined outcome data for the time period of March 2005 through April 2010. The evaluation measured the effects of the DMI intervention on three outcome variables: drugs and narcotics offenses, UCR Type I Offenses, and calls for service. The researchers analyzed the aggregated monthly number of these outcome variables for the following Nashville areas: (a) the McFerrin Park target neighborhood to assess the local effect; (b) adjoining, contagious areas to the McFerrin Park neighborhood to assess whether a local displacement or a diffusion of benefits occurred; and (c) the remainder of Davidson County, once the target and adjoining areas were subtracted from the county totals for general trend comparison purposes.

A mixed methods approached was utilized to assess the impact of the Nashville DMI. Poisson regression models controlling for trends and seasonal variations with Bonferroni *p* value corrections were used to analyze trends in the treatment, adjoining, and comparison areas. Quantitative analyses were supplemented with in‐depth interviews with 44 target neighborhood to learn their perception of the intervention.

Regression results indicated that drug and narcotics offenses declined by 56% during the postintervention period (26 months), and a 38% reduction occurred in the adjoining area and only a 3% reduction in the remainder of the city (Corsaro et al., 2010). There were no statistically significant changes in UCR Type I offenses between pre and postintervention periods in the target neighborhood (4% reduction), adjoining area (20% reduction), or remainder of the city (3% increase; Corsaro et al., 2010). Calls for service were significantly lower by 13% in the target neighborhood postintervention, whereas nonsignificant reductions of 4% and 2% occurred in the adjoining area and remainder of the city, respectively (Corsaro et al., 2010).

Qualitative results suggested that most interviewees perceived crime as less of a problem in their neighborhood at the time of the interview compared to previous years (Corsaro et al., 2010). Specifically, drug market activity was noted by most residents as reducing the most, a reduction that newer residents attributed to police efforts and longer‐tenured residents attributed to the removal of public housing units from the neighborhood (Corsaro et al., 2010).

### DMI in Rockford, IL

Corsaro et al. (2009) evaluated the impact of a pulling levers focused deterrence strategy to reduce crime and disorder problems associated with an illegal drug market operating in the Delancey Heights neighborhood of Rockford, Illinois. Like the Nashville strategy described above, this research and development study was a replication of the High Point, North Carolina DMI (Kennedy, 2009). The strategy was implemented in May 2007 and the evaluation examined outcome data for the June 2006 through June 2008 time period. The evaluation measured the effects of the DMI intervention on two outcome variables: violent crime (the aggregated number of homicide, rape, kidnaping, robbery, and aggravated assault incidents) and nonviolent crime (the aggregate number of property, drug, and nuisance crime incidents). The researchers analyzed the aggregated monthly number of these outcome variables for the Delancey Heights neighborhood and for the remainder of Rockford without Delancey Heights.

Hierarchical generalized linear growth curve regression models with a dummy variable to represent the implementation of the DMI strategy were used to analyze trends in the treatment and comparison areas. The analyses reported that the DMI intervention was associated with a statistically significant 22% reduction in nonviolent offenses and a nonstatistically significant reduction in violent offenses in the Delancey Heights target neighborhood. The evaluation did not find any significant reductions in either violent offenses or nonviolent offenses in the remainder of Rockford. Corsaro et al. (2009) also presented qualitative data from interviews with 34 adult residents from the Delancey Heights neighborhood. The authors reported that the majority of the residents interviewed noted considerable crime and disorder improvements in their neighborhood after the DMI was implemented.

### Operation Ceasefire in Rochester, NY

In response to high levels of homicide, Rochester sought a data‐driven response to rampant violence. Many homicides were identified as involving groups that were also involved in selling drugs and other criminal activity (Delaney, 2006). This led to three attempts to implement a “pulling levers” Ceasefire model. After word of early success in Boston began to spread, the U.S. Attorney's Office of Western New York took the lead in Rochester's first attempt to implement a Ceasefire model in 1998. Delaney (2006) noted two primary reasons that led to the demise of this effort: (a) enforcement actions were never taken against groups that continued to engage in violence and (b) the intervention's deterrence message was actively disseminated to juveniles who were not involved in gangs or serious violence which undermined the credibility of the message. The second attempt to implement a Ceasefire model came in 2002–2003 and was resurrected by a team participating in the federal Strategic Approaches to Community Safety Initiatives (SACSI) program. Rather than groups, this second iteration targeted individual high‐rate offenders but similar to its predecessor, enforcement actions failed to be taken against continuing offenders.

Despite previous failings, a third attempt was made and Rochester Police Department was finally able to lead a successful implementation of the Ceasefire model with the first call‐in during October 2003 marking the official beginning of the postintervention period (Delaney, 2006). Unlike the two preceding attempts, the third iteration of Ceasefire was successfully able to carry out enforcement actions and used actions taken against the “Thurston Zoo” gang as an example to other criminally active groups. The intervention's message was delivered to identified groups at “call‐in” meetings, where attendees were informed of the increased attention from criminal justice agencies that will ensue if they continue to be committing homicide (Delaney, 2006).

An interrupted time‐series model was used to estimate the impact of the intervention on crime (Delaney, 2006). In addition to investigating the impact of the intervention on overall homicide, gun assaults, and gun robberies, the effects of the intervention were also examined for a specific high‐risk demographic for violent victimization: black males ages 15 through 30. Whether the intervention had a delayed effect was also assessed using 1‐month increments for a period of 4 months postintervention.

Controlling for trends and seasonal variation, regression results suggested at 1‐, 3‐ and 4‐month lags that the intervention was associated with statistically significant 25% and 27% reductions in homicide and gun robbery, respectively, involving black male victims ages 15 to 30; however, no significant reduction in gun assault victimization was found for that high‐risk population (Delaney, 2006). No significant relationship effect was found for overall homicide, gun assault, and gun robbery.

Importantly, Delaney (2006) noted a number of concerns pertaining to Rochester's Operation Ceasefire. First, a separate violent reduction strategy called Project IMPACT was implemented in violent crime hotspots throughout the city and overlapped with when Ceasefire was taking place. Second, problems with interagency communication contributed to challenges in conducting enforcement actions and selecting potential targets for the intervention. Third, some law enforcement personnel were asked to deliver the deterrence message on the street but were not fully aware of the details of the intervention. Lastly, despite the continued presence of the initiative, homicide rates increased in the first half of 2005 which was beyond the time period for this evaluation.

### DMI in High Point, NC

There were two primary goals of the drug market intervention in High Point, NC: (a) closing selected open‐air drug markets and (b) reducing violence associated with those targeted markets (Kennedy, 2009). Previous assessments of the High Point DMI suggested improved perceptions of crime among residents (Frabutt, Gathings, Jackson, and Buford, 2005) and more challenges for narcotic officers attempts to conduct undercover drug buys (Kennedy & Wong, 2009); however, previous evaluations did not estimate the intervention's effect on violent crime with empirical rigor (Corsaro et al., 2012).

Rolling implementation of the High Point DMI took place from 2004 through 2007 (Kennedy & Wong, 2009). For each site, collaborative investigations and surveillance of key offenders lasted between 1 and 3 months. Four call‐in meetings were held with offenders from four different neighborhoods over the course of the intervention. Key offenders with prior felony convictions were arrested while nonviolent offenders with no previous felony convictions were selected to participate in the interventions (Corsaro et al., 2012). In total, 83 dealers were identified across the four intervention locations; 20 were arrested and 63 were selected to participate in a call‐in (Corsaro et al., 2012). At the call‐in, the message communicated to participants consisted of deterrence, social services and opportunity, and changing norms.

Evaluation of the High Point DMI used annual violent crime data (homicide, rape and sexual offenses, assaults, and robberies) from 1998 through (Corsaro et al., 2012). Corsaro et al. (2012) evaluated the DMI in two stages. First, count‐based difference‐in‐difference panel regression models were used to estimate the impact of the intervention on census blocks in treatment areas relative to comparison units matched via propensity score analyses. Second, group‐based trajectory analyses (GBTA) were completed to estimate group‐specific treatment effects (Corsaro et al., 2012).

Regression results suggested that violent crime in targeted neighborhoods decreased significantly (*p *< .001) by 18% in the postintervention period compared to the preintervention period (Corsaro et al., 2012). To assess whether displacement occurred, violent crime in the 59 nearest neighboring census blocks to the intervention sites were compared to the remainder of the city that was not treated. Although violent crime in neighborhoods surrounding intervention sites increased approximately 14%, the change was not significantly distinct relative levels of violence in the remainder of the nontreated city (Corsaro et al., 2012). Additionally, results indicated violent crime in targeted neighborhoods decreased significantly by 13% postintervention relative to their matched comparison sites (Corsaro et al., 2012).

For GBTA, three‐groups were determined to be appropriate for categorizing census blocks. Using results from previous propensity score analyses, trajectory groups in the treated area were compared to trajectory groups in matched comparison areas (Corsaro et al., 2012). Chronic High Trajectory census blocks in the target area experienced a statistically significant 17% decrease in violent crime relative to matched comparison census blocks under the same trajectory classification. Violent crime in treated census blocks within the Moderate Stable Trajectory group decreased 11% compared to matched comparison groups within the same trajectory; however, this divergence was not statistically significant (Corsaro et al., 2012). In contrast, violent crime the treated census blocks in the Negligible Trajectory group increased significantly relative to matched comparison census block and trajectory, an increase that equated to one additional incident per year (Corsaro et al., 2012).

In subsequent analyses, the effectiveness of the High Point DMI was examined by specific intervention sites and across different outcomes (Corsaro, 2013; Saunders et al., 2015). Corsaro (2013) used monthly incident data from January 1998 through August 2009 to analyze the effects of the DMI on violent crime, property crime, and drug and disorder crime. Autoregressive Poisson regression models were used to estimate the unique effects of the intervention in each of the four target neighborhoods. One intervention site (West End) experienced significant declines across all three categories of offenses whereas one intervention site (East Central) experienced no significant declines in any of the offense categories. Two intervention sites (Daniel Brooks and Southside) experienced significant (*p *< .05) 18% reductions in property crime but only marginally significant or nonsignificant changes in remaining offense types. Further analysis suggested that declines in property crime were likely attributable to similar declines citywide (Corsaro, 2013).

Saunders et al. (2015) evaluated the High Point DMI after it was implemented in a fifth intervention site (Washington). Synthetic control weights were imported into a difference‐in‐difference negative binomial regression model to estimate the effectiveness of the intervention across four outcomes over a 12‐month follow‐up period (Saunders et al., 2015). Overall, the intervention was associated with significant reductions in calls for service (16%) and violent crime (34%) but was not significantly related to drug crime or general crime reports (Saunders et al., 2015). In terms of impacts on specific intervention sites, all four outcome measures decreased in one treatment location (West End) and three of four outcomes decreased in another treatment location (Washington). The remaining three intervention sites generally experienced declines in each of the outcome measures but not at a statistically significant level (Saunders et al., 2015). Additionally, Saunders et al. (2015) found little evidence of displacement associated with the intervention. The authors concluded that evidence from a synthetic control model suggests the intervention produced long‐term reductions of crime and its effects may be larger than suggested by previous evaluations (Saunders et al., 2015).

### DMI in Peoria, IL

Drawing on initiatives in High Point, NC, Nashville, TN, and Rockford, IL, the Peoria Police Department implemented a “pulling levers” strategy to address crime associated with an open‐air drug market (Corsaro & Bruson, 2013). The intervention occurred in a single neighborhood that was identified as being associated with disproportionately high crime rates.

In March 2009, police began conducting surveillance and gathering intelligence on 29 suspected drug dealers (Corsaro & Brunson, 2013). This investigation led the arrest of 23 of the 29 dealers in October 2009, who were then subjected to enhanced prosecution. The six remaining suspected drug dealers were selected to participate in a call‐in meeting. At the November 2009 call‐in, actions taken against the 23 dealers recently arrested were used as examples to those in attendance (Corsaro & Brunson, 2013). Attendees were delivered a stern message that further drug dealing would not be tolerated and increased enforcement and sanctions will be imposed on anyone who reoffends. In addition to the deterrence message, participants met with social service providers and local community leaders who encouraged reintegration into the community (Corsaro & Brunson, 2013).

A mixed methods approach was used to evaluate the Peoria “pulling levers” drug market intervention. First, an ARIMA approach was used to model the effects of the intervention on the time series (Corsaro & Brunson, 2013). Four outcome measures were examined in this study: violent crime, property crime, drug and disorder crime, and total calls for service. Monthly counts of these variables were analyzed from January 2006 through December 2010 (Corsaro & Brunson, 2013). Second, surveys were used to measure local residents' perceptions of the intervention and whether they perceived any changes in drug offending in the 6 months prior to completing the survey (Corsaro & Brunson, 2013).

Results from the interrupted time‐series model indicated no statistically significant (*p *> .05) relationship between the intervention and any of the four outcome variables. The intervention was positively related to violent and property crime, and was inversely related to drug/disorder crime and total calls for service (Corsaro & Brunson, 2013).

Although local media covered the November 2009 call‐in, survey results indicated that only a minority of residents (31%) were even aware the intervention took place in their neighborhood (Corsaro & Brunson, 2013). In terms of the perceived effectiveness of the intervention, the majority (66%) of those familiar with the intervention thought it had no impact. As for perceptions of crime overall in the past 6 months, 32% thought crime was less of a problem, 36% thought crime was about the same, and 32% thought crime was more of a problem (Corsaro & Brunson, 2013). Specifically, 49% of respondents believed drug sales were about the same over the past 6 months, whereas 28% though drug sales were less of a problem and 23% believed drug sales were more of a problem (Corsaro & Brunson, 2013).

### Operation Ceasefire II in Boston, MA

“Pulling levers” focused deterrence strategies originated in Boston, MA in the form of Operation Ceasefire. This intervention produced promising results at reducing gang‐motivated homicides but was disbanded by the year 2000. After the program ended, Boston experienced a steady increase in gang‐motivated homicides (Braga et al., 2014). To counter this re‐emerging problem, a Ceasefire approach was brought back and implemented citywide.

This second iteration of the Boston Ceasefire model targeted 19 gangs from January 2007 through December 2010 (Braga et al., 2014). Efforts to implement a Ceasefire model were made in November 2006 through an interagency coalition hosting a call‐in with 22 members of the Lucerne Street Doggz gang. However, although detectives and officers from a BPD district (B‐3) initiated the intervention, the full support of BPD needed to carry out enforcement actions was not in place until January 2007 at the directive of newly appointed Police Commissioner Davis (Braga et al., 2014). After the interagency working group was firmly established, the Lucerne Street Doggz gang became the target of the first enforcement action of the second iteration of Boston's Operation Ceasefire (Braga et al., 2014).

A nonrandomized quasi‐experimental design was used to compare trends in serious violence among gangs targeted by the intervention to comparison gangs matched via propensity score analyses. Because of general deterrent effect sought by “pulling levers” focused deterrence strategies, which violates the “stable unit treatment value assumption,” gangs that were socially connected to those targeted by the intervention were excluded from the analysis (Braga et al., 2014). Of the 19 treated gangs, 16 were successfully matched with an isolated comparison group. Hierarchical negative binomial growth curve regression models controlling for trends and seasonal variation were used to estimate the effects of the intervention on quarterly counts of total gang‐involved shootings, victim gang‐involved shootings, and suspect gang‐involved shootings (Braga et al., 2014). Trends in these offenses were analyzed from January 2006 through December 2010 using police incident data.

Standardized mean difference effect size statistics indicated that the intervention was associated with large effects for total gang‐involved shootings (*d* = −0.77) and suspect gang‐involved shootings (−0.87) relative to matched comparison gangs, but had a modest and nonsignificant effect on victim gang‐involved shootings (*d* = −0.48). Growth curve regression results suggested gangs targeted by the Ceasefire intervention experienced statistically significant (*p *< .05) reductions in total gang‐involved shootings (31%), suspect gang‐involved shootings (35%), and victim gang‐involved shootings (27%; Braga et al., 2014). Notably, seasonality was present for each of the three outcomes: gang‐involved shootings were significantly high in the spring (April through June) and summer (July through September) quarters compared to the winter quarter (January through March; Braga et al., 2014).

Because the number of gangs subjected to Ceasefire was staggered over the course of the intervention, Braga et al. (2014) performed supplementary analysis examining the timing of the treatment on offending patterns of gangs specifically targeted. In other words, they investigated whether the intervention produced structural breakpoints on quarterly total gang‐involved shootings. Negative binomial regression results revealed that “13 of the 16 matched treatment gangs experienced their largest statistically significant reduction in total shootings in the same quarter as or the quarter immediately following the full implementation of Ceasefire” (Braga et al., 2014, p. 134).

In an alternative assessment, Braga et al. (2013) focused on whether Operation Ceasefire II produced spillover effects on offending behavior of gangs vicariously associated with those directly targeted by the intervention. Propensity score analyses were used to match treated gangs, vicarious gangs, and untreated gangs. Treated gangs and vicarious gangs experienced significant (*p *< .05) reductions of 36 and 27%, respectively, in total gang‐involved shootings. Compared to matched untreated gangs, vicarious gangs experienced statistically significant (*p *< .05) reductions in quarterly counts of total gang‐involved shootings (24%) and suspect gang‐involved shootings (27%). Victim gang‐involved shootings were 19% less for vicarious gangs relative to their matched untreated comparison gangs, but the divergence was only marginally significant (*p *< .10; Braga et al., 2013). The authors conclude these results are evidence of a spillover effect and consistent with the general deterrence aim of “pulling levers” focused deterrence strategies (Braga et al., 2013).

### Community Initiative to Reduce Violence (CIRV) in Glasgow, Scotland

Faced with high levels of violence and a culture of weapon carrying among its youth, Glasgow implemented the CIRV. Modeled closely after Cincinnati's Initiative to Reduce Violence, Williams et al. (2014) described Glasgow's CIRV as a holistic focused deterrence public health approach aimed at reducing physical violence and weapon possession driven by gangs. While firearms were the focus in Cincinnati, cutting instruments and blunt objects were the weapons of interest in Glasgow (Williams et al., 2014).

The CIRV was a multiagency program headed by the Strathclyde Police. This program was active from October 24, 2008 to April 1, 2011 in two police divisions (BD and BA) that together represented Glasgow (Williams et al., 2014). Representatives of the CIRV invited gang‐involved youth to self‐referral sessions held at the Glasgow Sherriff Court where they met with a street worker who informed the attendee of the program and asked for the individual to sign a “no violence, no weapon” pledge (Williams et al., 2014). Additionally, a “needs analysis” was completed for the participant who was then connected to appropriate social services. Similar to other focused deterrence strategies, participants were informed the gang would be held collectively responsible and that one violation of the pledge by any member would result in the entire gang being temporarily excluded from the services provided by the CIRV (Williams et al., 2014).

A quasi‐experimental design with comparison groups matched on simple descriptives was used to estimate the effects of the intervention (Williams et al., 2014). The comparison group consisted of 250 males randomly selected from a pool of 431 known gang members in two neighboring divisions (GA and GE) where the CIRV was not operating. These two neighboring divisions were selected for comparison because they experienced a similar gang problem and socioeconomic characteristics to divisions receiving treatment. Each individual from the comparison group was matched based on age to an individual who was treated by the intervention (Williams et al., 2014). Conditional fixed effects Poisson regression models were used to evaluate changes in offending among both one and 2‐year treatment and control cohorts.

Physical violence decreased for both the 1‐year (*N *= 272) and 2‐year (*N *= 234) treatment cohorts 21% and 31%, respectively (Williams et al., 2014). The 1‐year intervention cohort (*N* = 204) experienced a 65% decline in weapon carrying postintervention, and the 2‐year intervention cohort (*N* = 286) experienced an 84% decline (Williams et al., 2014). Both of these reductions were significantly larger than declines by their control group counterparts (35% and 40%, respectively). Importantly, Williams et al. (2014) noted that other violence reduction initiatives, such as the Gang Task Force, were present throughout the region that may have influenced results.

### Group VRS in Chicago, IL

With a dual emphasis on focused deterrence and legitimacy, Chicago's Group VRS was designed to reduce gun violence citywide driven by gangs and gang factions (Papachristos & Kirk, 2015). Chicago's VRS predominantly drew on the deterrence doctrine exhibited by Boston's Operation Ceasefire and other “pulling levers” interventions. Although not entirely absent from “pulling levers” strategies in Boston and elsewhere, Chicago's VRS emphasized legitimacy and procedural justice throughout the intervention. Program coordinators deliberately chose places of significance to the community to host offender notification meetings and deliver their message. The enforcement message delivered to attendees at call‐ins centered on the increased enforcement and sanctions that would ensue if gun violence continues. Messages of changing norms and social services were also communicated, as community representatives plead with attendees to stop their violent behavior and opportunities were presented to participants for connecting with social services (Papachristos & Kirk, 2015). Chicago's VRS was first put into action in August 2010 and continued through 2013. During that time, 18 call‐ins were held and were attended by 438 individuals representing 149 gang factions (Papachristos & Kirk, 2015).

In recent years, the composition of gangs in Chicago has undergone a transformation. Historically, gangs in Chicago have followed a hierarchical structure (Venkatesh and Levitt 2000), but more recently gangs have been “splintering” into a large number of factions that are only loosely affiliated with a large gang (Papachristos & Kirk, 2015). Another difference in the recent landscape of gangs in Chicago compared to gangs historically is that intra‐gang violence is more prevalent than inter‐gang violence (Papachristos & Kirk, 2015).

Papachristos and Kirk (2015) evaluated Chicago's VRS using a quasi‐experimental design with matched comparison groups. Specifically, propensity score analyses were used to compare shooting trends among gang factions targeted by the intervention to shooting trends among matched comparison gangs (Papachristos & Kirk, 2015). Of the 149 gang factions that were represented at call‐ins, 148 were matched with at least one comparison group via propensity score analyses (Papachristos & Kirk, 2015). While the study period spans January 2006 through March 2014, outcome variables were evaluated for the 12 months after each gang faction was represented at a call‐in. The outcome of interest, the frequency of gang faction‐involved shootings, was measured in three ways: total shooting involvement, shooting victimization, and shooting offending (Papachristos & Kirk, 2015). In addition to the collective 12‐month follow‐up evaluation period, Cox proportional hazards models were used to examine the length of time after a call‐in that a gang faction was involved in a shooting.

Overall, gang factions experienced a marginally significant (*p *= .10, one‐tailed test) reduction of 23% in total shooting involvement compared to match comparison factions in the 12 months after a call‐in. Shooting victimization (fatal and nonfatal) among treated gang factions was significantly (*p *< .05) lower by 32% lower relative to matched comparison groups. No significant relationship was found between call‐in attendance and shooting offending by gang factions; however, Papachristos and Kirk (2015) noted the conclusiveness of this null finding was limited due to the relatively small sample size of known offending parties. Lastly, results from survival analyses indicated that treated gang factions had a significantly lower hazard of shooting involvement relative to their matched comparison (Papachristos & Kirk, 2015). In other words, more time elapsed for gang factions that attended a call‐in before being involved with a shooting compared to factions that did not attend the call‐in.

### Group VRS in New Orleans, LA

Funded by the New Orleans Community Support Foundation, the New Orleans Group Violence Reduction Strategy (GVRS) was implemented in response to serious violence driven disproportionately by gangs and groups throughout the city (Corsaro & Engel, 2015). With a history of scandals involving politicians and the police department, and a history plagued by chronic violence, Corsaro and Engel (2015) noted that New Orleans provided a unique context to test the effectiveness of focused deterrence strategies. The GVRS was coupled with the CURE Violence public health model to form one larger homicide reduction initiative called “NOLA for Life” (Skogan et al., 2008).

During the problem analysis of gangs in New Orleans, officers stressed the changing structure of gangs. Although gangs in the past tended to be hierarchical, intergenerational, and structured, recent years has seen gang membership and affiliation become looser and more fluid (Corsaro & Engel, 2015). Officers posited that displacement spurred by Hurricane Katrina may have been responsible for the disruption of traditional gang structure in New Orleans (Corsaro & Engel, 2015). Because of changes in the nature of gang structure, the GVRS targeted both gangs and the less restrictive classification of “groups.”

From October 2012 through March 2014, five offender notification meetings were held with a total of 158 individuals representing 54 gangs or groups (Corsaro & Engel, 2015). Attendees were informed of the enhanced enforcement and sanctions that would be imposed on the next group involved in a homicide or shooting. In addition to the enforcement message, social services were offered to attendees. The GVRS communication strategy also consisted of home visits, which were conducted with six individuals (Corsaro & Engel, 2015).

Assessment of the GVRS was completed in two stages. The first stage of analysis followed a quasi‐experimental design with nonequivalent control groups. Corsaro and Engel (2015) used difference‐in‐difference Poisson regression models to analyze changes in homicide trends in New Orleans following the implementation of the GVRS relative to 14 cities with persistently high homicide rates drawn from a pool identified by McCall, Land, and Parker (2011). In addition to the effects of the actual intervention, a placebo intervention period was also tested for comparing changes in homicide rates in New Orleans and comparable cities. Separately, group‐based trajectory analyses were conducted and identified six cities with high‐trajectory homicide trends for the 3 years immediately before GVRS was implemented (Corsaro & Engel, 2015). Difference‐in‐difference regression models were used to compare annual homicide trends in New Orleans to annual homicide rates in the six high trajectory cities for the first year that GVRS was implemented (2013), when GVRS was partially implemented (2012), and placebo year when no treatment was in place (2011).

The second stage of analysis used a quasi‐experimental time‐series design. Conditional negative binomial Poisson regression models controlling for trends and seasonal variations were used to estimate the longitudinal impact of the intervention. Complementary analysis examined the effects of the GVRS by race and age (Corsaro & Engel, 2015).

Comparing annual homicide rates in New Orleans to homicide rates in 14 cities with high homicide rates revealed a statistically significant decline in homicides that was unique to New Orleans for both four in which the GVRS was fully implemented (2013) and partially implemented (2012; Corsaro & Engel, 2015). Similarly, New Orleans experienced a statistically significant reduction in homicide rates when compared to the six immediate high trajectory cities in both 2013 and 2012 (Corsaro & Engel, 2015). For both comparisons, differences in homicide trends in New Orleans was distinct relative to nonequivalent comparison cities.

The second stage of the evaluation used monthly crime incident data from January 2010 through March 2014 to examine the effects of the GVRS on overall homicides, firearm‐related homicides, firearm assaults, and gang‐member‐involved (GMI) homicides, as well as overall property and overall violent crime for comparative purposes (Corsaro & Engel, 2015). Evidence suggested that the GVRS was related to a significant (*p *< .05) 17% decrease in monthly total homicide rates postintervention. Additionally, results indicated that the GVRS was associated with a significant (*p *< .01) 32% reduction in GMI homicide, while rates of non‐GMI homicides did not change significantly postintervention. Firearm‐related homicides and nonfatal firearm assaults both declined 16% significantly (*p *< .05) following implementation of the GVRS (Corsaro & Engel, 2015).

Corsaro and Engel (2015) completed supplemental analysis to investigate whether the intervention produced differential effects on the most “at‐risk” populations. Pre and postintervention homicide victimization trends were examined for four groups: black male victims ages 20–29, black male victims ages 30 and over, all other victims ages 20–29, and all other victims ages 30 and over. This analysis found that that GVRS was significantly related to only one of the four groups: homicide victimization of black males ages 20–29 was 27% lower in the postintervention period (Corsaro & Engel, 2015).

Lastly, to isolate the effects of the GVRS focused deterrence strategy from the CURE Violence public health program that was taking place simultaneously, Corsaro and Engel (2015) analyzed changes in total homicides and GMI homicides citywide while excluding the area where CURE Violence was active (Central City). Although Central City experienced declines postintervention for both total homicides and GMI homicides, those declines were nonsignificant whereas the declines in the remainder of the city were statistically significant (*p *< .05).

### No Violence Alliance in Kansas City, MO

After a change in political leadership in 2012, Kansas City's BJA‐funded Smart Policing Initiative (SPI) shifted its strategy of reducing violence from a foot patrol experiment to a focused deterrence “pulling levers” intervention that targeted group‐related violence (Novak, Fox, Carr, McHale, and White 2015). Specifically, the Kansas City No Violence Alliance (KC NoVA) sought to reduce violence citywide by focusing on chronically violent, group‐involved offenders (Fox et al., 2015). This initiative was led by an interagency Governing Board that included the county prosecutor, mayor, chief of police, probation, parole, FBI, ATF, U.S. District Attorney, and the Chancellor of the University of Missouri—Kansas City (Fox et al., 2015).

There were notable challenges encountered during the implementation of the KC NoVA, including a lack of directive to participating personnel, no clear decision‐making structure, and ineffective communication and coordination (Fox et al., 2015). These inefficiencies emerged throughout the first year of the intervention (2013), but were eventually corrected by the end of the year; consequently, full implementation was not achieved until the beginning of 2014 (Fox et al., 2015).

The intervention's message was disseminated to the targeted audience through home visits, police stations, probation and parole offices, and call‐in meetings. Targeted subjects were offered opportunities to utilize social services but were also informed of the increased enforcement they would encounter if they continue with their violent ways (Fox et al., 2015). In 2014, four call‐ins were conducted and 149 group‐involved individuals attended (Novak et al., 2015). Additionally, enforcement actions were taken against six groups (Novak et al., 2015).

Using police incident data, Fox et al. (2015) analyzed monthly counts of homicide and aggravated assault with a firearm from January 2010 through December 2014 to evaluate the effects of the KC NoVA. January 2014 was treated as the beginning of the postintervention period, and the intervention became increasingly implemented throughout the year. Interrupted time series models were used to compare violent crime rates before and after the intervention. The evaluation assessed crime outcomes at 1‐, 3‐, 6‐, and 12‐months after the intervention.

Results indicated that the KC NoVA was associated with significant and immediate reductions for both rates of homicide and aggravated assault with a firearm, but those effects diminished over time. The KC NoVA was associated with a statistically significant (*p *< .05) 40% reduction in homicide 1‐month postintervention, a significant 34% reduction after 3 months, and a significant 29% reduction after 6 months; however, the effects of the intervention were no longer significant 12‐months postintervention (Fox et al., 2015). Similarly, the KC NoVA was associated with significant reductions in gun‐involved aggravated assaults of 19% and 14% at one and 3‐month follow‐up periods, respectively. However, no significant relationship was observed at 6 and 12 months after the intervention, with the direction of the relationship becoming positive at 12 months (Fox et al., 2015). In short, the longer the intervention was in place, the less influential it became; Fox et al. (2015) posited this finding may be suggestive of a decay effect.

### Project Longevity in New Haven, CT

After experiencing rising trends in homicide and gun violence, New Haven became the pilot site for a statewide effort to curb gun violence (Sierra‐Arevalo et al., 2015). New Haven's focused deterrence strategy, Project Longevity, sought to reduce fatal and nonfatal shootings citywide by targeting gangs or groups involved in gun violence. Given that much of the violence stemmed from reciprocal conflict across groups, local law enforcement conducted a group audit and an incident review with an emphasis on ongoing intergroup violence (Sierra‐Arevalo et al., 2015).

Completion of the group audit led to the identification of 52 street unique groups that consisted of 440 identified street group members. However, only 22 of the 52 groups identified were involved in gun violence and, thus, targeted by the intervention. Intervention staff prioritized the two most violent street groups and invited both groups to the first call‐in, which was held in November 2012. At the call‐in, law enforcement, social service providers, and community members joined together to deliver to attendees a deterrence message consisting of consequences that will ensue if their offending continues, a moral plea to cease their offending, and an extension of services available to those seeking assistance. In total, six call‐ins were held between November 2012 and June 2014 with nearly all previously identified street groups participating in at least one meeting.

Monthly counts of fatal and nonfatal shootings were used from January 2011 through April 2014 to estimate the impact of the intervention. A series of ARIMA models were used to investigate compare pre‐ and postintervention trends in total shootings, group‐involved shootings, and nongroup‐involved shootings. In addition to the within city evaluation, Sierra‐Arevalo et al. (2015) incorporated three supplemental analyses to determine whether changes in crime trends were unique to New Haven's Project Longevity. First, shooting trends in New Haven were compared to shooting trends in a similarly situated city in the same state (Hartford). Second, to investigate whether the decline in crime was not a result of a decline in general crime, Sierra‐Arevalo et al. (2015) investigated pre and postintervention trends in offenses involving multiple offenders in New Haven. Third, the classification was for group‐involved shootings was expanded to include possible group‐involved shootings.

Regression results suggested that Project Longevity was associated with a statistically significant 37% reduction in total shootings and homicides citywide. Additionally, the intervention related to a statistically significant 73% decrease in group‐member‐involved homicides and shootings, which equates to approximately five fewer of such incidents per month. Supplemental analyses indicated that controlling for other parameters produces a slight reduction in the impact of the intervention but a statistically significant reduction in group‐involved still persists (Sierra‐Arevalo et al., 2015).

### DMI in Guntersville, AL

After traditional law enforcement tactics proved ineffective, Guntersville PD implemented a DMI in response to persistent drug and crime problems that predominately centered on an overt drug market operating at a specific community park. An interagency working group consisting of criminal justice entities, social services, and community representatives was formed to guide the intervention and overcome a history of strained relationships between local residents and law enforcement that included two highly publicized lawsuits pertaining to excessive use of force (Saunders et al., 2016).

The intervention was implanted in the neighborhood of Lakeview, with an emphasis on the community's park, where crack cocaine, marijuana, prescription pills, and methamphetamines were being sold. A call‐in was held in December 2011 and all six offenders selected to participate in the program attended the meeting. At the call‐in, attendees were informed of the details of the intervention, heard from concerned members of the community, and encouraged to utilize social services that were made available.

A quasi‐experimental design that incorporated synthetic control methods was used to evaluate the intervention. Negative binomial regression models controlling for trends were used to estimate the impact of the intervention on total crime, violent crime, property crime, and drug crime. Despite implementing the intervention as intended, regression results suggested there was no statistically significant relationship between the DMI and any of the four crime outcomes examined (Saunders et al., 2015).

### DMI in Montgomery County, MD

A city council member brought the DMI strategy to the attention of the police chief, who embraced the idea and took the lead implementing the strategy (Saunders et al., 2015). The DMI in Montgomery County was very narrow in its geographic scope by targeting a one‐block apartment complex called Damascus Gardens where all units were designated as Section 8 housing. Primarily, drugs being sold in the apartment complex were crack cocaine, heroin, prescription pills, and marijuana (Saunders et al., 2015). Local law enforcement, an assistant state's attorney, and county's Department of Health and Human Services were all central actors in implementing the intervention (Saunders et al., 2016). Further, law enforcement placed a confidential informant in the apartment complex who played a critical role in identifying eight A‐listers and nine B‐listers to include in the intervention.

All eight B‐listers who were selected to participate attended the call‐in. Attendees were told to stay “out of trouble with the law” for the 12 months following the call‐in or else the charged with the offenses police had already gathered (Saunders et al., 2016, p. 24). While social services were offered to attendees, only minimal attention was given to providing social services to clients (Saunders et al., 2016). Notably, team members noticed a considerable shift in the community's attitudes toward drug dealing following the call‐in, from complacent to optimistic that change can occur. Following the intervention, four law enforcement officers were dedicated to Damascus Gardens and the surrounding. They were able to police proactively in the targeted areas as they were relieved from responding to calls for service.

In terms of the intervention team's assessment, they believed the DMI successful shut down the overt drug market. Saunders et al. (2015) assessed the intervention quantitatively using a quasi‐experimental design that incorporated synthetic control methods to generate a matched comparison area. Negative binomial regression models controlling for trends were used to estimate the effects of the DMI at Damascus Gardens on total crime, violent crime, property crime, and drug crime. Regression results indicated that there were no statistically significant reductions in any of the outcome measures associated with the DMI (Saunders et al., 2015).

### DMI in Roanoke, VA

Successes associated with the DMI in High Point, North Carolina inspired Roanoke's police chief to lead the formation of an interagency task for to implement a DMI in Roanoke (Saunders et al., 2016). In addition to reducing crime, the task force saw the DMI strategy as an opportunity to improve the community's perception of police.

The Roanoke DMI completed two call‐ins: the first in December 2011 in the Hurt Park neighborhood and the second in January 2013 in the Melrose‐Rugby neighborhood. The original target of Roanoke's DMI was the Hurt Park neighborhood, where the drugs most commonly sold were crack cocaine and marijuana (Saunders et al., 2015). Although Hurt Park had consistently high crime rates, it also had a strong community organizing presence who supported the DMI.

Intelligence gathering led to 10 offenders being designated as A‐listers and five as B‐listers (Saunders et al., 2016). At the call‐in meeting, attendees were told to refrain from criminal activity for the ensuing 6 months or else they would face charges for their previously observed crimes. Further, law enforcement, social services, and community leaders were all given a chance to speak at the call‐in meetings and express their desire for attendees to change their criminal behavior (Saunders et al., 2016). For the second call‐in, 15 A‐listers were identified and five B‐listers participated in the intervention.

A quasi‐experimental design with a matched comparison generated through synthetic control methods was used to evaluate the impact of this intervention. Negative binomial regression models controlling for trends were used to estimate the impact of the DMI on crime. For Hurt Park, regression results suggested that the DMI was associated with a statistically significant 30% reduction in total crime 3‐month postintervention, as well as a 19% reduction at 6 months, 28% reduction at 9 months, and 23% reduction at 12 months. Further, Hurt Park experienced a significant 45% decline in property rime at 6 months, 57% decline at 9 months, and 50% decline at 12‐month postintervention. There was also a significant decrease in violent crime of 24% at 3 months and 29% at 9 months following the intervention. No significant relationship was found between the DMI and drug crime in Hurt Park. For the Melrose‐Rugby neighborhood, violent crime was the only outcome significantly related to the DMI with reductions of 15% at 3‐month and 34% at 6‐month postintervention. There was no significant relationship discovered between the DMI in Melrose‐Rugby and total crime, property crime, or drug crime.

### DMI in Seattle, WA

There were two areas targeted by the DMI in Seattle: 23rd Street Corridor in the fall of 2009 and the International District in 2013 (Saunders et al., 2015). The first intervention site was selected because drug dealing concentrated predominately in two areas along the 23rd Street Corridor, which also had a history of gang violence. Undercover drug buys and video surveillance led to the identification of 20 DMI candidates. One challenge that emerged for the first call‐in pertained to the inability of members of Seattle PD on the intervention team to discuss the project with patrol and other personnel, which Saunders and colleagues speculated may have inhibit law enforcement's commitment to the program. This DMI also had to overcome negative community relations that were left behind by a Department of Justice “Weed and Seed” program that previously operated in the 23rd Street Corridor. The community did not embrace the previous program and it left some members of the community with disenfranchised and antipolice sentiments.

Seattle implemented a DMI in the International District in 2013. A total of 12 individuals were identified as DMI candidates through investigative strategies. There were a number of challenges faced during the implementation stage: confusing between the DMI and a similar program called Law Enforcement Assisted Diversion, language barriers between the task force and the predominately Asian speaking community, and most targeted offenders lived outside of the target area resulting in less buy‐in from the candidates and the community. Notably, a third DMI was held in early 2011 and focused on an area in Southeast Seattle; however, during the implementation of this iteration there was a change in command at Seattle PD couple with a change in the department's priorities.

Community mobilization efforts before the call‐ins helped garner community support for the initiatives. In addition to law enforcement's message of deterrence, community members offered support and disapproval of continued drug dealing and social services were offered to attendees. After the call‐in, uniformed patrols increased and calls for service were prioritized in the targeted areas. Six months after the call‐in in the 23rd Street Corridor, drug dealing began increasing and SPD took enforcement actions against 17 drug dealers in the target area. The intervention team continued efforts to maintain positive relationships with the target communities after the call‐ins by hosting community cleanups and barbecues.

To estimate the impact of the intervention, Saunders et al. (2015) used a quasi‐experimental design with a matched comparison area created through synthetic control techniques. A series of negative binomial regression models were used to investigate the effects of the DMI across four outcome measures: total crime, violent crime, property crime, and drug crime.

Regression results indicated that the DMI in the International District was related to a statistically significant 15% reduction in total crime at 3‐ and 6‐month postintervention. Three months after the intervention, property crime was significantly 8% lower, violent crime was significantly 53% lower, and drug crime was significantly 29% lower (Saunders et al., 2015). In contrast to the International District, which saw significant reductions across each of the four outcome measures, the intervention in the 23rd Street Corridor was not significantly associated with reductions in any of four crime types.

### DMI in Ocala, FL

Initiated by the Chief of Police of the Ocala Police Department (OPD), the intervention team consisted of the OPD, the city's drug task force, an assistant state's attorney, a community activist, and social service providers. Initially, the state's attorney office resisted involvement in the DMI because of a perception that it was “soft on crime”; however, efforts to convince the state's attorney's office, led by OPD's Chief, of the DMI's merit were successful and resulted in their full commitment to the intervention. Two sites were selected as target areas for the DMI: the “Second Chance” neighborhood in November 2009 and the First Avenue housing project in the October 2010 (Saunders et al., 2015). Problem analysis and investigative efforts led to the identification of 13 candidates and 15 candidates for the DMIs in “Second Chance” and First Avenue target areas, respectively. Those who were not chronic or violent offenders were invited to the call‐in, where they were presented the evidence already collected against them, offered an opportunity to avoid being arrested by ceasing to dealing drugs, and were offered social services.

Prior to the call‐ins, the DMI team attempted to garner support and build positive relationships with the community by hosting barbecues, special events, and town hall meetings. At the town hall meetings, the sergeant helped put to rest community concerns that the DMI was targeting young black men. Following the call‐ins, police prioritized calls for service pertaining to drug complaints and increased their presence in each of the target neighborhoods. The DMI team continued to host events for the community and conduct cleanups, and social services follow‐up with call‐in attendees.

A quasi‐experimental design with matched comparisons via synthetic control methods was used to evaluate the impact of the DMI. Negative binomial regression models were used to assess whether the intervention was associated with reductions in total crime, violent crime, property crime, and drug crime. Across all regression models, no significant relationship was found between the DMI and any of the four outcome measures. Despite the null impact on the outcome measures, the DMI team felt the intervention strongly contributed to the development of positive police‐community relations.

## APPENDIX D STUDIES NOT FITTING SELECTION CRITERIA

During our systematic search of the literature, we identified a number of focused deterrence interventions that did not fit our inclusion criteria. Focused deterrence interventions that did not meet our eligibility standards due, broadly, for one of two reasons: (a) full program implementation was not achieved; and (b) key components of the traditional “pulling levers” focused deterrence approach were absent in the intervention. In this section, we detail studies of focused deterrence strategies that fell short of being eligible for our meta‐analysis.

### Implementation

#### Selected Studies from the RAND Drug Market Intervention Cohort

The Bureau of Justice Statistics funded a cohort of seven sites to test whether the effects of the drug market intervention model used in High Point, North Carolina in different settings (Saunders et al., 2015). Sites included in this DMI cohort were: Guntersville, AL; Jacksonville, FL; Gary, IN; Montgomery County, MD; New Orleans, LA; and Roanoke, VA. An assessment of program implementation fidelity completed by Saunders et al. (2015) identified two of these sites (Gary, IN and Jacksonville, FL) that failed to complete the first phase of the implementation. To have a more comprehensive outcome assessment, the Gary and Jacksonville DMIs were replaced in the cohort evaluation with two sites from a previous DMI cohort (Seattle, WA and Ocala, FL).

We elected to include only sites from this DMI cohort evaluation that completed each stage of the implementation process with at least medium or high fidelity, as rated by Saunders et al. (2015). Sites that achieved this standard, and therefore were included in our meta‐analysis, were: Guntersville, Alabama; Montgomery County, Maryland; Roanoke, Virginia; Seattle, Washington; and Ocala, Florida. The remaining sites in the DMI cohort that were excluded from our meta‐analysis for incomplete or poor program implementation included Flint, MI; Gary, IN; and Jacksonville, FL.

Notably, of the sites from the RAND DMI cohort that were excluded from our meta‐analysis, Flint was the only one that conducted a call‐in with identified offenders (Saunders et al., 2015). Unlike most focused deterrence strategies that originate from police departments or public officials, Flint's DMI spawned from the efforts of a local community organization (the Flint Area Congregation Together) who brought the idea to the mayor and city council (Saunders et al., 2016). The Flint DMI was a multiagency initiative that consisted of representatives from 21 organizations ranging from law enforcement and government departments to social services and community organizations. Originally called Flint Ceasefire, the name “Flint Lifelines” was later adopted.

The initial announcement of the DMI was met with skepticism by the media and community due to failures of previous crime reduction strategies. Feeling pressure to implement the DMI quickly, the initiative was launched and a call‐in was scheduled prior to the task force receiving any formal training and before the exact composition and details of the intervention were determined. However, after several delays and attending the DMI training session held by Michigan State University, the intervention team realized they needed to take additional measures before hosting a call‐in. Ultimately, the first call‐in was held in July 2011 nearly 18 months after the original call‐in was scheduled. This considerable delay between the announcement of the program and actually enactment further fueled the media's and community's skepticism of the intervention.

The DMI was implement in the city's Second Ward, where vacant housing units were used to sell cocaine and heroin (Saunders et al., 2015). In addition to having one of the most active drug markets in Flint, the target area was also selected for having a disproportionately high concentration of Part I index crimes (Saunders et al., 2016). Despite challenges in the intelligence gathering process, the intervention team was able to identify 20 offenders to use as examples of increased punishment and seven offenders to participate in a call‐in, six of whom attended; later, in 2013, two additional call‐ins were held (Saunders et al., 2016).

Saunders and colleagues (2015) completed an outcome evaluation that utilized synthetic control models in a quasi‐experimental design to compare crime trends in the targeted neighborhood to trends in the matched comparison area. Negative binomial regression models controlling for crime trends were used to estimate the impact of the intervention on the time‐series. Results indicated that the intervention was not significantly related to any of the outcome measures assessed (total crime, violent crime, property crime, and drug crime).

There were three major shortcomings in the implementation of the Flint DMI that led to its exclusion from the present meta‐analysis. First, the problem analysis stage, specifically intelligence gathering, was hampered due the limited number of officers devoted to the intervention, lack of feedback from the community, and unreliable undercover informants. Second, due to strained and limited resources, no enforcement actions were carried out after the call‐in and crime problems in the target area could not be prioritized (Saunders et al., 2016). Third, quick bail and a refusal by judges to give arrested offenders harsher sentences undermined the deterrent message of the intervention.

### Intervention Components

#### Project Hawaii Opportunity with Probation Enforcement (HOPE) in Honolulu, Hawaii

HOPE intervention was a community supervision program aimed at substance‐abusing probationers (Hawken & Kleiman, 2009). The program relied on a mandate to abstain from illicit drugs, backed by swift and certain sanctions for drug test failures, and preceded by a clear and direct warning. Probationers were sentenced to drug treatment only if they continued to test positive for drug use, or if they requested a treatment referral. The deterrence‐based HOPE intervention differs significantly from typical drug court operations as it economizes on treatment and court resources. As Hawken and Kleiman (2009) suggest, HOPE does not mandate formal treatment for every probationer, and does not require regularly scheduled meetings with a judge; probationers appear before a judge only when they have violated a rule. HOPE is often linked to the DMI approaches as a related application of focused deterrence (see, e.g., Boyum, Caulkins, & Kleiman, 2011) as well as gang and group‐based pulling levers focused deterrence based on the common strategy of certain punishment for offenders (Durlauf & Nagin, 2011).

The HOPE evaluation used a randomized controlled trial among general‐population substance‐abusing probationers where probationers assigned to treatment conditions were compared to probationers assigned to probation‐as‐usual control conditions (Hawken & Kleiman, 2009). In their unpublished report to the U.S. National Institute of Justice, Hawken and Kleiman (2009) state that that HOPE relies on a mandate to abstain from illicit drugs, backed by swift and certain sanctions and preceded by a clear and direct warning. Unlike most diversion programs and drug courts, it does not attempt to impose drug treatment on every participant. Under HOPE, probationers are sentenced to drug treatment only if they continue to test positive for drug use, or if they request a treatment referral. According to Hawken and Kleiman (2009), HOPE should be considered to be distinct from drug courts in economizing on treatment and court resources (probationers appear before a judge only when a violation is detected). HOPE's stated goals are reductions in drug use, new crimes, and incarceration.

The randomized controlled trial used an intent‐to‐treat design where all offenders randomly allocated to the treatment condition were included in the HOPE group whether they formally entered the program or not. Of the eligible probationers, two thirds were assigned to the HOPE treatment (*n* = 330) and one‐third were assigned to the control group (*n* = 163). Ninety‐three percent of the probationers assigned for treatment appeared for their initial HOPE warning hearing and participated in the intervention. The experiment commenced in October 2007 and the intervention period lasted for 1 year.

Based on their analyses of the experimental data, Hawken and Kleiman (2009) concluded that HOPE was very effective in changing the behaviors of substance‐abusing probationers. Only 21% of HOPE probationers experienced new arrests as compared to 47% of control probationers (*p* < 01). HOPE probationers outperformed control probationers on a number of other performance measures such as missed probation appointments (treatment = 9%, control = 23%), positive urine drug test results (treatment = 13%, control = 46%), revocation rates (treatment = 7%, control = 15%), and the number of days sentenced to incarceration (treatment = 138 days, control = 267 days).

Based on our selection criteria, HOPE was not included in our final review. However, as stated earlier, several scholars believed that HOPE does fit within the general framework of pulling levers focused deterrence strategies. We agree that it is broadly similar to another evaluation included in our systematic review that is focused on a corrections population—Chicago's PSN intervention (Papachristos et al., 2007). The key elements of Chicago PSN strategy are administered by the Illinois Department of Correction and the U.S. Attorney's Office (the call‐in session is given to returning parolees to selected neighborhoods). The contribution of the Chicago Police Department is limited to increasing their gun policing efforts in the selected neighborhoods. The CPD does not select the returning parolees for the intervention nor do they run the communications strategy. Their only role is to increase gun recoveries and arrest those who commit violent crimes in these neighborhoods.

Moreover, probation has a central role in all of the gang/group‐based focused deterrence interventions included in our review. Monitoring offenders in the community to ensure they are abiding by probation conditions, changing conditions, and revoking probation are key levers that are pulled in the application of focused deterrence strategies to gangs and criminally active groups. In interagency working group settings, all involved agencies govern the shape and content of the pulling levers interventions. While the police convence the working group meeting, they share governance with the other criminal justice agencies, social service providers, and community members in the group. Probation is involved as a key decision maker in the process.

Most applications of pulling levers focused deterrence strategies have therapeutic elements (e.g., Braga et al., 2001; Papachristos et al., 2007). Indeed, the working group has social service providers, street outreach workers, and community members as core members. A vital part of the communications strategy to pair threats of sanctions with offers of help (job training/placement, education, substance abuse counseling, etc.). All targeted gangs and groups are offered services throughout the entire process. Under HOPE, probationers are sentenced to drug treatment only if they continue to test positive for drug use, or if they request a treatment referral. HOPE is distinct from drug courts in economizing on treatment and court resources (probationers appear before a judge only when a violation is detected).

#### Project CeaseFire in South Carolina

Project CeaseFire in South Carolina was a multiagency effort headed by the U.S. Attorney's Office for the District of South Carolina and consisted of South Carolina's Department of Public Safety, the South Carolina Department of Probation, Parole, and Pardon Services (SCDPPPS), the South Carolina Law Enforcement Division, and the Bureau of Alcohol, Tobacco, and Firearms (Barnes et al., 2010). Several programs were encompassed by this broader initiative, including “public service announcements, firearms task forces, ballistic laboratory enhancement, firearm violence investigators, anti‐gang investigators, gang awareness programs, and community gun crime prevention programs” (p. 384). Although this slew of programs all fell under the realm of Project CeaseFire, the evaluation conducted by Barnes et al. (2010) exclusively assessed the deterrence‐based notifications carried out by the SCDPPPS.

SCDPPPS sought to increase perceptions of the certainty and severity of punishments among convicted offenders actively on probation and parole (Barnes et al., 2010). After receiving specialized training, probation and parole officers notified all new probationers and parolees at intake of the firearm statutes and how they would be enforced against those who were under supervision. Prior to January 2005, all parolees were informed of federal firearms statutes but Project CeaseFire extended this notification process to all subjects of community supervision.

The evaluation of the SCDDPPS component of Project Ceasefire utilized a quasi‐experimental design. A sample of 400 probationers and parolees was used to evaluate the program: 200 subjects were randomly selected from a pool of offenders between March 2004 and May 2004 to form the comparison group and the treatment group consisted of 200 offenders under supervision in the same months the following year. Gun‐related crimes for 18‐months following the subject's admission to community supervision. Controlling for a number of individual‐level sociodemographic factors, logistic regression results suggested a positive relationship between being exposed to the notification of firearms statutes and subsequent involvement in illegal gun activity. Standard logistic regression results indicated this effect was statistically significant; however, although the direction of the relationship was maintained, rare‐events logistic regression model did not produce statistically significant results indicating the relationship is sensitive to model specification. Barnes et al. (2010) also performed a survival analysis to assess the effectiveness of the program but were limited by relatively few cases of failure. Results from the Cox regression analysis suggested that the treatment group were quicker to commit a gun‐related offense at a marginally significant rate (*p* < .10) than the comparison group.

The authors urged caution when interpreting the findings given the sensitivity of results due to model specification. They hypothesized the increase in gun‐related offenses for the treatment group may have been the result of heightened awareness of firearms statutes among law enforcement entities potentially leading to a systematic change in reporting practices. Also, the authors note the collaboration between SCDPPPS and ATF may have increased reporting of firearms violations because of the federal assistance in prosecuting gun crimes.

We excluded this study from out meta‐analysis for a variety of reasons. This evaluation focused exclusively on the impact of the notifications component of the larger CeaseFire intervention. How the notifications were carried out was not in accordance with procedures used in traditional “pulling levers” strategies. First, notably absent from this portion of the intervention were representatives from the community and social service providers. Relatedly, the authors note the vast range of agencies involved in the comprehensive CeaseFire initiative; however, this evaluation exclusively examines the impact of the notifications component that was conducted by SCDPPPS and appears to lack the interagency collaboration commonly found in the traditional focused deterrence model. Another reason this study was not deemed eligible was due to how notifications were carried out. These notifications were not focused at any specific individuals, groups, or areas. Instead, the notification was presented to a broad population (e.g., all new probationers and parolees). Lastly, this notification program appears to lack enforcement action that is critical to focused deterrence strategies. Although law enforcement agencies were part of the larger intervention, their participation in the program evaluated in this study was noticeably absent.

#### PSN in the Eastern District of Missouri

In 2002, PSN was implemented in the Eastern District of Missouri. The primary focus of this iteration of PSN was the high rates of homicide and gun violence in the city of St. Louis (Decker et al., 2007). Coordinated by the U.S. Attorney's Office, a task force was formed that included local, state, and federal law enforcement agencies; local and federal prosecutors; probation and parole offices; juvenile court; a hospital; city neighborhood services; street outreach workers; media partners; the regional justice information system; and researchers from the University of Missouri, St. Louis. This task force utilized a variety of strategies to address high levels of gun crime, including targeted enforcement, intensive gun case prosecution review, a most violent offender program, and notification meetings for high‐risk probationers (Decker et al., 2007).

Decker et al. (2007) utilized a quasi‐experimental design to estimate the impact of the PSN intervention on neighborhood crime rates. Crime rates in 14 treatment neighborhoods were compared to two different comparison groups. The first comparison group consisted of control neighborhoods that were selected based on similarity of sociodemographic characteristics and crime rates. In order to detect displacement and diffusion, a second comparison group consisting of neighborhoods contiguous to target neighborhoods was used.

Results from the outcome assessment were supportive of strong program fidelity. Specifically, the number of federal prosecutions and offenders convicted for gun crimes at the state and federal levels both increased significantly. Less clear, however, is the intervention's impact on crime rates. Violent gun crime declined significantly in targeted neighborhoods, but it also declined significantly citywide. Furthermore, the decrease in gun crime arrests in the treatment neighborhoods was not significantly different from arrest rates observed in control and contiguous neighborhoods (Decker et al., 2007).

Although PSN initiatives typically adhere to principles of “pulling levers” focused deterrence, we conclude that this particular intervention employs a collection of tactics that build on certain elements of focused deterrence but fails to implement a comprehensive focused deterrence strategy (see Kennedy, 2006). For example, the gun case prosecution review component of this intervention was largely a reform of court and prosecution practices rather than an application of deterrence. Additionally, the targeted enforcement strategies and most violent offenders program as described in the evaluation appear strictly reactive rather than proactive, and omit the communication component of the traditional “pulling levers” approach. Lastly, the probation notification portion of this PSN intervention appears to be a stand‐alone program without any firm criteria offered about why probationers were selected. It is also unclear whether this notification process was accompanied by any enhanced attention and enforcement.

## APPENDIX E ADDITIONAL REFERENCES FOR APPENDICES

Barnes, J. C., Kurlychek, M. C., Miller, H. V., Miller, J. M., & Kaminski, R. J. (2010). A partial assessment of South Carolina's Project Safe Neighborhoods strategy: Evidence from a sample of supervised offenders. *Journal of Criminal Justice*, *38*(4), 383–389.

Berk, R. A. (2005). Knowing when to fold ‘em: An essay on evaluating the impact of Ceasefire, Compstat, and Exile. *Criminology and Public Policy*, *4*(3), 451–465.

Boyum, D. A., Caulkins, J. P., & Kleiman, M. A. (2011). Drugs, crime, and public policy. In J. Q. Wilson & J. Petersilia (Eds.), *Crime and public policy* (pp. 368–410). New York, NY: Oxford University Press.

Braga, A. A., McDevitt, J., & Pierce, G. L. (2006). Understanding and preventing gang violence: Problem analysis and response development in Lowell, Massachusetts. *Police Quarterly, 9* (1), 20–46.

Decker, S. H., Huebner, B. M., Watkins, A., Green, L., Bynum, T., & McGarrell, E.F. (2007). *Project safe neighborhoods: Strategic interventions. Eastern district of Missouri: Case study 7*. Washington, DC: U.S. Department of Justice.

Engel, R. S., Baker, S. G., Tillyer, M. S., Eck, J., & Dunham, J. (2008). *Implementation of the Cincinnati initiative to reduce violence (CIRV): Year 1 report*. Cincinnati, OH: University of Cincinnati Policing Institute.

Engel, R. S., Tillyer, M. S., & Corsaro, N. (2013). Reducing gang violence using focused deterrence: Evaluating the Cincinnati Initiative to Reduce Violence (CIRV). *Justice Quarterly*, *30*(3), 403–439.

Engel, R. S., Tillyer, M. S., Dunham, J., Hall, D., Ozer, M., Henson, B., & Godsey, T. (2009). *Implementation of the Cincinnati initiative to reduce violence (CIRV): Year 2 report*. Cincinnati, OH: University of Cincinnati Policing Institute.

Hawken, A., & Kleiman, M. A. (2009). *Managing drug involved probationers with swift and certain sanctions: Evaluating Hawaii's HOPE*. Final report submitted to the National Institute of Justice, U.S. Department of Justice.

Kennedy, D. M. (1997). Pulling levers: Chronic offenders, high‐crime settings, and a theory of prevention. *Valparaiso University Law Review*, *31*(2), 449–484.

McCall, P. L., Land, K. C., & Parker, K. F. (2011). Heterogeneity in the rise and decline of city‐level homicide rates, 1976–2005: A latent trajectory analysis. *Social Science Research*, *40*(1), 363–378.

McGarrell, E. F., & Chermak, S. (2003). *Strategic approaches to reducing firearms violence: Final report on the Indianapolis violence reduction partnership*. Washington, DC: National Institute of Justice.

Meares, T., Papachristos, A. V., & Fagan, J. (2009). *Homicide and gun violence in Chicago: Evaluation and summary of the Project Safe Neighborhoods Program*. Retrieved from https://www.flintridge.org/newsresources/documents/HomicideandGunViolenceinChicago‐EvaluationandSummaryoftheProjectSafeNeighborhoodsProgram‐2009.pdf

Novak, K. J., Fox, A. M., Carr, C. M., McHale, J., & White, M. D. (2015). *Kansas City, Missouri smart policing initiative: From foot patrol to focused deterrence*. Smart Policing Initiative Spotlight Report. Washington, DC: Bureau of Justice Assistance.

Piehl, A. M., Kennedy, D. M., & Braga, A. A. (2000). Problem solving and youth violence: An evaluation of the Boston Gun Project. *American Law and Economics Review*, *2*(1), 58–106.

Skogan, W. G., Hartnett, S. M., Bump, N., & Dubois, J. (2008). *Evaluation of CeaseFire‐Chicago*. Washington, DC: U.S. Department of Justice.

Tita, G., Riley, K. J., & Greenwood, P. (2003). From Boston to Boyle heights: The process and prospects of a “pulling levers” strategy in a Los Angeles barrio. In S. H. Decker (Ed.), *Policing gangs and youth violence* (pp. 102–130). Belmont, CA: Wadsworth.

Venkatesh, S. A., & Levitt, S. D. (2000). “Are we a family or a business?” History and disjuncture in the urban American street gang. *Theory and Society*, *29*(4), 427–462.

Wakeling, S. (2003). *Ending gang homicide: Deterrence can work* (Perspectives on Violence Prevention No. 1). Sacramento, CA: California Attorney General's Office/California Health and Human Services Agency.

## References

[cl21051-bib-0001] Boyle, D. J. , Lanterman, J. L. , Pascarella, J. E. , & Cheng, C. C. (2010). The impact of Newark's operation ceasefire on trauma center gunshot wound admissions. Justice Research and Policy, 12(2), 105–123.

[cl21051-bib-0002] Braga, A. A. (2008b). Pulling levers focused deterrence strategies and the prevention of gun homicide. Journal of Criminal Justice, 36(4), 332–343.

[cl21051-bib-0003] Braga, A. A. , Hureau, D. M. , & Papachristos, A. V. (2014). Deterring gang‐involved gun violence: Measuring the impact of Boston's operation ceasefire on street gang behavior. Journal of Quantitative Criminology, 30(1), 113–139.

[cl21051-bib-0004] Braga, A. A. , Kennedy, D. M. , Waring, E. J. , & Piehl, A. M. (2001). Problem‐oriented policing, deterrence, and youth violence: An evaluation of Boston's operation ceasefire. Journal of Research in Crime and Delinquency, 38(3), 195–225.

[cl21051-bib-0005] Braga, A. A. , Pierce, G. L. , McDevitt, J. , Bond, B. J. , & Cronin, S. (2008). The strategic prevention of gun violence among gang‐involved offenders. Justice Quarterly, 25(1), 132–162.

[cl21051-bib-0006] Corsaro, N. , & Brunson, R. K. (2013). Are suppression and deterrence mechanisms Enough? Examining the “pulling levers” drug market intervention strategy in Peoria, Illinois, USA. International Journal of Drug Policy, 24(2), 115–121. 23352584 10.1016/j.drugpo.2012.12.006

[cl21051-bib-0007] Corsaro, N. , Brunson, R. K. , & McGarrell, E. F. (2009). Problem‐oriented policing and open‐air drug markets: Examining the rockford pulling levers deterrence strategy. Crime & Delinquency, 59(7), 1085–1107.

[cl21051-bib-0008] Corsaro, N. , & Engel, R. S. (2015). Most challenging of contexts: Assessing the impact of focused deterrence on serious violence in New Orleans. Criminology & Public Policy, 14(3), 471–505.

[cl21051-bib-0009] Corsaro, N. , Hunt, E. D. , Hipple, N. K. , & McGarrell, E. F. (2012). The impact of drug market pulling levers policing on neighborhood violence: An evaluation of the high point drug market intervention. Criminology and Public Policy, 11(2), 167–199.

[cl21051-bib-0010] Corsaro, N. , & McGarrell, E. F. (2009). An evaluation of the Nashville drug market initiative (DMI) pulling levers strategy. East Lansing, MI: Michigan State University, School of Criminal Justice.

[cl21051-bib-0011] Delaney, C. (2006). The effects of focused deterrence on gang homicide: An evaluation of Rochester's Ceasefire program. Rochester, NY: Rochester Institute of Technology.

[cl21051-bib-0012] Engel, R. S. , Corsaro, N. , & Tillyer, M. S. (2010). Evaluation of the Cincinnati initiative to reduce violence (CIRV). Cincinnati, OH: University of Cincinnati Policing Institute.

[cl21051-bib-0013] Farrington, D. P. (2006). Methodological quality and the evaluation of anti‐crime programs. Journal of Experimental Criminology, 2(3), 329–337.

[cl21051-bib-0014] Fox, A. M. , Novak, K. J. , & Yaghoub, M. B. (2015). Measuring the impact of Kansas city's no violence alliance. Kansas City, MO: Department of Criminal Justice and Criminology, University of Missouri—Kansas City.

[cl21051-bib-0015] McGarrell, E. F. , Chermak, S. , Wilson, J. M. , & Corsaro, N. (2006). Reducing homicide through a “lever‐pulling” strategy. Justice Quarterly, 23(2), 214–231.

[cl21051-bib-0016] Papachristos, A. V. , & Kirk, D. S. (2015). Changing the street dynamic: Evaluating Chicago's group violence reduction strategy. Criminology & Public Policy, 14(3), 525–558.

[cl21051-bib-0017] Papachristos, A. V. , Meares, T. L. , & Fagan, J. (2007). Attention felons: Evaluating project safe neighborhoods in Chicago. Journal of Empirical Legal Studies, 4(2), 223–272.

[cl21051-bib-0018] Saunders, J. , Kilmer, B. , & Ober, A. (2015). A comprehensive evaluation of a drug market intervention training cohort. Santa Monica, CA: RAND Corporation.

[cl21051-bib-0019] Sierra‐Arevalo, M. , Charette, Y. , & Papachristos, A. V. (2015). Evaluating the effect of project longevity on group‐involved shootings and homicides in New Haven, CT. New Haven, CT: Yale University.

[cl21051-bib-0020] Tita, G. , Riley, K. J. , Ridgeway, G. , Grammich, C. , Abrahamse, A. F. , & Greenwood, P. W. (2004). Reducing gun violence: Results from an intervention in East Los Angeles. Santa Monica, CA: RAND Corporation.

[cl21051-bib-0021] Williams, D. J. , Currie, D. , Linden, W. , & Donnelly, P. D. (2014). Addressing gang‐related violence in Glasgow: A preliminary pragmatic quasi‐experimental evaluation of the community initiative to reduce violence (CIRV). Aggression and Violent Behavior, 19(6), 686–691.

[cl21051-bib-0022] Andrews, D. A. , & Bonta, James (2006). The psychology of criminal conduct (4th ed.). Newark, NJ: LexisNexis/Matthew Bender.

[cl21051-bib-0023] Apel, R. , & Nagin, D. S. (2011). General deterrence: A review of recent evidence. In J. Q. Wilson , & J. Petersilia (Eds.), Crime and public policy (pp. 411–436). New York, NY: Oxford University Press.

[cl21051-bib-0024] Bowers, K. , Johnson, S. , Guerette, R. T. , Summers, L. , & Poynton, S. (2011). Spatial displacement and diffusion of benefits among geographically focused policing initiatives. Campbell Systematic Reviews, 7, 1–144.

[cl21051-bib-0025] Braga, A. A. (2008a). Problem‐oriented policing and crime prevention (2nd ed.). Boulder, CO: Lynne Rienner Publishers.

[cl21051-bib-0026] Braga, A. A. (2012). Getting deterrence right?: Evaluation evidence and complementary crime control mechanisms. Criminology and Public Policy, 11(2), 201–210.

[cl21051-bib-0027] Braga, A. A. , Apel, R. , & Welsh, B. C. (2013). The spillover effects of focused deterrence on gang violence. Evaluation Review, 37(3–4), 314–342. 24569771 10.1177/0193841X13518535

[cl21051-bib-0028] Braga, A. A. , & Kennedy, D. M. (2012). Linking situational crime prevention and focused deterrence strategies. In N. Tilley , & G. Farrell (Eds.), The reasoning criminologist: Essays in honour of Ronald V. Clarke (pp. 65–79). London: Taylor and Francis.

[cl21051-bib-0029] Braga, A. A. , Papachristos, A. V. , & Hureau, D. M. (2014). The effects of hot spots policing on crime: An updated systematic review and meta‐analysis. Justice Quarterly, 31(4), 633–663.

[cl21051-bib-0030] Braga, A. A. , & Weisburd, D. L. (2012). The effects of “pulling levers” focused deterrence strategies on crime. Campbell Systematic Reviews, 8, 1–90. 10.1002/cl2.1051 PMC8356499 37133287

[cl21051-bib-0031] Braga, A. A. , & Weisburd, D. L. (2011). The effects of focused deterrence strategies on crime: A systematic review and meta‐analysis of the empirical evidence. Journal of Research in Crime and Delinquency, 49(3), 323–358.

[cl21051-bib-0032] Braga, A. A. , & Weisburd, D. L. (2014). Must we settle for less rigorous evaluations in large area‐based crime prevention programs? Lessons from a Campbell review of focused deterrence. Journal of Experimental Criminology, 10(4), 573–597.

[cl21051-bib-0033] Braga, A. A. , & Winship, C. (2006). Partnership, accountability, and innovation: Clarifying Boston's experience with pulling levers. In D. Weisburd , & A. A. Braga (Eds.), Police innovation: Contrasting perspectives (pp. 171–188). New York, NY: Cambridge University Press.

[cl21051-bib-0034] Brunson, R. K. (2015). Focused deterrence and improved police‐community relations: Unpacking the proverbial “black box”. Criminology & Public Policy, 14(3), 507–514.

[cl21051-bib-0035] Campbell, D. T. , & Boruch, R. F. (1975). Making the case for randomized assignment to treatment by considering the alternatives. In C. A. Bennett , & A. A. Lumsdaine (Eds.), Evaluation and experiments: Some critical issues in assessing social programs (pp. 195–296). New York, NY: Academic Press.

[cl21051-bib-0036] Campbell, D. T. , & Stanley, J. C. (1966). Experimental and quasi‐experimental designs for research. Chicago, IL: Rand McNally.

[cl21051-bib-0037] Clarke, R. V. (1997). Situational crime prevention: Successful case studies (2nd ed.). Monsey, NY: Criminal Justice Press.

[cl21051-bib-0038] Clarke, R. V. , & Weisburd, D. (1994). Diffusion of crime control benefits: Observations on the reverse of displacement. Crime Prevention Studies, 2, 165–184.

[cl21051-bib-0039] Cohen, J. (1988). Statistical power analysis for the behavioral sciences (2nd ed.). Hillsdale, NJ: Lawrence Erlbaum.

[cl21051-bib-0040] Cook, P. J. (1980). Research in criminal deterrence: Laying the groundwork for the second decade. Crime and Justice, 2, 211–268.

[cl21051-bib-0041] Cook, P. J. , & Ludwig, J. (2006). Aiming for evidence‐based gun policy. Journal of Policy Analysis and Management, 25(3), 691–735.

[cl21051-bib-0042] Cook, P. J. , Ludwig, J. , Venkatesh, S. , & Braga, A. A. (2007). Underground gun markets. The Economic Journal, 117(524), F588–F618.

[cl21051-bib-0043] Corsaro, N. (2013). The high point drug market intervention: Examining impact across target areas and offense types. Victims & Offenders, 8(4), 416–445.

[cl21051-bib-0044] Corsaro, N. , & McGarrell, E. F. (2009). Testing a promising homicide reduction strategy: Re‐assessing the impact of the Indianapolis “pulling levers” intervention. Journal of Experimental Criminology, 5(1), 63–82.

[cl21051-bib-0045] Dalton, E. (2002). Targeted crime reduction efforts in ten communities: Lessons for the Project Safe Neighborhoods initiative. U.S. Attorney's Bulletin, 50, 16–25.

[cl21051-bib-0046] Deuchar, R. (2013). Policing youth violence: Transatlantic connections. London: IEP Press.

[cl21051-bib-0047] Durlauf, S. N. , & Nagin, D. S. (2011). Imprisonment and crime: Can both be reduced? Criminology and Public Policy, 10(1), 13–54.

[cl21051-bib-0048] Duval, S. J. (2005). The trim and fill method. In H. R. Rothstein , A. J. Sutton , & M. Borenstein (Eds.), Publication bias in meta‐analysis: Prevention, assessment, and adjustments (pp. 127–144). Chichester, England: Wiley.

[cl21051-bib-0049] Duval, S. , Tweedie, R. , Duval, S. , & Tweedie, R. (2000). A nonparametric “trim and fill” method of accounting for publication bias in meta‐analysis. Journal of the American Statistical Association, 95(449), 89–98.

[cl21051-bib-0050] Eck, J. (2002). Preventing crime at places. In Sherman, L. W. , Farrington, D. , Welsh, B. , & MacKenzie, D. L. (Eds.), Evidence‐based crime prevention (pp. 241–294). New York, NY: Routledge.

[cl21051-bib-0051] Fagan, J. (2002). Policing guns and youth violence. The Future of Children, 12(2), 132–151. 12194607

[cl21051-bib-0052] Farrington, D. P. , Gill, M. , Waples, S. J. , & Argomaniz, J. (2007). The effects of closed‐circuit television on crime: Meta‐analysis of an English national quasi‐experimental multi‐site evaluation. Journal of Experimental Criminology, 3(1), 21–38.

[cl21051-bib-0053] Farrington, D. P. , Gottfredson, D. C. , Sherman, L. W. , & Welsh, B. C. (2002). The Maryland scientific methods scale. In L. W. Sherman , D. P. Farrington , B. C. Welsh , & D. L. MacKenzie (Eds.), Evidence‐based crime prevention (pp. 13–21). New York, NY: Routledge.

[cl21051-bib-0054] Farrington, D. P. , & Petrosino, A. (2001). The Campbell collaboration crime and justice group. The Annals of the American Academy of Political and Social Science, 578, 35–49.

[cl21051-bib-0055] Farrington, D. P. , Ttofi, M. M. , & Lösel, F. (2016). Developmental and social prevention. In D. Weisburd , D. P. Farrington , & C. Gill (Eds.), What works in crime prevention and rehabilitation: Lessons from systematic reviews (pp. 15–75). New York, NY: Springer.

[cl21051-bib-0056] Gibbs, J. P. (1975). Crime, punishment, and deterrence. New York, NY: Elsevier.

[cl21051-bib-0057] Gravel, J. , Bouchard, M. , Descormiers, K. , Wong, J. S. , & Morselli, C. (2013). Keeping promises: A systematic review and a new classification of gang control strategies. Journal of Criminal Justice, 41(4), 228–242.

[cl21051-bib-0058] Hamilton, B. , Rosenfeld, R. , & Levin, A. (2018). Opting out of treatment: Self‐selection bias in a randomized controlled study of a focused deterrence notification meeting. Journal of Experimental Criminology, 17(1), 1–17.

[cl21051-bib-0059] Hasselblad, V. , & Hedges, L. V. (1995). Meta‐analysis of screening and diagnostic tests. Psychological Bulletin, 117(1), 167–178. 7870860 10.1037/0033-2909.117.1.167

[cl21051-bib-0060] Horney, J. , & Marshall, I. H. (1992). Risk perceptions among serious offenders: The role of crime and punishment. Criminology, 30(4), 575–594.

[cl21051-bib-0061] Kennedy, D. M. (2009). Drugs, race, and common ground: Reflections on the high point intervention. National Institute of Justice Journal, 262, 12–17.

[cl21051-bib-0062] Kennedy, D. M. (2006). Old wine in new bottles: Policing and the lessons of pulling levers. In D. Weisburd , & A. A. Braga (Eds.), Police innovation: Contrasting perspectives (pp. 155–170). New York, NY: Cambridge University Press.

[cl21051-bib-0063] Kennedy, D. M. (2008). Deterrence and crime prevention: Reconsidering the prospect of sanction. London, UK: Routledge Press.

[cl21051-bib-0064] Kennedy, D. M. (2011). Don't shoot: One man, a street fellowship, and the end of violence in inner‐city America. New York, NY: Bloomsbury.

[cl21051-bib-0065] Kennedy, D. M. , Piehl, A. M. , & Braga, A. A. (1996). Youth violence in Boston: Gun markets, serious youth offenders, and a use‐reduction strategy. Law and Contemporary Problems, 59(1), 147–196.

[cl21051-bib-0066] Kennedy, D. M. , & Wong, S. (2009). The High Point drug market intervention strategy. Washington, DC: U.S. Department of Justice, Office of Community Oriented Policing Services.

[cl21051-bib-0067] Koper, C. S. , & Mayo‐Wilson, E. (2012). Police strategies for reducing illegal possession and carrying of firearms. Campbell Systematic Reviews, 8, 1–53.

[cl21051-bib-0068] Koper, C. S. , Woods, D. J. , & Kubu, B. E. (2013). Gun violence prevention practices among local police in the United States. Policing: An International Journal of Police Strategies & Management, 36(3), 577–603.

[cl21051-bib-0069] KTVU (2013). *Oakland crime strategy has failed in past*. Interview with Barry Krisberg. Retrieved from inthenews.berkeleylawblogs.org/2013/12/19/oakland‐crime‐strategy‐has‐failed‐in‐past/

[cl21051-bib-0070] Lipsey, M. W. (2000). Statistical conclusion validity for intervention research: A significant (*p*< .05) problem. In L. Bickman (Ed.), Validity and social experimentation: Donald Campbell's legacy (Vol. 1, pp. 101–120). Thousand Oaks, CA: Sage Publications.

[cl21051-bib-0071] Lipsey, M. W. (2003). Those confounded moderators in meta‐analysis: Good, bad, and ugly. The Annals of the American Academy of Political and Social Science, 587, 69–81.

[cl21051-bib-0072] Lipsey, M. W. , & Wilson, D. B. (2001). Practical meta‐analysis, Applied Social Research Methods Series (Vol. 49). Thousand Oaks, CA: Sage Publications.

[cl21051-bib-0073] Lösel, F. , & Köferl, P. (1989). Evaluation research on correctional treatment in West Germany: A meta‐analysis. In H. Wegener , F. Lösel , & J. Haisch (Eds.), Criminal behavior and the justice system (pp. 334–355). New York, NY: Springer.

[cl21051-bib-0074] Ludwig, J. (2005). Better gun enforcement, less crime. Criminology & Public Policy, 4(4), 677–716.

[cl21051-bib-0075] Ludwig, J. , Kling, J. R. , & Mullainathan, S. (2011). Mechanism experiments and policy evaluations. Journal of Economic Perspectives, 25(3), 17–38.

[cl21051-bib-0076] MacKenzie, D. L. , & Hickman, L. J. (1998). *What works in corrections*? Report to the State of Washington Joint Audit and Review Committee, College Park, MD: University of Maryland, Department of Criminology and Criminal Justice.

[cl21051-bib-0077] Mazerolle, L. , Soole, D. W. , & Rombouts, S. (2007). Street level drug law enforcement: A meta‐analytic review. Campbell Systematic Reviews, 3, 1–47.

[cl21051-bib-0078] McGarrell, E. F. , Hipple, N. K. , Bynum, T. S. , Perez, H. , Gregory, K. , Kane, C. M. , & Ransford, C. (2013). Promising strategies for violence reduction: Lessons from two decades of innovation. East Lansing, MI: Michigan State University, School of Criminal Justice.

[cl21051-bib-0079] Morgan, S. L. , & Winship, C. (2007). Counterfactuals and causal inference: Methods and principals for social research. New York, NY: Cambridge University Press.

[cl21051-bib-0080] Nagin, D. S. (1998). Criminal deterrence research at the outset of the twenty‐first century. Crime and Justice, 23, 1–42.

[cl21051-bib-0081] Nagin, D. S. , & Telep, C. W. (2017). Procedural justice and legal compliance. Annual Review of Law and Social Science, 13, 5–28.

[cl21051-bib-0082] National Network for Safe Communities (2013). Group violence intervention: An implementation guide. Washington, DC: U.S. Department of Justice, Office of Community Oriented Policing Services.

[cl21051-bib-0083] National Research Council (2004). Fairness and effectiveness in policing: The evidence committee to review research on police policy and practices. Washington, DC: National Academies Press.

[cl21051-bib-0084] National Research Council (2005). Firearms and violence: A critical review, Committee to Improve Research Information and Data on Firearms. Washington, DC: The National Academies Press.

[cl21051-bib-0085] Paternoster, R. (1987). The deterrent effect of the perceived certainty and severity of punishment: A review of the evidence and issues. Justice Quarterly, 4(2), 173–217.

[cl21051-bib-0086] Paternoster, R. , Brame, R. , Bachman, R. , & Sherman, L. W. (1997). Do fair procedures matter? The effect of procedural justice on spouse assault. Law & Society Review, 31(1), 163–204.

[cl21051-bib-0087] Perry, A. E. , & Johnson, M. (2008). Applying the consolidated standards of reporting trials (CONSORT) to studies of mental health provision for juvenile offenders: A research note. Journal of Experimental Criminology, 4(2), 165–185.

[cl21051-bib-0088] Perry, A. E. , Weisburd, D. , & Hewitt, C. (2010). Are criminologists describing randomized controlled trials in ways that allow us to assess them? Findings from a sample of crime and justice trials. Journal of Experimental Criminology, 6(3), 245–262.

[cl21051-bib-0089] Petrosino, A. , Campie, P. , Pace, J. , Fronius, T. , Guckenburg, S. , Wiatrowski, M. , & Rivera, L. (2015). Cross‐sector, multi‐agency interventions to address urban youth firearms violence: A rapid evidence assessment. Aggression and Violent Behavior, 22(1), 87–96.

[cl21051-bib-0090] Piehl, A. M. , Cooper, S. J. , Braga, A. A. , & Kennedy, D. M. (2003). Testing for structural breaks in the evaluation of programs. Review of Economics and Statistics, 85(3), 550–558.

[cl21051-bib-0091] President's Task Force on 21st Century Policing (2015). Final report of the President's Task Force on 21^st^ Century Policing. Washington, DC: Office of Community Oriented Policing Services.

[cl21051-bib-0092] Reppetto, T. A. (1976). Crime prevention and the displacement phenomenon. Crime & Delinquency, 22(2), 166–177.

[cl21051-bib-0093] Rosenfeld, R. , Fornango, R. , & Baumer, E. (2005). Did Ceasefire. Criminology & Public Policy, 4(3), 419–449.

[cl21051-bib-0094] Rosenthal, R. (1994). Parametric measures of effect size. In H. Cooper , & L. V. Hedges (Eds.), The handbook of research synthesis (pp. 231–244). New York, NY: Russell Sage.

[cl21051-bib-0095] Rossi, P. H. (1987). The iron law of evaluation and other metallic rules. Research in Social Problems and Public Policy, 4, 3–20.

[cl21051-bib-0096] Rothstein, H. R. (2008). Publication bias as a threat to the validity of meta‐analytic results. Journal of Experimental Criminology, 4(1), 61–81.

[cl21051-bib-0097] Rothstein, H. R. , & Hopewell, S. (2009). The grey literature. In H. Cooper , L. V. Hedges , & J. C. Valentine (Eds.), The handbook on research synthesis (2nd ed.). New York, NY: Sage Publications. (103‐126).

[cl21051-bib-0098] Sampson, R. J. (1997). Neighborhoods and violent crime: A multilevel study of collective efficacy. Science, 277(5328), 918–924. 9252316 10.1126/science.277.5328.918

[cl21051-bib-0099] Saunders, J. , Lundberg, R. , Braga, A. A. , Ridgeway, G. , & Miles, J. (2015). A synthetic control approach to evaluating place‐based crime interventions. Journal of Quantitative Criminology, 31(3), 413–434.

[cl21051-bib-0100] Saunders, J. , Ober, A. J. , Kilmer, B. , & Greathouse, S. M. (2016). A community‐based, focused‐deterrence approach to closing overt drug markets: A process and fidelity evaluation of seven sites appendix G. Santa Monica, CA: RAND Corporation.

[cl21051-bib-0101] Seabrook, J. (2009). Don't shoot: A radical approach to the problem of gang violence. *The New Yorker*, June 22.

[cl21051-bib-0102] Shadish, W. R. , Cook, T. D. , & Campbell, D. T. (2002). Experimental and quasi‐experimental designs for generalized causal inference. New York, NY: Houghton Mifflin.

[cl21051-bib-0103] Sherman, L. W. (2002). Fair and effective policing. In J. Q. Wilson , & J. Petersilia (Eds.), Crime: Public policies for crime control (pp. 383–412). Oakland, CA: ICS Press.

[cl21051-bib-0104] Sherman, L. W. , Gottfredson, D. , MacKenzie, D. , Eck, J. , Reuter, P. , & Bushway, S. (1997). Preventing crime: What works, what doesn't and what's promising. Washington, DC: U.S. Department of Justice.

[cl21051-bib-0105] Spelman, W. , & Brown, D. K. (1984). Calling the police: Citizen reporting of serious crime. Washington, DC: U.S. Department of Justice, National Institute of Justice.

[cl21051-bib-0106] Simonsohn, U. , Nelson, L. D. , & Simmons, J. P. (2014). *P*‐curve: A key to the file‐drawer. Journal of Experimental Psychology: General, 143(2), 534–547. 23855496 10.1037/a0033242

[cl21051-bib-0107] Telep, C. W. , & Weisburd, D. (2012). What is known about the effectiveness of police practices in reducing crime and disorder? Police Quarterly, 15(4), 331–357.

[cl21051-bib-0108] The New York Times (2001). Text of Mayor Giuliani's farewell address. *The New York Times*, December 21. Retrieved from https://www.nytimes.com/2001/12/27/nyregion/text‐of‐mayor‐giulianis‐farewell‐address.html

[cl21051-bib-0109] Tyler, T. R. (2004). Enhancing police legitimacy. The Annals of the American Academy of Political and Social Science, 593, 84–99.

[cl21051-bib-0110] Tyler, T. R. (2006). Why people obey the law. Princeton, NJ: Princeton University Press.

[cl21051-bib-0111] Visher, C. , & Weisburd, D. (1998). Identifying what works: Recent trends in crime. Crime, Law, and Social Change, 28(3–4), 223–242.

[cl21051-bib-0112] Wallace, D. , Papachristos, A.V. , Meares, T. , & Fagan, J. (2016). Desistance and legitimacy: The impact of offender notification meetings on recidivism among high risk offenders. Justice Quarterly, 33(7), 1–28.

[cl21051-bib-0113] Weisburd, D. , Petrosino, A. , & Mason, G. (1993). Design sensitivity in criminal justice experiments. Crime and Justice, 17, 337–379.

[cl21051-bib-0114] Weisburd, D. (2008). Place‐based policing, Ideas in American Policing. Washington, DC: The Police Foundation.

[cl21051-bib-0115] Weisburd, D. , & Eck, J. E. (2004). What can police do to reduce crime, disorder and fear? The Annals of the American Academy of Political and Social Science, 593, 42–65.

[cl21051-bib-0116] Weisburd, D. , Lum, C. M. , & Petrosino, A. (2001). Does research design affect study outcomes in criminal justice? The Annals of the American Academy of Political and Social Science, 578, 50–70.

[cl21051-bib-0117] Weisburd, D. , & Majmundar, M. K. (Eds.). (2018). Proactive policing: Effects on crime and communities, Committee on Proactive Policing: Effects on Crime, Communities, and Civil Liberties. Washington, DC: The National Academies Press.

[cl21051-bib-0118] Weisburd, D. , Telep, C. W. , Hinkle, J. C. , & Eck, J. E. (2008). The effects of problem‐oriented policing on crime and disorder. Campbell Systematic Reviews, 4, 1–87. 10.1002/cl2.1005 PMC8356314 37133254

[cl21051-bib-0119] Welsh, B. C. , Peel, M. E. , Farrington, D. P. , Elffers, H. , & Braga, A. A. (2011). Research design influence on study outcomes in crime and justice: A partial replication with public area surveillance. Journal of Experimental Criminology, 7(2), 183–198.

[cl21051-bib-0120] Werb, D. , Rowell, G. , Guyatt, G. , Kerr, T. , Montaner, J. , & Wood, E. (2011). Effect of drug law enforcement on drug market violence: A systematic review. International Journal of Drug Policy, 22(1), 87–94. 21392957 10.1016/j.drugpo.2011.02.002

[cl21051-bib-0121] Wilkinson, L. (1999). Statistical methods in psychology journals: Guidelines and explanations. American Psychologist, 54(8), 594–604.

[cl21051-bib-0122] Wilson, D. B. (2009). Missing a critical piece of the pie: Simple document search strategies inadequate for systematic reviews. Journal of Experimental Criminology, 5(4), 429–440.

[cl21051-bib-0123] Zimring, F. E. , & Hawkins, G. (1973). Deterrence: The legal threat in crime control. Chicago, IL: University of Chicago Press.

